# Alcohol intake reprograms hepatic immune-metabolic circuits to exacerbate murine atherosclerosis and human cardiovascular risk

**DOI:** 10.1016/j.jhepr.2026.101932

**Published:** 2026-06-19

**Authors:** Constanze Hoebinger, Georg Semmler, Oleksandr Petrenko, Dragana Rajcic, Dorothea Katharina Hoffelner, Tereza Duckova, Taras Baranovskyi, Laura Goederle, My Phung Khuu, Christoph Reinhardt, Petra Pjevac, Joana Séneca, Kamil Mieczkowski, Katharina Burger, Ina Bergheim, Sophie Gensluckner, Andreas Völkerer, Thomas Reiberger, Bernhard Scheiner, Elmar Aigner, Bernhard Wernly, Christian Datz, Christoph J. Binder, Tim Hendrikx

**Affiliations:** 1 Department of Laboratory Medicine, KILM, Medical University of Vienna, Vienna, Austria; 2 Division of Gastroenterology and Hepatology, Department of Medicine III, Medical University of Vienna, Vienna, Austria; 3 Clinical Research Group MOTION, Medical University of Vienna, Vienna, Austria; 4 FLASH Centre for Liver Research, Department of Gastroenterology and Hepatology, Odense University Hospital, Odense, Denmark; 5 Institute of Clinical Research, Faculty of Health Sciences, University of Southern Denmark, Odense, Denmark; 6 Ukrainian Institute for Systems Biology and Medicine, Kyiv, Ukraine; 7 Center for Thrombosis and Hemostasis (CTH), University Medical Center of the Johannes Gutenberg-University Mainz, Mainz, Germany; 8 Research Center for Immunotherapy (FZI), University Medical Center Mainz, Johannes Gutenberg-University Mainz, Mainz, Germany; 9 Joint Microbiome Facility of the Medical University of Vienna and the University of Vienna, Vienna, Austria; 10 Division of Microbial Ecology, Centre for Microbiology and Environmental Systems Science, University of Vienna, Vienna, Austria; 11 Department of Nutritional Sciences, Molecular Nutritional Science, University of Vienna, Vienna, Austria; 12 First Department of Medicine, Paracelsus Medical University Salzburg, Salzburg, Austria; 13 Department of Internal Medicine, General Hospital Oberndorf, Teaching Hospital of the Paracelsus Medical University Salzburg, Oberndorf, Austria

**Keywords:** MetALD, ethanol, cardiovascular disease, uric acid, NLRP3 inflammasome

## Abstract

**Background & Aims:**

Recent reclassification of steatotic liver disease (SLD) distinguishes metabolic dysfunction-associated steatotic liver disease (MASLD) from MetALD, a newly defined entity combining MASLD with alcohol consumption. Since the mechanisms linking alcohol consumption in the context of SLD to cardiovascular disease (CVD) – the leading cause of SLD mortality – remain elusive, we investigated how metabolic dysregulation and alcohol intake synergistically promote atherosclerosis.

**Methods:**

Low-density lipoprotein receptor-deficient (*Ldlr*^*-/-*^) mice were fed a high-fat, high-cholesterol (HFC) diet with regular or ethanol-containing drinking water (10–20% v/v). Germ-free and antibiotic-treated *Ldlr*^*-/-*^ mice were used to assess the contribution of ethanol-induced dysbiosis. Associations between alcohol consumption and cardiometabolic risk were assessed in two human cohorts (n = 5,115, n = 2,515).

**Results:**

Ethanol intake in HFC diet-fed *Ldlr*^*-/-*^ mice exacerbated hepatic steatosis and systemic dyslipidemia, despite only modest elevations in systemic ethanol levels (*p* values ≤0.05). Liver transcriptomic profiling revealed ethanol-induced alterations in lipid metabolism and enhanced proinflammatory signatures, accompanied by increased recruitment of Ly6C^high^ monocytes to the liver (*p =* 0.0121) and elevated levels in the circulation (*p =* 0.0043). Correspondingly, ethanol-consuming HFC diet-fed *Ldlr*^*-/-*^ mice developed enlarged aortic root lesions (*p =* 0.0105). Neither germ-free conditions nor antibiotic treatment mitigated CVD progression. Metabolomic profiling revealed hyperuricemia in ethanol-exposed HFC diet-fed *Ldlr*^*-/-*^ mice, which was associated with upregulation of inflammasome-related genes in the liver, along with an increase in hepatic NLRP3 protein expression (*p* values ≤0.05). Notably, human data mirrored these findings, demonstrating a dose-dependent association between alcohol intake, dyslipidemia, monocytosis, hyperuricemia, and increased cardiovascular risk (*p* values ≤0.05).

**Conclusion:**

Our findings identify alcohol as an important immunomodulatory lifestyle factor that contributes to elevated cardiovascular risk.

**Impact and implications:**

Alcohol consumption, even at moderate levels, contributes to cardiovascular risk, particularly in individuals with steatotic liver disease (SLD). Combining murine models with human cohort data, we show that alcohol intake in SLD is associated with coordinated immune-metabolic alterations, including dyslipidemia, monocytosis, hyperuricemia, and enhanced NLRP3 inflammasome signaling, collectively indicating an elevated cardiovascular risk profile. These findings are relevant to hepatology and cardiovascular medicine, linking alcohol consumption to measurable systemic pathways beyond liver injury. Clinically, systematic assessment of alcohol intake and incorporation into cardiovascular risk evaluation may improve risk stratification and support closer surveillance and preventive strategies in patients with SLD.

## Highlights

• Ethanol plus HFC feeding in *Ldlr*^*-/-*^ mice accelerates early atherosclerosis.
• In HFC-fed *Ldlr*^*-/-*^ mice, ethanol exacerbates hepatic and systemic dyslipidemia and induces pro-atherogenic monocytosis.
• Ethanol induces hyperuricemia and NLRP3 inflammasome signaling in HFC-fed *Ldlr*^*-/-*^ mice, revealing a targetable pathway.
• Individuals with SLD show concordant, dose-dependent associations between alcohol intake and cardiometabolic risk.

## Graphical abstract

**Figure:**
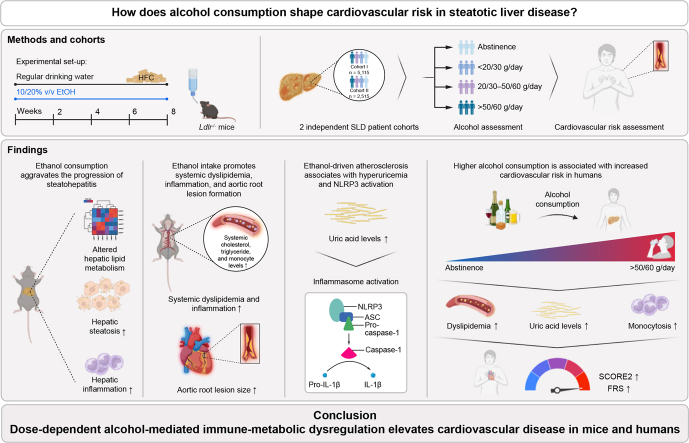

## Introduction

The updated nomenclature for steatotic liver disease (SLD) distinguishes alcohol-associated liver disease (ALD) from metabolic dysfunction-associated steatotic liver disease (MASLD), which together represent the most prevalent chronic liver diseases worldwide, affecting over one-third of the global population.^1^^,^^2^ MASLD is characterized by metabolic dysfunction, including dyslipidemia, obesity, and type 2 diabetes that drive disease progression, while ALD is defined by high alcohol intake, irrespective of cardiometabolic risk factors. Nonetheless, both conditions share similar pathophysiological mechanisms and exhibit comparable histopathological features, including steatosis, steatohepatitis, fibrosis, and cirrhosis.^3^ To capture their overlap, the updated classification introduced a new diagnostic category, MetALD, defined as MASLD with moderate alcohol intake (140-350 g/week for women; 210-420 g/week for men).^4^^,^^5^

The intersection of metabolic dysfunction and alcohol consumption is supported by epidemiological evidence demonstrating increased morbidity and mortality associated with alcohol intake in patients with metabolic disorders, such as MASLD.^6^^,^^7^ Nonetheless, alcohol consumption remains an underestimated risk factor. Studies indicate that 74% of Europeans consume alcohol, with nearly 10% drinking daily.^8^ With projections suggesting that 55% of the population may develop SLD by 2040,^9^ understanding the synergistic effects between metabolic dysfunction and alcohol exposure, as seen in MetALD, is essential.^10^^,^^11^

Metabolic dysregulation creates a pro-atherogenic milieu characterized by dyslipidemia, inflammation, oxidative stress, insulin resistance, and endothelial dysfunction, rendering cardiovascular diseases (CVDs) the leading cause of mortality among patients with MASLD.^12^ Increased *de novo* lipogenesis, a hallmark of MASLD, elevates saturated fatty acid levels and contributes to dyslipidemia, characterized by elevated plasma cholesterol, triglycerides, and low-density lipoprotein cholesterol, together with reduced high-density lipoprotein cholesterol.^13^ Hepatic lipid accumulation further triggers inflammatory and oxidative cascades that accelerate atherosclerosis.^14^ In ALD, excessive ethanol consumption can further exacerbate dyslipidemia and thereby augment atherogenesis.[15], [16], [17] However, the effects of alcohol consumption on CVD in MetALD and the underlying mechanisms remain unclear, underscoring the need to investigate how alcohol contributes to cardiovascular risk in these patients.^11^^,^^18^

In this study, we demonstrate that ethanol consumption combined with a high-fat, high-cholesterol (HFC) diet in *Ldlr*^*-/-*^ mice, which display a human-like lipoprotein profile, induces systemic dyslipidemia, inflammation, hyperuricemia, and accelerates the progression of steatohepatitis. These metabolic alterations subsequently accelerate atherosclerosis development and plaque progression. Our findings indicate that disease aggravation may be linked to elevated uric acid levels and NLRP3 inflammasome-related signaling, independent of ethanol-mediated effects on the intestinal microbiota. Notably, we observed similar alterations in systemic lipid levels and inflammatory markers upon alcohol consumption in humans. These findings identify individuals with underlying SLD who consume alcohol as having an increased risk of experiencing cardiovascular events.

## Materials and methods

### Murine models

Female *Ldlr*^*-/-*^ mice were used to model SLD and atherosclerosis. Accordingly, *Ldlr*^*-/-*^ mice were fed a HFC diet for 8 weeks and received either regular drinking water or ethanol-supplemented water, with concentrations alternating between 10% (v/v) for 3 days and 20% (v/v) for 4 days, provided *ad libitum* for 8 or 16 weeks. To assess microbiome-dependent effects, additional experiments were performed using broad-spectrum antibiotic treatment as well as germ-free and conventionally raised *Ldlr*^*-/-*^ mice. Blood, liver, intestine, stool, aorta, and heart were collected for analyses. Further information can be found in the supplementary materials and methods.

### Human cohorts

Two independent human cohorts were analyzed. Cohort I included 5,115 individuals aged 40-69 years undergoing first screening colonoscopy within the prospective SAKKOPI registry, and Cohort II comprised 2,515 first-time referrals to a hepatology outpatient clinic. In both cohorts, self-reported alcohol intake was converted to daily ethanol consumption and used to stratify participants into abstainers and low (<20 g/day for women, <30 g/day for men), moderate (20–50 g/day for women, 30–60 g/day for men), or high (>50 g/day for women, >60 g/day for men) alcohol consumption groups. Patients with liver diseases other than SLD were excluded. Cardiovascular risk was assessed using SCORE2 and the Framingham risk score. Laboratory parameters were compared across alcohol consumption groups using appropriate statistical tests. Subgroup analyses excluding statin users were additionally performed (see supplementary materials and methods for further information).

### Biochemical analyses and statistics

Murine plasma and tissues were analyzed using biochemical assays, ELISA, quantitative PCR, western blotting, microscopy, (immuno)histochemistry, and flow cytometry to assess metabolic, inflammatory, and vascular parameters. Biochemical assays included the quantification of plasma and hepatic lipids (cholesterol, triglycerides, free fatty acids), as well as uric acid, ethanol, endotoxin (lipopolysaccharide), cytokines, and immunoglobulins. Atherosclerotic lesion burden was assessed in the aortic root and by *en face* analysis of the aorta. In addition, liver transcriptomics, plasma metabolomics, and fecal 16S rRNA sequencing were performed to characterize ethanol-associated immune-metabolic alterations. Additional details are provided in the supplementary materials and methods.

## Results

### Ethanol feeding aggravates systemic dyslipidemia and hepatic steatosis by altering lipid metabolism in the liver of *Ldlr*^*-/-*^ mice

**Fig. 1:**
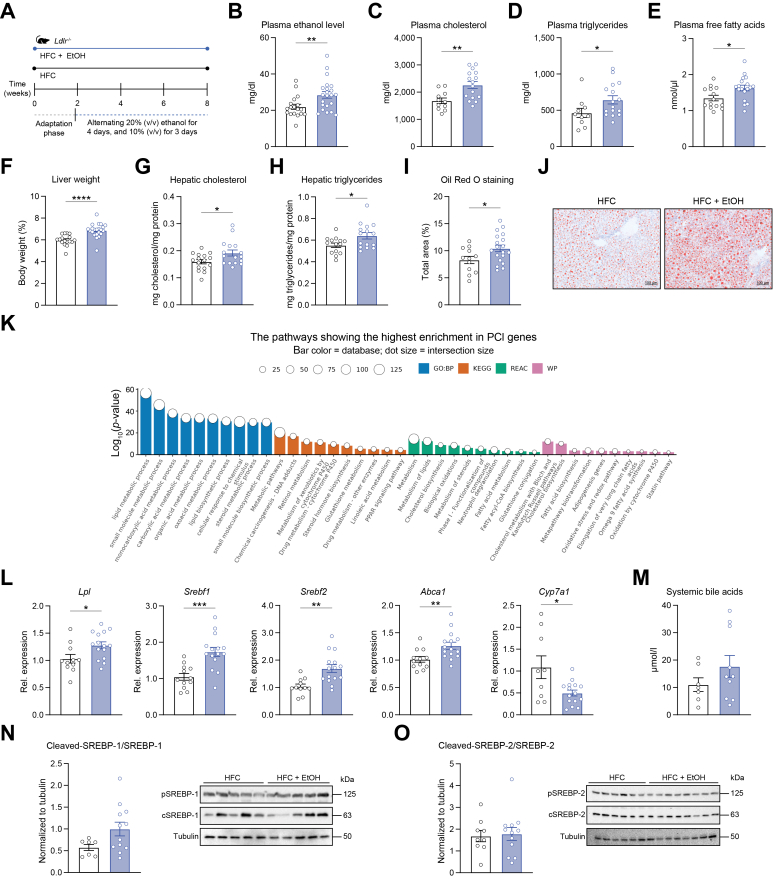
Ethanol intake exacerbates dyslipidemia and alters hepatic lipid metabolism in *Ldlr*^*-/-*^ mice fed a HFC diet. (A) Study design. (B) Plasma ethanol levels. (C-E) Plasma lipid levels. (F) Liver weight. (G-H) Hepatic lipid levels. (I) Quantification and (J) representative images of Oil Red O staining of liver sections. (K) Significant pathways showing the highest enrichment score (up to the top 10) with their respective significance values. Colors indicate database source, and bubble size the degree of overlap between the annotated pathway and the query gene set. (L) Hepatic gene expression. (M) Systemic bile acid levels. (N-O) Cleaved SREBP-1 and SREBP-2 protein levels. Data are mean ± SEM (n = 4–18 for the HFC group; n = 6–22 for the HFC+EtOH group). mRNA was normalized to *18S* and expressed relative to HFC-fed *Ldlr*^*-/-*^ mice receiving regular water. Statistical significance was determined using a two-tailed, unpaired Student’s *t* test. ∗*p ≤*0.05, ∗∗*p ≤*0.01, ∗∗∗∗*p ≤*0.0001. EtOH, ethanol; HFC, high-fat, high-cholesterol.

To study the impact of alcohol consumption on CVD progression, *Ldlr*^*-/-*^ mice were fed a HFC diet for 8 weeks and received either regular drinking water or ethanol-supplemented drinking water (Fig. 1A). Notably, ethanol intake resulted in only a modest increase in plasma ethanol levels compared with controls (Fig. 1B), thereby distinguishing our model from murine ALD, where systemic ethanol levels are reported to be up to ten-fold higher.^19^ Body weight gain was comparable between groups (Fig. S1A), and hepatic expression of *Cyp2e1* and *Adh1*, two key enzymes in ethanol metabolism, was unchanged or only modestly altered in ethanol-fed mice (Fig. S1B and C).

Furthermore, ethanol intake aggravated systemic dyslipidemia in HFC-fed *Ldlr*^*-/-*^ mice, as indicated by increased plasma cholesterol, triglycerides, and free fatty acid levels after 8 weeks (Fig. 1C–E). While lipid quantification in the small intestinal content showed no difference in cholesterol levels, triglyceride levels were increased in HFC diet-fed *Ldlr*^*-/-*^ mice receiving ethanol compared with mice receiving regular drinking water (Fig. S1D and E). Small intestinal mRNA levels of genes involved in lipid uptake and export (*Npc1l1*, *Abcg4*, *Abcg8*, *Cd36*, *Fatp4*) remained unchanged (Fig. S1F). Yet, HFC diet-fed *Ldlr*^*-/-*^ mice drinking ethanol displayed increased liver-to-body weight ratio, accompanied by elevated cholesterol and triglyceride content in the liver, paralleled by histological evidence of increased macrosteatosis (Figs 1F-J and S1G).

Hepatic bulk RNA sequencing revealed distinct transcriptional profiles between ethanol-exposed and control mice (Fig. S1H). Under the defined thresholds, the two experimental groups had 219 differentially expressed genes (Fig. S1I), of which a selection was confirmed by qPCR analyses (Fig. 1L). The 500 genes with the largest absolute loadings on principal component 1, which captured most of the variance between groups, were significantly enriched for pathways related to lipid, cholesterol, and fatty acid turnover, suggesting that these transcriptional changes contribute to increased hepatic steatosis upon ethanol intake in HFC-fed *Ldlr*^*-/-*^ mice (Fig. 1K).

Targeted per-gene analyses of cholesterol-related signatures revealed that *Apoa4*, *Pcsk9*, *Soat1, and Lipa* exhibited the most pronounced expression differences, with higher expression in HFC-fed *Ldlr*^*-/-*^ mice receiving ethanol (Fig. S1J and K). While genes such as *Soat1* and *Lipa* were increased, suggesting enhanced intracellular cholesterol esterification and turnover, genes involved in cholesterol clearance were decreased in HFC-fed *Ldlr*^*-/-*^ mice subjected to ethanol feeding. In particular, *Lcat*, which esterifies free cholesterol into cholesteryl esters in HDL particles to promote reverse cholesterol transport, and *Cyp7a1*, the rate-limiting enzyme for bile acid synthesis, were markedly downregulated (Figs 1L and S1J,K). Yet, total circulating bile acid levels remained unchanged (Fig. 1M). Despite elevated transcriptional levels, mean hepatic cleaved SREBP-1 levels were only mildly elevated upon alcohol intake (Fig. 1N), while cleaved SREBP-2 was comparable between groups (Fig. 1O). These results indicate selective activation of the lipogenic SREBP-1 pathway in HFC-fed *Ldlr*^*-/-*^ mice upon ethanol intake, suggesting that ethanol primarily alters cholesterol clearance rather than enhancing cholesterol synthesis. Collectively, these findings imply that specific alterations in hepatic lipid homeostasis promote enhanced hepatic steatosis and systemic hyperlipidemia in ethanol-exposed HFC-fed *Ldlr*^*-/-*^ mice.

### HFC diet in combination with ethanol is associated with accelerated hepatic inflammation in *Ldlr*^*-/-*^ mice

**Fig. 2:**
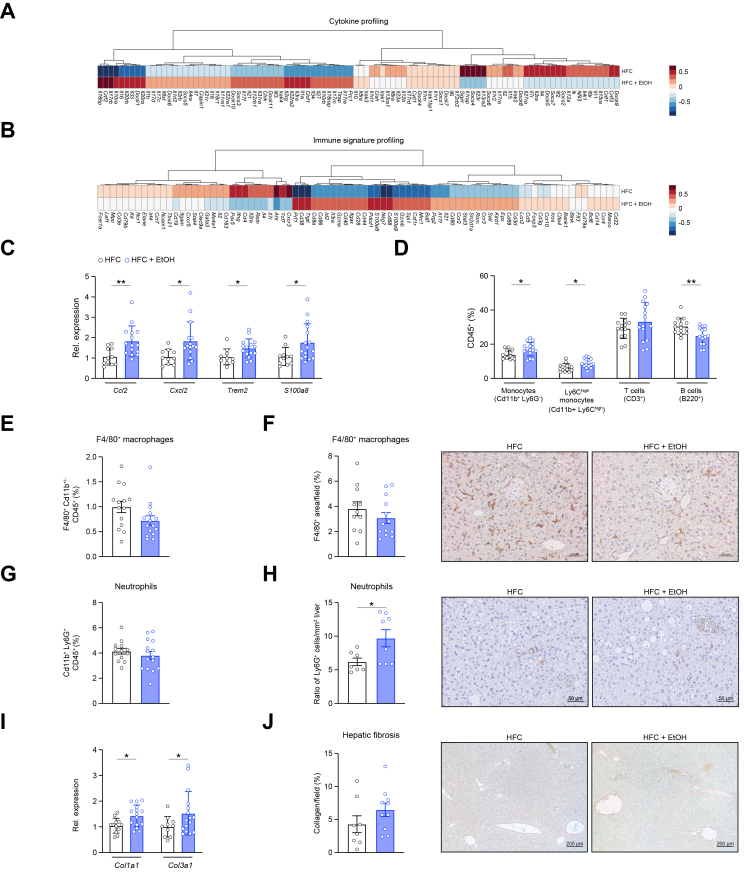
*Ldlr*^*-/-*^ mice exhibit accelerated hepatic inflammation following HFC diet combined with ethanol intake. (A) Heatmap showing the expression levels of cytokine genes from the BioMart database and (B) of immune-related genes in livers of HFC-fed *Ldlr*^*-/-*^ mice with regular and ethanol-containing drinking water. Abundance values are scaled and averaged per group. (C) Hepatic gene expression. (D) Quantification of hepatic immune cell populations by flow cytometry. (E) Quantification of hepatic Kupffer cells by flow cytometry. (F) Quantification and representative images of F4/80-stained liver sections. (G) Quantification of hepatic neutrophils by flow cytometry. (H) Quantification and representative images of Ly6G-stained liver sections. (I) Hepatic gene expression. (J) Quantification and representative images of Sirius Red-stained liver sections. Data are mean ± SEM (n = 7–14 for the HFC group and n = 8–16 for the HFC+EtOH group). mRNA was normalized to *18S* and expressed relative to HFC-fed *Ldlr*^*-/-*^ mice on regular water. Statistical significance was determined using a two-tailed unpaired Student’s *t* test; ∗*p ≤*0.05, ∗∗*p ≤*0.01. EtOH, ethanol; HFC, high-fat, high-cholesterol.

Immune profiling of the liver transcriptome dataset uncovered an altered cytokine signature in mice drinking ethanol (Figs 2A and S2A). *Il16*, *Il33*, and *Il1a* were among the most differentially upregulated cytokines in *Ldlr*^*-/-*^ mice receiving HFC diet and ethanol. Moreover, we observed increased expression of immune cell markers associated with macrophages and monocytes (*Cd68*, *Cd38*), cytotoxic T/NK cells (*Prf1*, *Tigit*, *Nkg7*), neutrophils (*S100a8, S100a9*), and B cells (*Cd22, Cd79a, Blnk, Bank1*) (Figs 2B and S2B). Conversely, markers characteristic of T helper 1 cells and regulatory T cells (*Ifng*, *Cxcr3*, *Il2ra*, *Ahr*) were suppressed (Figs 2B and S2B). Reactome pathway analysis further indicated that signatures of neutrophil degranulation, FcγR-dependent phagocytosis, and IL-10 signaling, among others, prevailed in mice with ethanol intake compared with controls (Fig. S2C).

Consistent with bulk RNA-seq data, gene expression of the monocyte-recruiting chemokine *Ccl2* and the macrophage-associated marker *Trem2* were elevated in the livers of HFC diet-fed *Ldlr*^*-/-*^ mice exposed to ethanol intake (Fig. 2C). These transcriptional changes were accompanied by a relative increase in infiltrating monocytes, particularly the proinflammatory Ly6C^high^ subset (Fig. 2D), whereas resident macrophages were modestly reduced (Fig. 2E,F). In line with signatures of neutrophil degranulation, mRNA levels of *Cxcl2*, a neutrophil-attracting chemokine, and *S100a8,* an alarmin predominantly produced by activated neutrophils and monocytes, were increased (Fig. 2C). Although relative hepatic neutrophil abundance was unchanged (Fig. 2G), absolute numbers of Ly6G^+^ neutrophils were elevated in ethanol-exposed mice (Fig. 2H). No difference in the relative amount of T cells was found in the liver (Fig. 2D), while hepatic B cells showed a relative decrease in HFC-fed *Ldlr*^*-/-*^ mice subjected to ethanol (Fig. 2D). Yet, circulating B cell numbers and plasma IgM, IgG1, or IgA levels remained unchanged (Figs S2D–F and S3C).

Evaluation of hepatic fibrosis revealed increased expression of profibrotic genes (*Col1a1*, *Col3a1*) in the liver (Fig. 2I), while collagen deposition was slightly higher in ethanol-fed *Ldlr*^*-/-*^ mice (Fig. 2J). Overall, these data indicate that in *Ldlr*^*-/-*^ mice, ethanol exposure combined with HFC diet feeding particularly aggravates the development of steatohepatitis.

### Ethanol intake in HFC diet-fed *Ldlr*^*-/-*^ mice exacerbates systemic inflammation and aortic lesion formation

**Fig. 3:**
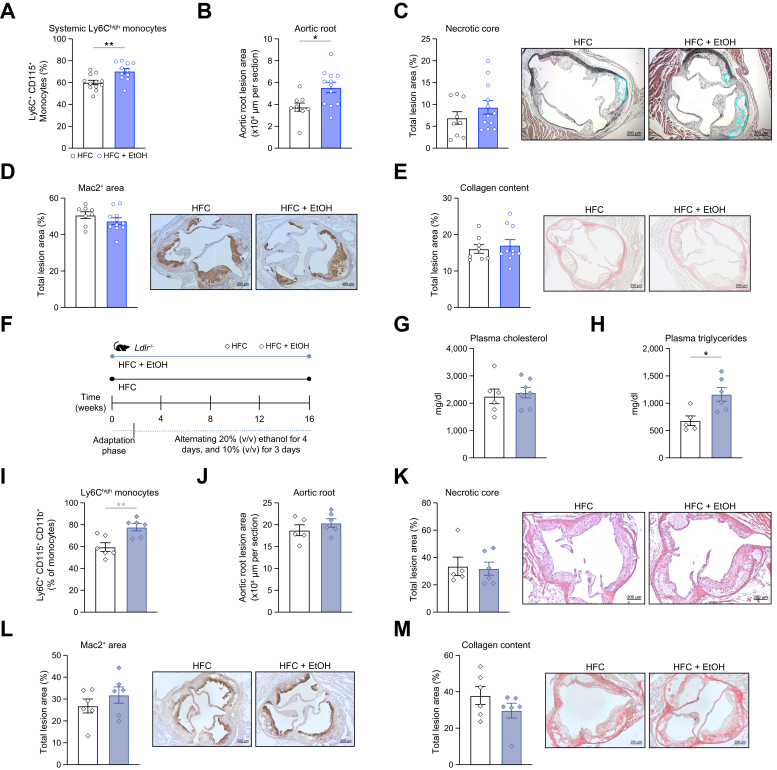
HFC diet with ethanol intake results in increased atherosclerosis in *Ldlr*^*-/-*^ mice. (A-H) 8-week dietary intervention. (A) Quantification of circulating Ly6C^high^ monocytes determined by flow cytometry. (B) Average aortic root lesion size expressed as μm^2^ per section. (C) Relative size of necrotic core expressed as a percentage of total plaque area and representative images of Masson’s trichrome-stained aortic root sections. (D) Quantification and representative images of infiltrating macrophages assessed by Mac2^+^ staining of aortic root sections. (E) Quantification and representative images of collagen content assessed by Sirius Red staining of aortic root sections. (F-M) 16 weeks dietary intervention. (F) Study design. (G-H) Plasma lipid levels. (I) Quantification of circulating Ly6C^high^ monocytes determined by flow cytometry. (J) Quantification of aortic root lesion size, calculated as the average lesion size (μm^2^) from seven sections throughout the aortic root. (K) Quantification of relative size of necrotic core expressed as a percentage of total plaque area and representative images of H&E-stained aortic root sections. (L) Quantification and representative images of infiltrating macrophages assessed by Mac2^+^ staining of aortic root sections. (M) Quantification and representative images of collagen content assessed by Sirius Red staining of aortic root sections. Data in (A-E) are mean ± SEM (n = 6–12 for the HFC group and n = 10–12 for the HFC+EtOH group). Data in (F–M) are mean ± SEM (n = 5–6 for the HFC group and n = 5–6 for the HFC+EtOH group). Statistical significance was determined using a two-tailed unpaired Student’s *t* test; ∗*p ≤*0.05, ∗∗*p ≤*0.01. EtOH, ethanol; HFC, high-fat, high-cholesterol.

Characterization of systemic effects of ethanol in combination with a HFC diet showed that ethanol administration resulted in elevated total monocytes and an increased monocyte-to-lymphocyte ratio, a parameter proposed to predict the severity of coronary artery disease (Table 1).^20^ Within the monocyte compartment, systemic proinflammatory Ly6C^high^ monocytes were specifically elevated in ethanol-supplemented mice (Figs 3A and S3A). Ethanol intake was further associated with elevated red blood cell counts, hemoglobin, and hematocrit, while mean corpuscular hemoglobin was reduced. Although total white blood cell counts were not significantly affected, relative lymphocyte abundance was decreased, while granulocytes and eosinophils were increased (Table 1). Strikingly, other hematological parameters and systemic immune cell composition did not show any significant changes (Table 1, Fig. S3B–E).

**Table 1:** Murine blood parameters after 8-week dietary intervention.

Parameter	HFC	HFC + EtOH	*p* values
White blood cells, 10^3^/μl	9.5 (2.5)	8.6 (2.1)	0.09126
Lymphocytes, 10^3^/μl	7.5 (2.2)	6 (1.3)	0.0712
Lymphocytes, %	79.9 (2.7)	71.2 (4.7)	**0.001**
Monocytes, 10^3^/μl	0.5 (0.3)	0.6 (0.2)	0.2351
Monocytes, %	5.8 (0.8)	7.0 (1.1)	**0.0113**
Granulocytes, 10^3^/μl	1.4 (0.3)	2.0 (0.9)	0.0744
Granulocytes, %	14.3 (2.9)	21.4 (5.1)	**0.0022**
Eosinophils, %	1.3 (0.2)	1.8 (0.6)	**0.0282**
Red blood cells, 10^6^/μl	16.4 (2.6)	12.9 (3.4)	**0.0211**
Hemoglobin, g/dl	23.3 (3.3)	18.8 (5)	**0.0298**
Hematocrit, %	83.0 (11.8)	67.2 (17.7)	**0.0337**
Mean corpuscular volume, μm^3^	50.7 (1.1)	52 (1.3)	**0.0287**
Mean corpuscular hemoglobin, pg	13.6 (0.9)	12.5 (0.5)	**0.0135**
Mean corpuscular hemoglobin concentration, d/dl	26.8 (1.5)	27.9 (1.0)	0.3853
Red blood cell distribution width, %	14.54 (0.9)	14.6 (0.8)	0.2083
Platelets, 10^3^/μl	981.6 (105.7)	838.5 (193.3)	0.0627
Mean platelet volume, μm^3^	5.9 (0.2)	6.0 (0.2)	0.0627
Monocyte/lymphocyte, ratio	0.09 (0.1)	0.09 (0.02)	**0.0181**

EtOH, ethanol; HFC, high-fat, high-cholesterol.

Statistical significance was determined using two-tailed, unpaired Student’s *t* tests.

Of note, ethanol administration markedly increased the aortic root lesion size in HFC diet-fed *Ldlr*^*-/-*^ mice compared with HFC diet-fed *Ldlr*^*-/-*^ mice receiving regular drinking water (Figs 3B and S3F), accompanied by only a modest increase in necrotic core size (Fig. 3C). Despite elevated monocytosis, the relative macrophage content within the cellular area of aortic root lesions was found to be comparable between the groups (Fig. 3D). Furthermore, collagen content within atherosclerotic lesions did not differ between the study groups (Fig. 3E). To determine whether accelerated atherosclerosis was attributable to systemic hypercholesterolemia, we compared lesion size in mice with matched cholesterol exposure and found that ethanol remained associated with larger lesions, indicating that ethanol-driven atherogenesis is at least partly independent of systemic cholesterol (Fig. S3G and H). Bold values indicate statistically significant differences with a p value ≤ 0.05.

After 16 weeks of dietary intervention (Fig. 3F), and consistent with findings from the 8-week setting, systemic ethanol levels were only modestly increased and body weight gain remained comparable between groups (Fig. S3I and J), yet hepatic steatosis progression was increased in HFC-fed *Ldlr*^*-/-*^ mice subjected to ethanol feeding (Fig. S3K-O). Notably, although plasma cholesterol levels were similar after 16 weeks of dietary intervention, circulating triglyceride levels were elevated in HFC diet-fed *Ldlr*^*-/-*^ mice upon ethanol intake (Fig. 3G,H). No major changes in hepatic immune cell composition or proinflammatory gene expression in the liver were observed between the groups (Fig. S3P,Q). Yet, ethanol intake in HFC diet-fed *Ldlr*^*-/-*^ mice resulted in an increase in circulating proinflammatory Ly6C^high^ monocytes (Fig. 3I, Table 2), despite no differences between groups in atherosclerotic lesion area, necrotic core size, or relative macrophage or collagen content within aortic root lesions (Fig. 3J-M). In summary, these findings suggest that ethanol intake significantly accelerates early atherosclerosis development, whereas this effect appears attenuated at later stages following prolonged exposure.

**Table 2:** Characteristics of 4,334 individuals in the general population (Cohort I) excluding statin users (n = 781, 15.3%), stratified by self-reported alcohol intake.

Parameter	Overall (N = 4,334)	Abstinence (n = 1,595)	<20/30 g/day (n = 2,284)	20/30-50/60 g/day (n = 418)	>50/60 g/day (n = 37)	*p* values
Age, years	58.7 (8.25)	59.5 (8.58)	57.9 (7.95)	59.7 (8.11)	60.3 (8.70)	**<0.001**
Male sex	2,199 (50.7%)	479 (30.0%)	1,434 (62.8%)	258 (61.7%)	28 (75.7%)	**<0.001**
BMI, kg/m^2^	26.9 (4.60)	27.4 (5.11)	26.7 (4.28)	26.4 (3.94)	28.2 (4.97)	**<0.001**
BMI ≥30 kg/m^2^	932 (21.5%)	428 (26.8%)	423 (18.5%)	70 (16.7%)	11 (29.7%)	**<0.001**
Abdominal obesity	3,145 (72.6%)	1,214 (76.1%)	1,593 (69.7%)	308 (73.7%)	30 (81.1%)	**<0.001**
Alcohol, g/week	20.0 [0-100]	0 [0-0]	80.0 [20.0-100]	280 [160-280]	500 [440-560]	**<0.001**
Diabetes	586 (13.5%)	242 (15.2%)	268 (11.7%)	68 (16.3%)	8 (21.6%)	**0.005**
Uric acid, mg/dl	5.70 (1.51)	5.39 (1.44)	5.81 (1.49)	6.11 (1.56)	6.90 (2.35)	**<0.001**
AST, U/L	20.0 [17.0-25.0]	20.0 [17.0-24.0]	21.0 [17.0-25.0]	22.0 [18.0-27.0]	22.0 [20.0-29.0]	**<0.001**
AST xULN	0.486 [0.400-0.600]	0.514 [0.429-0.629]	0.480 [0.400-0.600]	0.520 [0.420-0.629]	0.486 [0.420-0.600]	**<0.001**
ALT, U/L	20.0 [15.0-29.0]	19.0 [14.0-26.0]	21.0 [16.0-29.0]	23.0 [16.0-32.0]	23.0 [17.0-30.0]	**<0.001**
ALT xULN	0.486 [0.371-0.660]	0.486 [0.371-0.680]	0.480 [0.371-0.657]	0.514 [0.380-0.720]	0.514 [0.360-0.620]	0.128
GGT, U/L	25.0 [17.0-42.0]	22.0 [15.0-33.0]	27.0 [18.0-43.0]	33.0 [21.0-58.0]	53.0 [32.0-99.0]	**<0.001**
GGT xULN	0.500 [0.350-0.800]	0.475 [0.350-0.725]	0.517 [0.367-0.800]	0.633 [0.433-1.07]	0.917 [0.617-1.65]	**<0.001**
Glucose, mg/dl	99.4 (18.1)	99.7 (19.7)	98.9 (16.6)	101 (18.6)	105 (21.3)	**0.017**
HbA1c (%)	5.53 (0.496)	5.59 (0.555)	5.48 (0.436)	5.52 (0.491)	5.57 (0.956)	**<0.001**
Cholesterol, mg/dl	227 (40.9)	226 (41.9)	227 (39.5)	234 (42.6)	233 (51.9)	**0.004**
HDL cholesterol, mg/dl	59.4 (16.8)	58.0 (15.6)	59.7 (16.9)	61.9 (19.3)	64.4 (20.3)	**<0.001**
LDL cholesterol, mg/dl	148 (37.3)	149 (37.5)	147 (36.4)	151 (39.3)	150 (50.7)	0.325
Triglycerides, mg/dl	104 [77.0-145]	104 [77.0-143]	103 [76.0-145]	114 [82.0-158]	117 [85.5-166]	**0.006**
SCORE2, points^1^	4.8 [2.9-7.3]	4.3 [2.6-6.6]	4.9 [3.0-7.4]	5.8 [4.0-9.3]	7.1 [4.9-8.6]	**<0.001**
FRS, points^2^	0.132 [0.0738-0.229]	0.115 [0.0658-0.208]	0.134 [0.0766-0.237]	0.165 [0.0925-0.278]	0.178 [0.122-0.277]	**<0.001**
CRP, mg/dl	0.180 [0.100-0.360]	0.200 [0.100-0.400]	0.160 [0.100-0.300]	0.160 [0.100-0.300]	0.200 [0.100-0.500]	**<0.001**
Monocytes, %	7.20 [6.00-8.70]	6.80 [5.70-8.40]	7.40 [6.20-8.90]	7.60 [6.65-8.70]	7.05 [6.08-8.35]	**<0.001**
Monocytes, G/L	0.410 [0.330-0.510]	0.400 [0.320-0.490]	0.420 [0.330-0.520]	0.410 [0.355-0.545]	0.330 [0.310-0.403]	**0.045**
Monocyte to lymphocyte ratio	0.241 [0.190-0.313]	0.231 [0.190-0.303]	0.247 [0.187-0.319]	0.249 [0.207-0.309]	0.244 [0.184-0.303]	0.207

ALT, alanine aminotransferase; AST, aspartate aminotransferase; CRP, C-reactive protein; FRS, Framingham risk score; GGT, gamma-glutamyltransferase; ULN, upper limit of normal. Bold values indicate statistically significant differences with a *p* value *≤*0.05.

1 Only calculated in 2,397 patients aged 40-69, without diabetes and without established cardiovascular disease.

2 Only calculated in patients without established cardiovascular disease. Group comparisons used Kruskal–Wallis tests for continuous variables and chi-square tests for categorical variables; for normally-distributed variables (uric acid and total cholesterol), one-way ANOVA was used.

### Accelerated cardiometabolic features occur independently of ethanol-induced dysbiosis

Mounting evidence suggests that ethanol intake alters microbiome composition and intestinal barrier function, both of which are associated with the progression of SLD and CVD.[21], [22], [23], [24], [25] Accordingly, 16S rRNA gene amplicon sequencing of fecal samples from the 8-week dietary intervention revealed distinct clustering between ethanol-exposed and control mice (Fig. S4A and B). At the genus level, genera within the family *Tannerellaceae* and genus *Akkermansia*, both reported to exert protective effects in liver disease and atherosclerosis development,^26^^,^^27^ showed the largest decrease in relative abundance in ethanol-consuming mice, while *Muribaculaceae* displayed a markedly increased relative abundance (Fig. S4A). Importantly, these changes in fecal microbiome composition did not appear to affect intestinal barrier integrity, as indicated by comparable mRNA and protein levels of tight junction genes (Fig. S4C and E), as well as mucus barrier genes across the groups (Fig. S4D). Consistently, systemic lipopolysaccharide levels were comparable between HFC diet-fed *Ldlr*^*-/-*^ mice receiving regular and ethanol-containing drinking water (Fig. S4F).

**Fig. 4:**
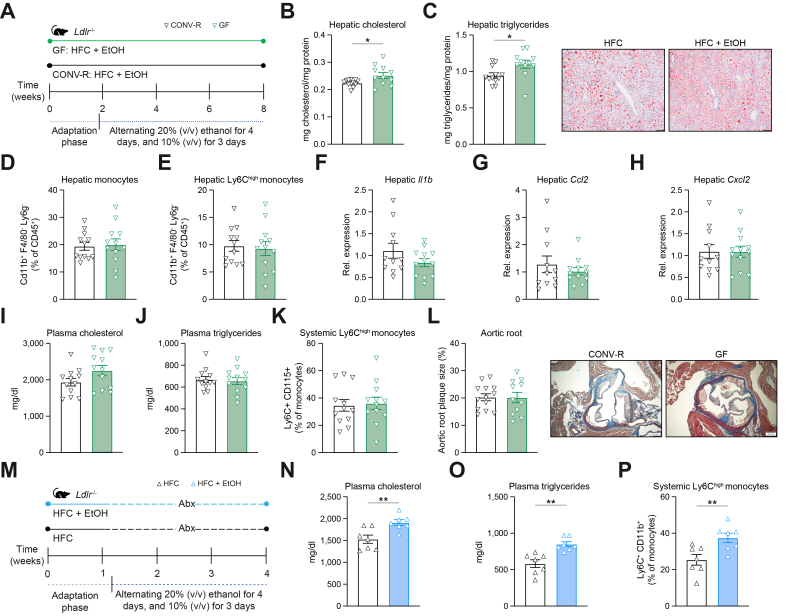
Exacerbated atherosclerosis occurs independent of ethanol-induced dysbiosis. (A-L) 8-week dietary intervention with CONV-R and GF *Ldlr*^*-/-*^ mice. (A) Schematic of the 8-week dietary intervention study in CONV-R and GF *Ldlr*^*-/-*^ mice. (B) Hepatic cholesterol and (C) triglyceride levels and representative images of Oil Red O staining of liver sections. (D) Quantification of hepatic monocytes and (E) Ly6C^high^ assessed by flow cytometry. (F-H) Hepatic gene expression. (I-J) Plasma lipid levels. (K) Quantification of circulating Ly6C^high^ monocytes assessed by flow cytometry. (L) Quantification of aortic root lesion size, expressed as the percentage lesion area relative to total aortic valve area and representative images of Masson’s Trichrome-stained aortic root sections. (M-P) 4-week dietary intervention with antibiotic-induced microbiome depletion. (M) Schematic of the 4-week dietary intervention study in *Ldlr*^*-/-*^ mice, combined with a 3-week antibiotic-induced microbiome depletion. (N-O) Plasma lipid levels. (P) Quantification of circulating Ly6C^high^ monocytes assessed by flow cytometry. Data in (A-L) are mean ± SEM (n = 11–12 for the CONV-R group and n = 11–12 for the GF group). Data in (M–P) are presented as mean ± SEM (n = 7 for the HFC group and n = 7 for the HFC+EtOH group). Statistical significance was determined using a two-tailed unpaired Student’s *t* test; ∗*p ≤*0.05, ∗∗*p ≤*0.01. CONV-R, conventionally raised; EtOH, ethanol; GF, germ-free; HFC, high-fat, high-cholesterol.

To assess the functional contribution of ethanol-induced dysbiosis to steatohepatitis and atherosclerosis, we compared germ-free (GF) and conventionally raised (CONV-R) *Ldlr*^*-/-*^ mice after 8 weeks of HFC diet feeding with ethanol supplementation (Fig. 4A). While body weight and plasma ethanol levels were comparable between GF and CONV-R *Ldlr*^*-/-*^ mice receiving ethanol (Fig. S4G and H), hepatic steatosis was increased in GF mice (Fig. 4B,C). In contrast, hepatic immune cell populations and proinflammatory gene expression profiles did not differ between groups (Figs 4D-H and S4I). Systemic dyslipidemia and circulatory Ly6C^high^ monocytes remained unaffected by the absence of the microbiome in GF *Ldlr*^*-/-*^ mice (Fig. 4I-K). Most notably, quantification of atherosclerotic lesions revealed comparable plaque areas between CONV-R and GF *Ldlr*^*-/-*^ mice receiving HFC diet combined with ethanol-supplemented drinking water (Fig. 4L).

In a complementary approach, *Ldlr*^*-/-*^ mice were fed a HFC diet with either regular or ethanol-containing drinking water for 4 weeks in combination with broad-spectrum antibiotic treatment to deplete the intestinal microbiome (Fig. 4M), a duration sufficient to induce increased systemic dyslipidemia and elevated circulating Ly6C^high^ monocytes upon ethanol intake (Fig. S4J-L). Microbiota ablation was verified by the absence of bacterial growth on agar plates inoculated with cecum contents (Fig. S4M). While ethanol intake in the setting of antibiotic treatment did not affect body weight, plasma ethanol levels were higher in mice receiving ethanol (Fig. S4N and O). Supporting our findings in GF settings, ethanol-fed mice displayed elevated plasma cholesterol and triglyceride levels, as well as increased circulating Ly6C^high^ monocytes despite microbiome depletion (Fig. 4N-P). Collectively, these data indicate that ethanol intake amplifies systemic dyslipidemia, inflammation and aortic root lesion formation in *Ldlr*^*-/-*^ mice on a HFC diet, independently of ethanol-induced gastrointestinal dysbiosis.

### Accelerated atherosclerosis in mice drinking ethanol correlates with exacerbated hyperuricemia and NLRP3 inflammasome activation

**Fig. 5:**
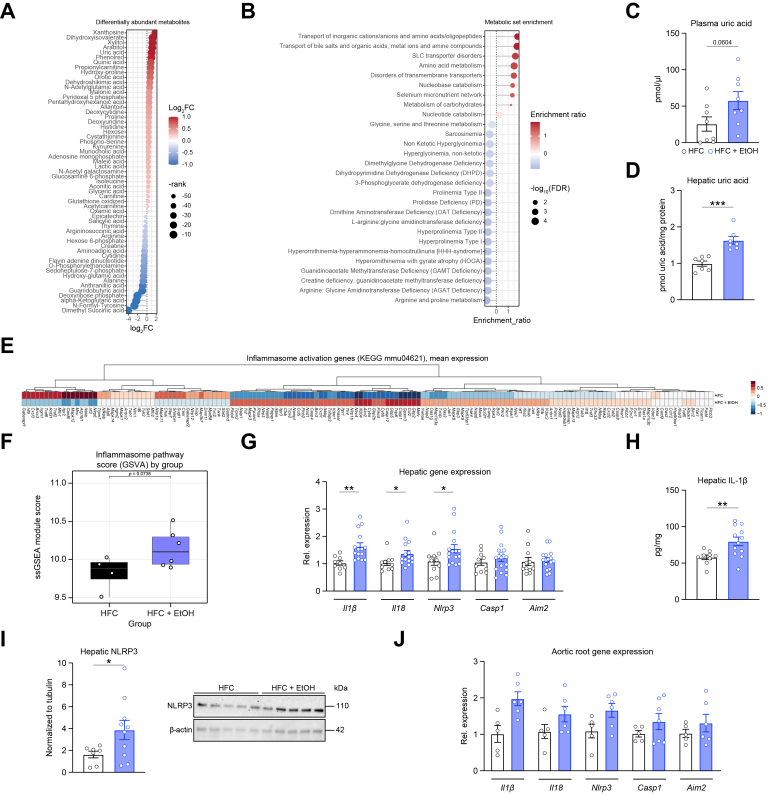
Ethanol intake results in accelerated hyperuricemia and NLRP3 inflammasome activation. (A) Differentially abundant systemic metabolites (n = 8 for the HFC group and n = 9 for the HFC+EtOH group). The color scale indicates the direction of regulation, and dot size is inversely correlated with the rank of a metabolite in the list. (B) Metabolic pathway enrichment based on plasma metabolomics data (n = 8 for the HFC group and n = 8 for the HFC+EtOH group). The color scale indicates the direction of regulation. The enrichment scores are the result of over-representation testing against the MetaboAnalyst pathways universe. (C) Plasma and (D) hepatic uric acid levels. (E) Heatmap showing the expression of genes involved in inflammasome activation (KEGG pathway accession mmu04621) in livers of mice depicted in Fig. 1A. Average values for each group are presented. (F) Inflammasome activation pathway score based on DEG in liver bulk RNA sequencing. (G) Hepatic gene expression. (H) Hepatic IL-1β protein concentration assessment. (I) Hepatic NLRP3 protein levels. (J) Aortic root gene expression. Data are mean ± SEM (n = 5–11 for the HFC group; n = 6–16 for the HFC+EtOH group). mRNA was normalized to *18S* and expressed relative to HFC-fed *Ldlr*^*-/-*^ mice on regular drinking water. Statistical significance was determined using a two-tailed unpaired Student’s *t* tests; ∗∗*p ≤*0.01, ∗∗∗*p ≤*0.001. DEG, differentially expressed genes; EtOH, ethanol; HFC, high-fat, high-cholesterol.

Untargeted plasma metabolite profiling revealed a clear separation between experimental groups based on systemic metabolomic profiles (Fig. S5A), providing insight into ethanol-driven metabolic alterations in the context of SLD. The most upregulated metabolite driving this separation in HFC diet-fed *Ldlr*^*-/-*^ mice receiving ethanol was dihydroxy-isovalerate, together with isoleucine, indicating alterations in branched-chain amino acid metabolism (Figs 5A and S5B). Notably, functional annotation of the differentially regulated metabolites revealed higher levels of uric acid, allantoin, and xanthosine, all key components of purine metabolism, upon ethanol intake (Figs 5A and S5C). Metabolic set enrichment analysis further identified “Transport of inorganic cations/anions and amino acids/oligopeptides,” “SLC transporter disorders,” and “Amino-acid metabolism” among metabolites with higher abundance, whereas those with lower abundance were enriched in “Ornithine aminotransferase deficiency,” “HHH-syndrome,” and related ornithine-arginine pathways (Fig. 5B). These enrichment patterns were concordant with the purine metabolite accumulation. Consistently, uric acid concentrations were elevated in both plasma and liver of mice consuming ethanol (Fig. 5C,D).

These findings align with previous studies documenting an association between hyperuricemia and alcohol consumption in humans.^28^ Moreover, uric acid has been shown to activate the NLRP3 inflammasome, thereby contributing to disease progression.^29^ To assess inflammasome activation in our model, we analyzed transcriptomic signatures for genes involved in inflammasome pathways. Notably, expression of *Nlrp3*, *Casp1*, *Aim2*, *Il1b*, and *Il18* was upregulated in HFC diet-fed *Ldlr*^*-/-*^ mice subjected to ethanol (Figs 5E and S5D), accompanied by an increased inflammasome pathway activity score (Fig. 5F). The inflammasome-related transcriptional changes (Fig. 5G) were corroborated by increased IL-1β concentrations and higher NLRP3 levels in the livers of ethanol- and HFC diet-fed *Ldlr*^*-/-*^ mice (Fig. 5H,I). Importantly, ethanol intake also resulted in increased expression of inflammasome-related genes in the aortic root of HFC diet-fed *Ldlr*^*-/-*^ mice (Fig. 5J). Collectively, these findings support a potential role for uric acid-driven NLRP3 inflammasome activation in promoting atherosclerotic disease progression in mice drinking ethanol.

### Alcohol consumption in humans is associated with elevated cholesterol, uric acid, and monocyte numbers in blood, contributing to higher cardiovascular risk

**Fig. 6:**
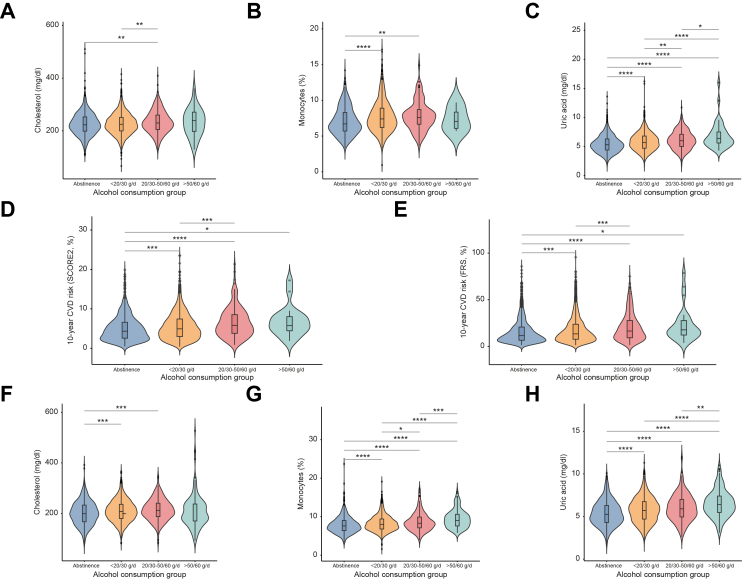
Increased alcohol consumption associates with enhanced systemic dyslipidemia, higher uric acid, elevated monocyte counts and increased cardiovascular risk scores. (A) Systemic cholesterol levels, (B) relative monocyte counts, (C) uric acid levels, (D) SCORE2 risk score, and (E) Framingham risk score in 4,334 individuals of the general population, stratified by alcohol consumption: abstinent, <20/30 g/day, 20/30–50/60 g/day, or >50/60 g/day (Cohort I; excluding statin users). (F) Systemic cholesterol levels, (G) relative monocyte counts, and (H) uric acid levels in 2,166 individuals from a hepatology outpatient clinic, stratified by alcohol consumption: abstinent, <20/30 g/day, 20/30–50/60 g/day, or >50/60 g/day (Cohort II; excluding statin users). Graphs showing violin plots integrated with boxplots of key metabolic parameters in Cohort I (A-E), and Cohort II (F-H). Brackets and asterisks indicate significant differences in post-hoc comparisons applying either Tukey’s (cholesterol, uric acid) or Dunn’s (Monocytes, SCORE2, Framingham risk score) *post hoc* comparisons, as applicable. ∗*p ≤*0.05, ∗∗*p ≤*0.01, *p ≤*0.001 and ∗∗∗∗*p ≤*0.0001.

To interrogate these relationships in a human setting, we analyzed a population-based cohort (Cohort I; n = 5,115; Fig. S6A). After exclusion of statin users, individuals were stratified as abstinent or according to alcohol intake (<20/30 g/day, 20/30–50/60 g/day, and >50/60 g/day). Higher alcohol consumption was associated with progressive increases in systemic levels of gamma-glutamyltransferase, alanine aminotransferase, and aspartate aminotransferase, along with a dose-dependent elevation in cholesterol and triglyceride levels, consistent with our observations in mice (Table 2, Fig. 6A). Lipid-related associations remained robust upon inclusion of statin-treated individuals (Table S2). In line with our murine findings, besides elevated C-reactive protein, alcohol intake resulted in elevated monocyte counts in the blood (Table 2, Fig. 6B). Additionally, uric acid, one of the metabolites identified as elevated in our murine metabolomic profiling and previously reported to be increased in patients with cardiovascular disease, exhibited a dose-dependent increase in circulating levels with increasing alcohol intake^14^^,^^30^^,^^31^ (Table 2, Fig. 6C). Consequently, alcohol consumption was associated with a dose-dependent increase in cardiovascular risk, as assessed by SCORE2 and the Framingham risk score (Table 2, Fig. 6D,E). A subgroup analysis of 1,916 individuals with ultrasound-confirmed hepatic steatosis showed consistent results after stratification by alcohol intake, independent of statin use (Table S3).

These findings were independently recapitulated in patients referred to a hepatology outpatient clinic (Cohort II; n = 2,515; Fig. S6B), in which higher alcohol intake was again associated with increased circulating cholesterol, monocyte counts, and uric acid levels (Table 3, Fig. 6F-H), including in patients with confirmed hepatic steatosis (controlled attenuation parameter ≥248 dB/m) (Table S5). Overall, these findings provide translational evidence that higher alcohol intake not only aggravates liver disease progression but also promotes systemic dyslipidemia, monocytosis, and an increase in uric acid levels, thereby contributing to an increased cardiovascular risk.

**Table 3:** Characteristics of 2,166 individuals referred to a hepatology outpatient clinic (Cohort II) excluding statin users (n = 349, 13.9%), stratified by self-reported alcohol intake.

Parameter	Overall (N = 2,166)	Abstinence (n = 636)	<20/30 g/day (n = 1,115)	20/30-50/60 g/day (n = 243)	>50/60 g/day (n = 172)	*p* values
Age, years	50.8 (15.0)	49.6 (15.8)	49.8 (14.8)	54.7 (14.3)	55.8 (12.0)	**<0.001**
Male sex	1,226 (56.6%)	245 (38.5%)	675 (60.5%)	158 (65.0%)	148 (86.0%)	**<0.001**
BMI, kg/m^2^	27.1 (7.94)	27.3 (5.56)	27.1 (9.83)	26.8 (4.18)	26.6 (4.66)	0.738
BMI ≥30 kg/m^2^	502 (23.2%)	182 (28.6%)	245 (22.0%)	44 (18.1%)	31 (18.0%)	**<0.001**
Diabetes	251 (11.6%)	87 (13.7%)	115 (10.3%)	20 (8.2%)	29 (16.9%)	**0.013**
Uric acid, mg/dl	5.75 (1.52)	5.37 (1.43)	5.78 (1.49)	6.03 (1.55)	6.52 (1.56)	**<0.001**
AST, U/L	31.0 [24.0-44.0]	29.0 [23.0-42.0]	30.0 [23.0-41.0]	32.0 [24.5-46.0]	44.5 [32.0-76.5]	**<0.001**
ALT, U/L	39.0 [25.0-63.0]	37.0 [24.0-64.0]	39.0 [25.0-63.0]	41.0 [27.0-63.5]	44.5 [30.8-63.0]	**<0.001**
GGT, U/L	59.0 [29.0-128]	49.0 [23.0-113]	56.0 [27.0-112]	74.0 [38.0-154]	190 [70.8-444]	**<0.001**
Glucose, mg/dl	98.5 (24.2)	99.1 (27.8)	96.9 (22.0)	100 (22.9)	104 (23.9)	**0.002**
HbA1c (%)	5.43 (0.683)	5.53 (0.844)	5.41 (0.598)	5.37 (0.530)	5.29 (0.688)	**<0.001**
Cholesterol, mg/dl	208 (45.9)	201 (46.4)	210 (42.7)	214 (42.1)	208 (63.1)	**<0.001**
HDL cholesterol, mg/dl	58.4 (19.1)	57.5 (18.4)	58.1 (18.7)	61.3 (19.7)	60.0 (22.5)	**0.034**
LDL cholesterol, mg/dl	133 (40.5)	128 (40.1)	135 (38.1)	137 (39.0)	128 (54.8)	**<0.001**
Triglycerides, mg/dl	111 [78.0-162]	107 [78.0-154]	112 [78.0-168]	116 [79.5-154]	117 [89.0-184]	0.059
CRP, mg/dl	0.170 [0.100-0.390]	0.180 [0.100-0.413]	0.150 [0.100-0.360]	0.150 [0.100-0.400]	0.200 [0.100-0.543]	**0.009**
Monocytes, G/L	0.500 [0.400-0.630]	0.480 [0.388-0.620]	0.480 [0.390-0.610]	0.520 [0.410-0.640]	0.615 [0.490-0.763]	**<0.001**
Monocytes, %	8.0 [6.8-9.4]	7.6 [6.4-9.0]	8.0 [6.8-9.4]	8.3 [7.1-9.9]	9.0 [7.6-10.6]	**<0.001**
Monocyte to lymphocyte ratio	0.273 [0.215-0.360]	0.255 [0.204-0.343]	0.271 [0.214-0.347]	0.292 [0.232-0.385]	0.353 [0.265-0.482]	**<0.001**

ALT, alanine aminotransferase; AST, aspartate aminotransferase; CRP, C-reactive protein; FRS, Framingham risk score; GGT, gamma-glutamyltransferase.

Group comparisons used Kruskal–Wallis tests for continuous variables and chi-square tests for categorical variables; for normally-distributed variables (uric acid and total cholesterol), one-way ANOVA was used. Bold values indicate statistically significant differences with a *p* value ≤0.05.

## Discussion

SLD is the leading cause of chronic liver disease worldwide and its prevalence continues to rise due to changes in lifestyle factors, such as the adoption of a Western diet, sedentary lifestyles, and harmful alcohol consumption.^1^ Given the high global prevalence of SLD and the frequent coexistence of metabolic dysfunction and alcohol consumption, the term MetALD has been introduced.^4^^,^^10^ Although cardiovascular diseases are known to be the leading cause of mortality in patients with SLD, the contribution of alcohol consumption to atherosclerosis remains to be elucidated.^32^^,^^33^

In this study, we describe a preclinical mouse model of SLD in the setting of alcohol consumption and show that combined HFC diet and ethanol intake synergistically promote immune-metabolic disturbances, closely recapitulating early metabolic and transcriptomic characteristics of human SLD. Importantly, our results indicate that ethanol-fed hyperlipidemic mice exhibit accelerated atherosclerosis development, and that the systemic alterations in lipid metabolism, inflammation, and hyperuricemia, which are associated with increased cardiovascular risk, are also observed in humans. Of note, our data suggest that disease aggravation may be driven by hyperuricemia, which potentially activates the NLRP3 inflammasome and occurs independently of microbiome composition.

Due to the recent introduction of the term MetALD, murine models that distinguish MetALD from MASLD and ALD are still lacking. Here, we describe a mouse model that recapitulates key hepatic features of human MetALD while also enabling investigation of its cardiovascular consequences. A retrospective study analyzing 39 murine MASLD models demonstrated that Western-style diets closely mimic the progression of human MASLD from early steatosis and inflammation to metabolic dysfunction-associated steatohepatitis with fibrosis, making these diets suitable for studying MetALD by additionally exposing mice to ethanol.^34^ Despite the common practice of combining high-caloric meals with moderate, regular alcohol consumption, to our knowledge, only one murine MetALD model has been introduced so far.^35^ In contrast to the model of Babuta and colleagues, which employed a 12-week dietary intervention with 58 kcal% fat, 1.0% cholesterol, supplemental sucrose and fructose, and periodic binge ethanol exposure resulting in advanced fibrosis, we used a diet containing 21% fat and 0.21% cholesterol over a period of 8 weeks. Our dietary regimen recapitulates early MASLD characteristics, including hepatic steatosis and onset of inflammation, while Babuta and colleagues’ model mimics progression to accelerated fibrosis.^35^ Furthermore, mice in our study exhibited plasma ethanol levels of around 30 mg/dl, compared with 115 mg/dl reported by Babuta and colleagues.^35^ Although the relatively low plasma ethanol levels in our model indicate simulation of moderate alcohol consumption, their physiological relevance is limited, as measurements were obtained at a single time point at euthanasia. Consequently, they do not capture the time course of ethanol consumption, thereby constraining conclusions regarding systemic ethanol exposure. Moreover, the lack of quantification of alcohol intake in g/kg/day represents an additional limitation of this study. However, the human-like lipoprotein profile of *Ldlr*^*-/-*^ mice, together with their susceptibility to atherosclerosis, enhances the translational relevance of our model for investigating alcohol-induced immunometabolic dysregulation and the resulting cardiovascular risk, as well as for exploring novel therapeutic strategies in a preclinical model.^36^

Although it is well established that both a Western diet and alcohol consumption independently modulate the gastrointestinal microbiome and contribute to non-communicable diseases such as SLD and atherosclerosis, their combined impact on microbial composition and disease progression has not been thoroughly studied.^21^^,^^22^^,^^24^^,^^25^ In our model, ethanol intake resulted in a reduced abundance of gut microbes such as *Akkermansia muciniphila*, a bacterium associated with metabolic health, while *Streptococcus* species, linked to subclinical atherosclerosis, were enriched.^27^^,^^37^ However, depletion of the gut microbiota did not alter disease severity. These findings suggest that specific microbial taxa or their metabolites, rather than global community structure, may drive disease progression and represent potential therapeutic targets.

Interestingly, between-group differences in aortic root lesion area were attenuated at 16 weeks. Although plaque composition was evaluated by quantifying collagen and relative macrophage content, no significant differences were detected, likely due to the limited sample size. Nevertheless, ethanol-supplemented HFC-fed *Ldlr*^*-/-*^ mice showed a modest increase in Mac2-positive and a minor reduction in Sirius Red-positive area within aortic root lesions. Therefore, one may speculate that this pattern reflects a shift toward a more proinflammatory, collagen-poor plaque phenotype associated with reduced stability. Alternatively, prolonged HFC feeding may mask ethanol-specific effects at later disease stages, highlighting the importance of early disease interception to mitigate accelerated cardiovascular risk.

Mechanistically, we observed an ethanol-mediated increase in circulating monocytes and uric acid levels in both mice and humans, accompanied by upregulation of NLRP3 inflammasome-related gene and protein expression in mice, suggesting their involvement in disease progression. Ly6C^high^ monocytes, expressing *Ccr2* and *Cx3cr1*, preferentially infiltrate artery walls and atherosclerotic plaques, where they differentiate into M1 macrophages and form foam cells through modified lipoprotein uptake, thereby amplifying local inflammation and tissue damage.^38^^,^^39^ Previous studies have also shown that hyperuricemia correlates with atherosclerosis progression and systemic dyslipidemia, and that soluble uric acid promotes inflammation and atherogenesis through AMPK-mediated activation of the NLRP3 inflammasome.^30^^,^^31^ Further, uric acid has been shown to regulate hepatic steatosis development through NLRP3 inflammasome-dependent mechanisms.^29^ Importantly, xanthine oxidase inhibitors have shown promise in reducing hyperuricemia and atherosclerosis burden.^40^ Collectively, uric acid may serve as a potential biomarker that, together with established risk markers and cardiovascular risk scores, provides a more comprehensive assessment of cardiovascular risk, while uric acid-lowering therapy could represent a strategy to help prevent or manage atherosclerosis in individuals with MetALD. However, to delineate the functional link between alcohol consumption, monocytosis, hyperuricemia, and NLRP3 inflammasome activation, and their collective contribution to cardiovascular risk, targeted modulation of purine metabolism, inhibition of NLRP3 activation, and suppression of monocyte-driven inflammation will be essential.

The Framingham risk score and SCORE2 are multivariable algorithms estimating 10-year cardiovascular risk based on parameters including sex, age, systolic blood pressure, and lipid profiles, several of which can be modulated by chronic alcohol intake. SCORE2 was specifically developed and extensively validated across European populations and is currently the European Society of Cardiology-recommended tool for cardiovascular risk estimation in primary prevention.^41^^,^^42^ Accordingly, while alcohol-related modulation of individual SCORE2 components constitutes a limitation, it simultaneously underscores the close pathophysiological interconnection between alcohol exposure and established cardiometabolic risk determinants captured by these scores.

In conclusion, we demonstrate that a combination of a HFC diet with ethanol intake alters hepatic lipid metabolism, leading to sustained dyslipidemia, heightened inflammatory processes, and hyperuricemia, which is associated with inflammasome activation. Together, these factors accelerate atherosclerosis development and elevate cardiovascular risk. Our findings underscore alcohol as a long-overlooked lifestyle factor that merits consideration in managing SLD and its cardiovascular complications.

## Abbreviations

ALD, alcohol-associated liver disease; CONV-R, conventionally raised; CVD, cardiovascular disease; GF, germ-free; HFC, high-fat, high-cholesterol; *Ldlr*^*-/-*^*,* low-density lipoprotein receptor knockout (mice); MASLD, metabolic dysfunction-associated steatotic liver disease; MetALD, metabolic dysfunction-associated steatotic liver disease with moderate ethanol consumption; SLD, steatotic liver disease.

## Authors’ contributions

CH designed the study concept and experimental approaches, performed the studies, acquired, and analyzed the data, wrote, and edited the manuscript. GS provided and analyzed the human data, and provided data tables and plots, and critically revised and edited the manuscript. OP analyzed -omics data from murine studies and edited the manuscript. DR, DKH, TD, TB, KM, and KB provided technical assistance and critically revised the manuscript. LG provided technical assistance for atherosclerosis analyses. MPK performed the studies in germ-free mice, acquired and analyzed part of the data in germ-free studies, and edited the manuscript. CR provided access to germ-free mice for our studies, and critically revised and edited the manuscript. PP and JS acquired and analyzed the 16S rRNA gene amplicon sequencing data and revised the manuscript. IB critically revised the manuscript. SG, AV, TR, BS, EA, BW, CD provided access to human data, and critically revised and edited the manuscript. CJB provided part of the mice, and critically revised and edited the manuscript. TH designed the study concept and experimental approaches, performed part of the studies, and acquired part of the data, wrote and edited the manuscript, acquired funding.

## Data availability

Data are available upon reasonable request to the corresponding author.

## Financial support

This research was in part supported by a Zukunftskollegs grant (FWF; ZK81B), a WEAVE grant (FWF; I6853), and a Stand Alone grant (FWF; P36774) to TH. GS, TR and BS are supported by the Clinical Research Group MOTION, Medical University of Vienna, Vienna, Austria – a project funded by the Clinical Research Groups Program of the Ludwig Boltzmann Gesellschaft (Grant Nr: LBG_KFG_22_32) with funds from the Fonds Zukunft Österreich.

## Conflicts of interest

GS received travel support from Amgen. SG received travel support from Roche and Ipsen. TR received grant support from AbbVie, Boehringer-Ingelheim, Gilead, MSD, Philips Healthcare, Pliant Pharmaceuticals, W.L. Gore & Associates, and Siemens, served as a speaker for AbbVie, Gilead, Gore, Intercept, Roche, MSD, and W.L. Gore & Associates, received consulting/advisory board fee from AbbVie, Bayer, Boehringer-Ingelheim, Gilead, Intercept, MSD, and Siemens, and travel support from Boehringer-Ingelheim, Gilead, and Roche. BS received travel support from AbbVie, AstraZeneca, Ipsen, and Gilead, grant support from AstraZeneca and Eisai and speaker’s honoraria from AstraZeneca and Eisai. CD is part of the scientific advisory board of SPAR Österreich AG. All other authors declare no conflict of interest.

Please refer to the accompanying ICMJE disclosure forms for further details.
