# Corrigendum to: ‘Alcohol intake reprograms hepatic immune-metabolic circuits to exacerbate murine atherosclerosis and human cardiovascular risk’ [JHEP Reports (2026) 19;8(9):101932]

**DOI:** 10.1016/j.jhepr.2026.102001

**Published:** 2026-09-03

**Authors:** Constanze Hoebinger, Georg Semmler, Oleksandr Petrenko, Dragana Rajcic, Dorothea Katharina Hoffelner, Tereza Duckova, Taras Baranovskyi, Laura Goederle, My Phung Khuu, Nadja Paeslack, Christoph Reinhardt, Petra Pjevac, Joana Séneca, Kamil Mieczkowski, Katharina Burger, Ina Bergheim, Sophie Gensluckner, Andreas Völkerer, Thomas Reiberger, Bernhard Scheiner, Elmar Aigner, Bernhard Wernly, Christian Datz, Christoph J. Binder, Tim Hendrikx

**Affiliations:** 1 Department of Laboratory Medicine, KILM, Medical University of Vienna, Vienna, Austria; 2 Division of Gastroenterology and Hepatology, Department of Medicine III, Medical University of Vienna, Vienna, Austria; 3 Clinical Research Group MOTION, Medical University of Vienna, Vienna, Austria; 4 FLASH Centre for Liver Research, Department of Gastroenterology and Hepatology, Odense University Hospital, Odense, Denmark; 5 Institute of Clinical Research, Faculty of Health Sciences, University of Southern Denmark, Odense, Denmark; 6 Ukrainian Institute for Systems Biology and Medicine, Kyiv, Ukraine; 7 Center for Thrombosis and Hemostasis (CTH), University Medical Center of the Johannes Gutenberg-University Mainz, Mainz, Germany; 8 Research Center for Immunotherapy (FZI), University Medical Center Mainz, Johannes Gutenberg-University Mainz, Mainz, Germany; 9 Joint Microbiome Facility of the Medical University of Vienna and the University of Vienna, Vienna, Austria; 10 Division of Microbial Ecology, Centre for Microbiology and Environmental Systems Science, University of Vienna, Vienna, Austria; 11 Department of Nutritional Sciences, Molecular Nutritional Science, University of Vienna, Vienna, Austria; 12 First Department of Medicine, Paracelsus Medical University Salzburg, Salzburg, Austria; 13 Department of Internal Medicine, General Hospital Oberndorf, Teaching Hospital of the Paracelsus Medical University Salzburg, Oberndorf, Austria

In the original publication, Nadja Paeslack was inadvertently omitted from the author list. Nadja Paeslack made substantial contributions to the experimental work involving gnotobiotic *Ldlr*−/− mice conducted in the laboratory of Prof. Reinhardt. In recognition of these substantial contributions, Nadja Paeslack should have been included as an author of the original article. The author list has therefore been corrected to include Nadja Paeslack. This correction does not affect the scientific conclusions of the article. The authors apologize for this omission and any inconvenience caused.

