# Supplementary material for: Alcohol intake reprograms hepatic immune-metabolic circuits to exacerbate murine atherosclerosis and human cardiovascular risk
Source: JHEP Rep. 2026 Jun 19;8(9):101932. doi: 10.1016/j.jhepr.2026.101932 (PMC13482647; doi:10.1016/j.jhepr.2026.101932)
Supplement: Supplementary file 1 [file mmc1.pdf]

Alcohol intake reprograms hepatic immune-metabolic circuits to exacerbate murine atherosclerosis and human cardiovascular risk

Constanze Hoebinger, Georg Semmler, Oleksandr Petrenko, Dragana Rajcic, Dorothea Katharina Hoffelner, Tereza Duckova, Taras Baranovskyi, Laura Goederle, My Phung Khuu, Christoph Reinhardt, Petra Pjevac, Joana Séneca, Kamil Mieczkowski, Katharina Burger, Ina Bergheim, Sophie Gensluckner, Andreas Völkerer, Thomas Reiberger, Bernhard Scheiner, Elmar Aigner, Bernhard Wernly, Christian Datz, Christoph J. Binder, Tim Hendrikx

Table of contents

Supplementary materials and methods…………………………………………….……2

Supplementary figures……………………………………………………………...13

Supplementary figure legends……………………………………………………..19

Supplementary tables……………………………………………………………….21

Supplementary references……………………………………………………...….30

Supplementary materials and methods

This study adheres to all relevant ethical regulations. All experimental studies conducted in Vienna were approved by the Animal Ethics Committee of the Medical University of Vienna and the Austrian Federal Ministry of Education, Science, and Research (license no. BMWF 2022-0.574.136). These studies were carried out in accordance with Good Scientific Practice and national and international institutional guidelines. Experimental studies conducted in Mainz were approved by the Landesuntersuchungsamt Rheinland-Pfalz, Koblenz, Germany (license no. G20-1-119) and carried out in accordance with Good Scientific Practice and national and international institutional guidelines.

Human data

Cohort I: 5115 subjects aged 40-69 years undergoing first screening colonoscopy within the prospective SAKKOPI registry between 2007 and 2020 were included (i) if data on alcohol consumption and (ii) laboratory data on lipid levels and uric acid were available (1). Alcohol consumption was evaluated using a structured questionnaire completed by participants before the health check-up. Participants reported the frequency of consuming standard beverage units (0.5 L beer, 0.25 L wine, and 0.02 L liquor/spirits) using predefined categories ("never," "seldom," "once per week," "several times per week," "once per day," "twice per day," or "more than twice per day"). Reported frequencies were converted into estimates of total alcohol intake (grams of ethanol per day), which were subsequently used to classify individuals according to established thresholds for SLD categories.

Cardiovascular risk was assessed by SCORE2 and the Framingham Risk Score (FRS) estimating the 10-year risk of fatal and non-fatal cardiovascular events. Of note, SCORE2 was only calculated in patients aged 40-69, without diabetes and without established cardiovascular disease, while FRS was only calculated in those without established cardiovascular disease.

Cohort II: 2515 first-time referrals to the hepatology outpatient clinic of the University Clinic Salzburg between 2016 and 2024 were included (i) if data on self-reported alcohol consumption and (ii) laboratory data on lipid levels and uric acid were available. Alcohol intake was documented based on patient self-report obtained during routine clinical interviews, and converted into daily ethanol intake for classification according to established SLD thresholds.

For both cohorts, patients with other liver diseases than SLD were excluded. Subjects were stratified into abstainers, low consumption (<20/30g/d), moderate consumption (20-50/30-60g/d) and high consumption (>50/60g/d), respectively. Subgroup analysis excluding statin users (Cohort I: n=781, 15.3%; Cohort II: n=349, 13.9%) were provided.

The study was approved by the local ethics committees (Cohort I: 415-E/1262/2-2010; Cohort II: PMU-EK-2024-0059). All patients from Cohort I provided written informed consent for the analysis of their data.

Patient characteristics were summarized overall and by alcohol groups using descriptive statistics (continuous variables reported as median [IQR] and/or mean [SD], categorical variables as counts and percentages). Group comparisons used Kruskal–Wallis tests for continuous variables and chi-square tests for categorical variables; for normally-distributed variables (uric acid and total cholesterol), one-way ANOVA was used.

Results were visualized for selected variables of interest using violin plots and annotated for significant pairwise differences. Specifically, when the global test was significant ($p \leq 0.05$),

pairwise post-hoc comparisons were performed using Tukey's HSD after ANOVA (family-wise error control) or Dunn's tests with Benjamini–Hochberg adjustment after Kruskal–Wallis. All tests were two-sided with a significance level of 0.05.

Animal models

Ldlr$^{-/-}$ mice were provided by Prof. C. J. Binder, Medical University of Vienna, Vienna, Austria. These mice (B6.129S7-Ldlrtm1Her/J, JAX stock no. 002207) were originally obtained from The Jackson Laboratory and were subsequently bred in-house. All mice were on a C57BL/6J background, and age- and sex-matched littermate controls were used in all experiments. Mice were bred under specific pathogen-free conditions in the Department of Biomedical Research or the Department of Laboratory Animal Science and Genetics of the Medical University of Vienna, Austria.

Age-matched female *Ldlr*$^{-/-}$ mice (9–13 weeks old) were used for all experiments. The mice were bred and maintained in individually ventilated cages (IVCs) under a 12-hour light/dark cycle with ad libitum access to sterilized food and water, under barrier-specific pathogen-free conditions at the Core Facility for Animal Breeding and Husbandry of the Center for Biomedical Research at the Medical University of Vienna. Ambient temperature was maintained at approximately 22 °C (range: 20–24 °C) with a relative humidity of around 55% (range: 45–65%). To induce MASLD and atherosclerosis, mice were fed a high-fat, high-cholesterol diet (HFC) containing 21% milk fat and 0.21% cholesterol (TD88137, ssniff-Spezialdiäten GmbH) with ad libitum access to pellets. To induce MetALD and atherosclerosis, we adapted the protocol described by *Schonfeld et al* (2). Mice received HFC diet with *ad libitum* access to pellets in combination with ethanol in the drinking water. Ethanol was administered as the sole source of drinking water at specified concentrations for treatment durations of 8 or 16 weeks. In the MetALD groups, the concentration of ethanol in the drinking water was progressively increased in a stepwise manner: Mice received 1%, 3%, 10%, 15%, and 20% (v/v) ethanol for 3 days each. Upon reaching 20% (v/v), the regimen continued with an alternating pattern of 20% (v/v) for 4 days followed by 10% (v/v) for 3 days.

For experiments evaluating the role of the intestinal microbiota in liver disease and atherosclerosis development, additional groups were incorporated. In these studies, mice were maintained on the HFC diet for 4 weeks to induce enhanced dyslipidemia and monocytosis, in combination with ethanol in the drinking water. In the MetALD group, mice received 1%, 3%, 10%, 15%, and 20% (v/v) alcohol for 2 days each, and upon reaching 20% (v/v), the regimen continued with alternating 20% (v/v) ethanol for 4 days and 10% (v/v) for 3 days. During the final 3 weeks of the 4-week period, mice received daily oral gavage with an antibiotic cocktail (0.1 mg/g metronidazole, 0.26 mg/g ampicillin, and 0.13 mg/g vancomycin) to deplete the intestinal microbiota. Mice were housed in groups of 2 to 3 per cage, and body weight was assessed weekly. Blood samples and fecal pellets were collected at baseline (prior to antibiotic treatment, week 1), week 4, week 8 and week 16. At the end of the experiment, mice were killed by CO2 overdose under isoflurane anesthesia, and blood was drawn via the vena cava. The number of mice in each experimental group is indicated in the figure legends.

GF and CONV-R *Ldlr*$^{-/-}$ mice shown in Fig. 4 and Fig. S4 were bred at the Translational Animal Research Center of the University Medical Center Mainz. *Ldlr*$^{-/-}$ mice were rederived as GF by aseptic hysterectomy and maintained as a GF mouse colony in sterile flexible film mouse isolator systems. CONV-R *Ldlr*$^{-/-}$ mice were housed under specific-pathogen-free conditions.

All experimental animals were 11-14 weeks old and maintained in EU type II cages under a 12-hour light/dark cycle with ad libitum access to sterilized food and water. Mice received standard autoclaved laboratory diet and water ad libitum, 22 °C ± 2 °C room temperature,a 12-h light-dark cycle, and 55–65% humidity. Induction of MetALD was performed as described above. Mice were housed in groups of 3 to 5 per cage and body weight was assessed twice per week. Blood samples and fecal pellets were collected at week 8. At the end of the experiment, mice were killed by CO2 overdose, and blood was drawn via the vena cava. The number of mice in each experimental group is indicated in the figure legends.

Biochemical analyses in murine models

Blood was collected via tail vein bleeding directly into EDTA collection tubes (Greiner Bio-One, Germany) during the dietary intervention, and postmortem from the vena cava using a 23-gauge needle and transferred into EDTA collection tubes at the experiment's end. All blood collections were performed after a 4-hour fasting period. Plasma was obtained by centrifugation at 2,000 × g for 10 minutes at 8 °C. Plasma and hepatic triglyceride and cholesterol levels were measured according to the manufacturer's instructions using liquid reagents kit (GPO-PAP Triglyceride Liquicolor kit, CHOD-PAP Cholesterol Liquicolor kit, HUMAN Biochemica, and Diagnostica mbH, Wiesbaden, Germany). Protein content was measured using the Pierce BCA Protein Assay Kit (Thermo Fisher Scientific, Waltham, MA, USA). Plasma and hepatic uric acid levels were measured according to the manufacturer's instructions using Uric Acid Assay Kit (ab65344, Abcam, Netherlands). Plasma free fatty acids were quantified using the MAK044-1KT kit (Sigma-Aldrich, Vienna, Austria), and plasma ethanol levels were measured with the Ethanol assay kit (MAK076-1KT, Sigma-Aldrich, Vienna, Austria) following the manufacturer's protocol. Systemic LPS levels were assessed according to the manufacturer's protocol using Mouse Lipopolysaccharides (LPS) ELISA Kit (CSB-E13066m, Hölzel Diagnostika Handels GmbH, Köln, Germany). Hepatic IL-1β protein concentration was determined using the DuoSet™ ELISA Development System Mouse IL-1β/IL-1F2 (DY401), according to the manufacturer's protocol. Plasma immunoglobulins were quantified by sandwich ELISA. IgM levels were measured using anti-mouse IgM for coating (AP500, Sigma-Aldrich, Austria) and alkaline-phosphatase–conjugated anti-mouse IgM for detection (A9688, Sigma-Aldrich, Austria), as previously described (3). For IgG1, plates were coated with anti-mouse IgG1 (clone RMG1-1; 406602, BioLegend, San Diego, USA) and detected with biotinylated anti-mouse IgG1 (clone A85-1; 553441, Becton Dickinson, Austria). Total IgA concentrations were measured using a chemiluminescent sandwich ELISA with anti-mouse IgA for capture (clone C10-3; 556969, Becton Dickinson, Austria) and biotinylated anti-mouse IgA for detection (clone C10-1; 556978, Becton Dickinson, Austria).

RNA Isolation

For whole-liver RNA isolation, 50 mg tissue samples were collected from the left lateral liver lobe and snap-frozen. For aortic RNA isolation, the entire aorta was harvested and snap-frozen. For small intestine RNA isolation, a small portion of the whole intestinal tissue was snap-frozen in liquid nitrogen and used for RNA extraction. The frozen tissues were homogenized in QIAzol lysis reagent (Cat. No. 79306, Qiagen, Hilden, Germany) using 1.0 mm TriplePure M-Bio Grade High Impact Zirconium beads (Lot: 44544432, Benchmark) in a TissueLyser II (Qiagen, Hilden, Germany). RNA extraction was performed according to the manufacturer's

instructions, and RNA quantity and quality were subsequently assessed using a NanoDrop spectrophotometer (Peqlab).

cDNA Synthesis and Quantitative Real-Time PCR (qPCR)
Up to 1 µg of total RNA was reverse transcribed into cDNA using the High-Capacity cDNA Reverse Transcription Kit (Applied Biosystems, Thermo Fisher, Waltham, MA, USA) following the manufacturer's protocol. qPCR was carried out on a CFX96 Real-Time PCR System (Bio-Rad Laboratories, Hercules, CA, USA) using the KAPA SYBR FAST kit (Thermo Fisher, Waltham, MA, USA) with the primers listed below. Relative gene expression was calculated by the $2^{(-\Delta\Delta Ct)}$ method, with normalization to the housekeeping gene 18S. Primer sequences are provided in Table 4.

Primer details

Target Gene	Forward Sequence (5'-3')	Reverse Sequence (5'-3')
Abca1	GAGCAAAGCCAAGCATCTTC	AGCAGGGACCACATAATTGC
Abcg4	CTGAGCTCTTCCAACACTTCG	GCCACAGAACACCAACTCTC
Abcg8	CTCACCTACCAGGTGGACAT	GCATCTGTCCACTCCTCACT
Adh1	GGGTTCTCAACTGGCTATGG	ACAGACAGACCGACACCTCC
Aim2	AGGCTGCTACAGAAGTCTGTCC	TCAGCACCGTGACAACAAGTGG
Casp1	GGCACATTTCCAGGACTGACTG	GCAAGACGTGTACGAGTGGTTG
Ccl2	AGGTCCCTGTCATGCTTCTG	TCTGGACCCATTCCTTCTTG
Cd36	GCCAAGCTATTGCGACATGA	AAAAGAATCTCAATGTCCGAGACTTT
Cldn1	ATGCCAATTACCATCAAGGC	GAGGGACTGTGGATGTCCTG
Cldn2	TTCTTGGATCCGAGCCTCTA	TCCAGAGCTCTTCGAAAGGA
Cxcl1	GCTGGGATTCACCTCAAGAA	TCTCCGTTACTTGGGGACAC
Cxcl2	AGTGAACTGCGCTGTCAATG	TTCAGGGTCAAGGCAAACTT
Cyp2e1	CTTAGGGAAAACCTCCGCAC	GGGACATTCCTGTGTTCCAG
Cyp7a1	CACCATTCCTGCAACCTTCTGG	ATGGCATTCCCTCCAGAGCTGA
Fatp4	GACTTCTCCAGCCGTTTCCACA	CAAAGGACAGGATGCGGCTATTG
Fut2	GGTGGATGATGGTGGAAGTC	TTCCCTGTTACCACAGCCAG
Il1b	TGTGCAAGTGTCTGAAGCAGC	TGGAAGCAGCCCTTCATCTT
Il18	GACAGCCTGTGTTCGAGGATATG	TGTTCTTACAGGAGAGGGTAGAC
Lpl	GGGAGTTTGGCTCCAGAGTTT	TGTGTCTTCAGGGGTCCTTAG
Muc2	AAGTTTGCCCCTGGCTATGAC	CCTTGCAGTCAAACTCAAAGT
Nlrp3	TCACAACTCGCCCAAGGAGGAA	AAGAGACCACGGCAGAAGCTAG
Npc1l1	ATCGCACTACCATCCAGGACCT	CCCAGAGTAGCCTTGGAATCCA
Ocln	ATTTATGATGAACAGCCCCC	CATAGTCAGATGGGGGTGGA
Reg3b	GGCTTCATTCTTGTCCTCCA	TCCACCTCCATTGGGTTCT
Reg3g	AAGCTTCCTTCCTGTCCTCC	TCCACCTCTGTTGGGTTCAT
S100a8	AAATCACCATGCCCTCTACAAG	CCCACTTTTATCACCATCGCAA
Srebf1	CGACTACATCCGCTTCTTGCAG	CCTCCATAGACACATCTGTGCC
Srebf2	AGAAAGAGCGGTGGAGTCCTTG	GAACTGCTGGAGAATGGTGAGG
Trem2	CTACCAGTGTCAGAGTCTCCGA	CCTCGAAACTCGATGACTCCTC

Zo-1	AGCTGTTTCCTCCATTGCTG	GAGATGTTTATGCGGACGG

Flow Cytometry

For flow cytometric analysis, livers were harvested, flushed *in situ* with PBS to reduce blood-derived contamination, mechanically dissociated, and enzymatically digested for 30 minutes at 37 °C in PBS supplemented with 450 U/ml collagenase I (LifeTech Austria, Vienna, Austria), 125 U/ml collagenase XI (Sigma-Aldrich, Vienna, Austria), 60 U/ml DNase I (LifeTech Austria, Vienna, Austria), and 60 U/ml hyaluronidase (Sigma-Aldrich, Vienna, Austria). The digested tissue was passed through a 40µm cell strainer to obtain a single-cell suspension, and residual erythrocytes were removed using red blood cell lysis buffer (Morphisto, Offenbach am Main, Germany). Blood was collected as described above and also processed with red blood cell lysis (Morphisto, Offenbach am Main, Germany) before the staining procedure. Cells were first incubated with an anti-mouse CD16/32 blocking antibody (clone 93, 0.5 µg per 0.5×10^6 cells; eBioscience) for 20 minutes at 4 °C. This was followed by staining for 30 minutes with a mixture of the following antibodies: BV605-labeled anti-Ly6C (clone HK1.4, 1:1000, BioLegend), PerCP-Cy5.5-labeled anti-CD45 (clone 30-F11, 1:800, eBioscience), FITC-labeled anti-CD11b (clone M1/70, 1:400, BioLegend), PE-labeled anti-CD3e (clone 145-2C11, 1:400, eBioscience), PE-Cy7-labeled anti-Ly6G (clone 1A8, 1:400, BD Biosciences), APC-eFluor780-labeled anti-CD11c (clone N418, 1:800, eBioscience), Alexa Fluor700-labeled anti-B220/CD45R (clone RA3-6B2, 1:800, BioLegend), APC-labeled anti-F4/80 (clone BM8, 1:800, BioLegend), PerCP-Cy5.5-labeled anti-CD115 (CSF-1R) (clone AFS98, 1:400, BioLegend), FITC-labeled anti-CD45 (clone 30-F11, 1:400, BioLegend), and APC-Vio770-labeled anti-B220/CD45R (clone REA755, 1:800, Miltenyi Biotec). All staining procedures were performed in 100 µl of FACS buffer (PBS supplemented with 2% FBS) for 30 minutes at 4 °C in the dark, followed by two washing steps. Stained cell populations were recorded using a BD FACS Fortessa multiparameter flow cytometer. During analysis using FlowJo version 10 (Tree Star Inc., Ashland, OR, USA), dead cells and doublets were excluded based on forward- and side-scatter. Gating strategies to identify hepatic immune cell populations by flow cytometry: Kupffer cells (CD45^{+} Ly6G^{-}CD11b^{+}F4/80^{+}), monocytes (CD45^{+}CD11b^{+}F4/80^{-}Ly6G^{-}), Ly6C^{high} monocytes (CD45^{+}CD11b^{+}Ly6G^{-}F4/80$^{-/low}$Ly6C^{high}), neutrophils (CD45^{+}Ly6G^{+}CD11b^{+}F4/80^{-}), T cells (CD45^{+}B220^{-}CD3^{+}), and B cells (CD45^{+}B220^{+}CD3^{-}). Gating strategies to identify systemic immune cell populations by flow cytometry: monocytes (CD115^{+}CD11b^{+}Ly6G^{-}), Ly6C^{high} monocytes (CD45^{+}CD115^{+}CD11b^{+}Ly6G^{-}Ly6C^{high}), neutrophils (CD11b^{+}Ly6G^{-}), T cells (B220^{-}CD3^{+}, and B cells (B220^{+}CD3^{-}).

Western blot

Liver

Western blotting was done as previously described (4). Briefly, liver protein samples were prepared in RIPA buffer (50 nM Tris-HCl, pH 8, 0.5% SDS, 150 mM NaCl, 1% Triton X-100, 0.5% sodium deoxycholate) supplemented with protease inhibitor cocktail tablets (Roche) and phosphatase inhibitor cocktail 2 (Sigma-Aldrich). Protein concentration was measured using the Pierce BCA Protein Assay Kit (Thermo Fisher Scientific, Waltham, MA, USA). 50 µg of protein per lane were loaded, resolved in SDS-PAGE, and transferred onto a nitrocellulose

membrane. The membranes were blocked in 5% skimmed milk in PBS-T and immunoblotted overnight with the following primary antibodies at 4 °C: SREBP1 (Abcam, clone 2A4, dilution 5 µg/mL), SREBP2 (Abcam, dilution 1:500), TUBULIN (Abcam, dilution 1 µg/mL), NLRP3 (eubio, dilution 1 µg/mL), and ß-ACTIN (Abcam, 1 µg/mL). Next, after washing steps in PBS-T, secondary antibodies ECL™ donkey anti-rabbit IgG-HRP (Amersham) or ECL™ sheep anti-mouse IgG-HRP (Amersham) were used. Chemiluminescence detection was performed with Clarity™ Western ECL Substrate (Bio-Rad) and acquired with a ChemiDoc Imager (Bio-Rad). Densitometry quantification of bands representing detected proteins was done with ImageJ software. Densitometry data are presented as mean cleaved-to-precursor protein ratios adjusted to the loading control Tubulin. Densitometry quantification was performed on all available samples.

Intestine

RIPA protein lysates of small intestinal tissue (20 µg/lane for OCCLUDIN; 10 µg/lane for ß-ACTIN) were separated on 10% SDS-polyacrylamide gels and transferred to polyvinylidene difluoride membranes (Bio-Rad Laboratories) as detailed before (5). Membranes were further incubated with specific primary antibodies (Occludin, Invitrogen; ß-Actin, Santa Cruz) and the respective secondary antibody (anti-mouse IgG, HRP-linked; Cell Signaling Technology, Massachusetts, USA). To detect the protein bands, the Super Signal Western Dura kit (Thermo Fisher Scientific) was used, and densitometric analysis were performed using ChemiDoc XRS System (Bio-Rad Laboratories) as detailed previously (6).

Histology and immunohistochemistry

Liver

The left lobes of the livers were harvested and divided into four equal pieces, each snap-frozen in liquid nitrogen for subsequent analyses. Frozen liver sections (7 µm) were prepared and stained for infiltrating neutrophils as previously described by us (7). Neutrophils were detected using a mouse anti-Ly6G antibody (1:100; BD PharMingen), and cell nuclei were counterstained with hematoxylin (Klinipath). The number of positively stained cells was counted in 5 pictures per liver per mouse to determine the level of liver inflammation. To assess lipid content, additional frozen liver sections were stained with Oil Red O (Sigma-Aldrich, Vienna, Austria) and quantified as previously described (8). Pictures were taken with a Zeiss AxioImager A1 microscope equipped with an AxioCam MRC5 camera and ZEN 2.3 Pro software (Carl Zeiss). Formalin-fixed liver samples (Tissues were collected and stored in 3.7% formaldehyde for 16-24h, and then transferred to PBS and kept at 4°C) were embedded in paraffin, and 4 µm sections were stained with Mayer's Hematoxylin solution (254766, AppliChem, Darmstadt, Germany) and Eosin Y solution (HT110321, Sigma-Aldrich, Vienna, Austria) for assessing general tissue morphology. Collagen deposition was evaluated by Sirius Red staining of liver sections as previously described (9). F4/80 immunohistochemical staining was performed on paraffin-embedded liver sections. Slides were deparaffinized and rehydrated, followed by heat-induced antigen retrieval in citrate buffer (pH 6.0; Sigma-Aldrich) at 98-99 °C. Sections were blocked with 10% normal donkey serum (Sigma-Aldrich) in PBS containing 0.05% Tween-20, followed by avidin/biotin blocking (Abcam) according to the manufacturer's instructions. Sections were then incubated overnight at 4 °C with a rabbit anti-

mouse F4/80 primary antibody (1:500; Cell Signaling Technology, clone D2S9R) diluted in PBS containing 4% fetal bovine serum. Endogenous peroxidase activity was quenched using 3% H_2O_2 in PBS/Tween. Slides were subsequently incubated with a biotinylated goat anti-rabbit IgG secondary antibody, followed by streptavidin-horseradish peroxidase (1:1000; Sigma-Aldrich). Signal detection was performed using a DAB substrate kit (DAKO). Sections were counterstained with hematoxylin, mounted using Aquamount (Merck), and imaged using a Zeiss AxioImager A1 microscope equipped with an AxioCam MRC5 camera and ZEN 2.3 Pro software (Carl Zeiss). Images were analyzed for F4/80$^+$ macrophages using ImageJ (v1.53), and the percentage of positive staining per liver section was quantified. At least five randomly selected fields per liver section were analyzed for each mouse.

Aorta, heart, and innominate artery preparation:
Immediately post-mortem, mice underwent perfusion via the left ventricle first with 20 mL of PBS, (followed by perfusion with 3.7% formaldehyde (Merck Millipore) for mice undergoing 8 weeks dietary regimen). Perivascular tissue was removed in situ, and the hearts, brachiocephalic (innominate) arteries, and aortas (extending to the iliac bifurcation) were isolated and stored in 3.7% formaldehyde for 16–24 hours. Tissues were then transferred to PBS until further processing. Ventricular portions were excised along with the atria to expose the aortic root for subsequent cross-sectional analyses.

Aortic root cross-section and lesion quantification
8-week dietary intervention study: Hearts were prepared and analyzed as previously described by us (10). 16-week dietary intervention study: Hearts, including the aortic root, were embedded in OCT compound, and aortic root sections were prepared using a cryostat (Leica CM1950) at a thickness of 10 µm, beginning at the anatomical level where all three aortic valve leaflets became visible. Sections were sequentially collected throughout the entire aortic root region. For cross-sectional analysis, sections were stained with Mayer's Hematoxylin solution (254766, AppliChem, Darmstadt, Germany) and Eosin Y solution (HT110321, Sigma-Aldrich, Vienna, Austria). Images were photographed on a Zeiss AxioImager A1 using AxioCam MRC5 and ZEN 2.3 Pro software (Carl Zeiss). Lesion size in all three leaflets was assessed by computer-assisted image analysis using Photoshop Elements and ImageJ software. For cross-sectional lesion analysis, data are presented as the average total lesion size across eight sections collected at 80 µm intervals, spanning 560 µm of the aortic root, with total lesion size defined as the sum of lesions at the three leaflets at each location.

8-week dietary intervention study in GF and CONV-R mice: Hearts, including the aortic root, were fixated in 4% PFA for 24 h. Paraffin embedding was performed by the Histology Core Facility (University Medical Center Mainz, Germany). Serial cross-sections of the aortic root were collected at a thickness of 5 µm using a rotatory microtome (Leica Biosystems) and stained with Trichrome Stain Kit (ab150686, Abcam, Cambridge, UK) for atherosclerotic lesion size. Images were acquired on an Olympus BX51 microscope (Olympus). Lesion size in all three leaflets was assessed by computer-assisted image analysis using CellSens Dimension (v.4.1) (Carl Zeiss). For cross-sectional analysis, lesion size was expressed as the average total lesion area across eight sections, collected at 50 µm intervals over a span of 400 µm over the

aortic origin, with total lesion size defined as the sum of lesions at the three leaflets at each location.

Aorta Sudan IV staining and lesion quantification

For *en face* preparations, aortas were pinned onto standard wax dissection pans submerged in PBS, and remaining adventitial tissue was removed. The aortas were washed in Sørensen's phosphate buffer (pH 7.38) and subsequently incubated for 15 min in Sudan IV Staining Solution (0.5% Sudan IV in 1:1 acetone and 70% ethanol, filtered; Sigma-Aldrich) with periodic shaking. Aortas were subsequently incubated in 80% ethanol for 5 min, followed by washing in water. Images were acquired on a Zeiss Stemi Dissecting Microscope with Axiocam 208 Color and ZEN Blue software (Carl Zeiss). Lesion size was assessed in a double-blinded fashion by computer-assisted analysis using Photoshop Elements (Adobe) and ImageJ. Data are expressed as the percent of Sudan IV^+ area per total aortic area.

Immunohistochemistry of the heart

Lesional macrophage content was assessed by immunostaining of paraffin-embedded sections obtained after 8-week dietary intervention using antibodies against MAC2 (BioLegend, #125401, clone M3/38; dilution 1:1,500) and analyzed as previously described (10). Cryosections from mice subjected to 16-week dietary intervention were stained according to the same protocol using adjusted antibody dilutions (1:300). For cryosections, dehydration and antigen retrieval were omitted. Instead, sections were air-dried for 45 min, incubated in ice-cold acetone for 10 min, followed by an additional 15 min air-drying step prior to initiation of the staining procedure.

Sirius Red staining of the heart

For quantification of collagen deposition, paraffin-embedded sections and cryosections of aortic root lesions were stained with Sirius Red F3B (CI 35782; Sigma-Aldrich). Paraffin- and cryosections were prepared as described above. After washing in PBS and rinsing in distilled water, sections were treated with 0.2% phosphomolybdic acid and stained with 0.1% Sirius Red in picric acid. Sections were subsequently washed in 0.01 N HCl, dehydrated, cleared, and mounted with Entellan. Collagen content within aortic root lesions was quantified using Photoshop Elements and ImageJ software.

Transcriptomic data acquisition and analysis

Data acquisition

Sequencing libraries from total RNA of the samples were prepared at the Core Facility Genomics, Medical University of Vienna, using the NEBNext Poly(A) mRNA Magnetic Isolation Module and the NEBNext UltraExpress RNA Library Prep Kit for Illumina with Unique Dual Indices (UDIs) according to manufacturer's protocols (New England Biolabs). Libraries were QC-checked on a Bioanalyzer 2100 (Agilent) using a High Sensitivity DNA Kit for correct insert size and quantified using Qubit dsDNA HS Assay (Invitrogen). Libraries were sequenced on a P3 flowcell of a NextSeq2000 instrument in 1x50bp single-end sequencing mode. On average, 34 million reads per sample were generated. Reads in fastq format were

generated using the Illumina bcl2fastq command line tool (v2.19.1.403) incl. trimming of the sequencing adapters.

Data analysis

This analysis has been completed in R (v.4.3.3). Protein-coding genes were identified with biomaRt and utilized in the downstream analysis (11). The exploratory principal component analysis has been performed using PCAtools with the removal of 10% of genes with the lowest variance. Outlier identification has been performed based on the intersections of the first two principal components, after which the outliers (n = 2) were removed.

DESeq2 (v.1.40.2) with the design formula ~group was used for differential expression analysis using pairwise Wald testing (12). The shrinkage of log_2(Fold Change) inflation has been performed using ashr (v.2.2-63) (13). Multiple testing correction has been performed with independent hypothesis weighting (IHW, v.1.28.0) (14). The thresholds for differentially expressed genes (DEGs) were set as follows: shrunk log2(Fold Change) > |0.27| (equalizing to >20% expression difference between the groups), $P_{adjusted} < 0.05$. The DEGs visualization has been made with EnhancedVolcano (v.1.20.0)

Functional analysis of DEGs has been performed using clusterProfiler (v.4.8.1) (15) in gene set enrichment analysis mode against Reactome, Biocarta, Immunosig and Wikipathways databases. For this analysis, genes were ranked using the $sign(log_2FoldChange) \times -log_{10}(Pvalue)$ formula. For ranking genes of the principal components and network members, gProfiler2 (v.0.2.3) (16) with the over-representation testing were used against Gene Ontology (MF), KEGG, Reactome, and WikiPathways databases. The Z-normalized expression differences of all genes of the selected significant pathways from the over-representation testing were then presented as heatmaps.

Inflammasome activation analysis

A curated list of 192 inflammasome activation-related genes was derived from the KEGG mmu04621 pathway. For variance analysis genes without variance across samples or those without detected transcripts in all samples were removed. For variance analysis and visualization, DESeq2-normalized (v.1.40.2) (12), and scaled counts of the remaining genes were used, and displayed as per-sample variance using pheatmap (v.1.10.12).

For the whole pathway-level analysis, we employed gene set variance analysis (GSVA, v.1.50.5) (17). In brief, we used full counts matrix normalized with DESeq2's Variance stabilizing transformation as input for GSVA with Gaussian kernel density estimation, receiving pathway activity score per-sample. The normality of the score distribution was assessed using the Shapiro-Wilk test independently for each experimental group. Homogeneity of variance between group was tested using Levene's test (median-centered), confirming the assumption of equal variances ($p = 0.57$). The normality assumptions were satisfied as well ($p > 0.05$ for both groups), hence a Welch's two-sample t-test was conducted to determine difference in the pathway activation.

Data Availability Statement

The data for this study have been deposited in the European Nucleotide Archive (ENA) at EMBL-EBI under accession number PRJEB106963.

Metabolomics

Plasma extraction

Twenty microliters of plasma sample were spiked with a mixture of heavy isotope labeled internal standards. Samples were extracted using methanol and ultrasonic assisted extraction (on ice). Samples were centrifugated and the supernatants were evaporated using a nitrogen flow. Dry samples were reconstituted with ultrapure water before the HPLC-MS analysis.

Hydrophilic metabolites acquisition

A 1290 Infinity II UHPLC system (Agilent Technologies) coupled with a 6470 triple quadrupole mass spectrometer (Agilent Technologies) was used for the LC-MS/MS analysis. Chromatographic separation was performed on a ZORBAX RRHD Extend-C18, 2.1 x 150 mm, 1.8 µm analytical column (Agilent Technologies) in ion pairing mode, maintained at 40°C. A 4 µL sample was injected per run. The mobile phase A consisted of 3% methanol (v/v), 10 mM tributylamine, and 15 mM acetic acid in water, while mobile phase B contained 10 mM tributylamine and 15 mM acetic acid in methanol. A gradient elution at a flow rate of 250 µL/min was conducted for 24 minutes. Following this, back-flushing of the column was done using a 6 port/2-position divert valve with acetonitrile for 8 minutes, followed by 8 minutes of column equilibration with 100% mobile phase A. The triple quadrupole mass spectrometer was operated in negative electrospray ionization mode, with a spray voltage of 2 kV, gas temperature of 150°C, gas flow of 1.3 L/min, nebulizer pressure of 45 psi, sheath gas temperature of 325°C, and sheath gas flow of 12 L/min. Metabolites were detected using dynamic MRM mode. Ten-point calibration curves with internal standardization were used for absolute quantification. Data processing was performed using PeakBot software (version 0.9.54) (18).

Metabolomic data analysis

Metabolites with missing values at threshold ≥ 20% were excluded from the analysis (n = 62, 30.8%). The remaining 139 metabolites were processed with POMA (19). The missing values were imputed with kNN and relative abundances were normalized using the log-Pareto method (20). The exploratory principal component analysis was performed using PCAtools, which removed 10% of the genes with the lowest variance. Differential abundance and over-representation analysis were performed with MetaboAnalystR (21). Briefly, compound names were mapped against metabolic databases, and their differential abundance was quantified using the MetaboAnalyst FoldChange module. The metabolites reaching >|0.27| log2FoldChange threshold (i.e., >20% abundance difference) were considered as significant. For the enrichment analysis, MetaboAnalystR implementation of the hypergeometric test was applied, and normalized enrichment scores were used to identify up-/downregulated pathways.

16S rRNA amplicon sequencing of the fecal microbiome

DNA was extracted from fecal/cecum samples using the QIAamp Fast DNA Stool Mini Kit (Qiagen), and 16S rRNA gene amplification, barcoding and sequencing of the hypervariable

V4 region of bacterial and archaeal 16S rRNA genes was performed at the Joint Microbiome Facility (JMF) of the Medical University of Vienna and the University of Vienna under Project ID JMF-2307-04 as previously described by us (22). Amplicon libraries were sequenced on an Illumina Miseq (v3 chemistry), and amplicon pools were extracted from raw sequencing data using the FASTQ workflow in BaseSpace (Illumina) with default parameters. Data was filtered for PhiX contamination with BBDuk (BBTools, Bushnell B), demultiplexing was done using the python package demultiplex (Laros JFJ) allowing one mismatch to barcodes, and two mismatches for linker sequences and primers. Barcodes, linkers, and primers were trimmed off using BBDuk, and forward and reverse FASTQ reads were trimmed at 220 and 150 nt, respectively, with allowed expected errors of 2, followed by ASV inference using the recommended DADA2 workflow in R (23, 24). Taxonomic classification against the SILVA (25) SSU Ref NR 99 rRNA database v138.1 was performed using the classifier implemented in DADA2 using a confidence threshold of 0.5. All downstream analyses were performed in R version 4.0.2.

Data Availability Statement

The 16S rRNA gene amplicon sequencing datasets supporting the conclusions of this article are available in the NCBI repository under BioProject accession number PRJNA1261036.

Statistical analysis

Statistical analyses were performed using GraphPad Prism v10. Data comparing the groups were analyzed by two-tailed unpaired Student’s t-tests following evaluation of Gaussian distribution. Results are expressed as mean ± standard error of mean (SEM) unless stated otherwise.

Additional details are provided in the Supplementary CTAT Table.

Supplementary figures

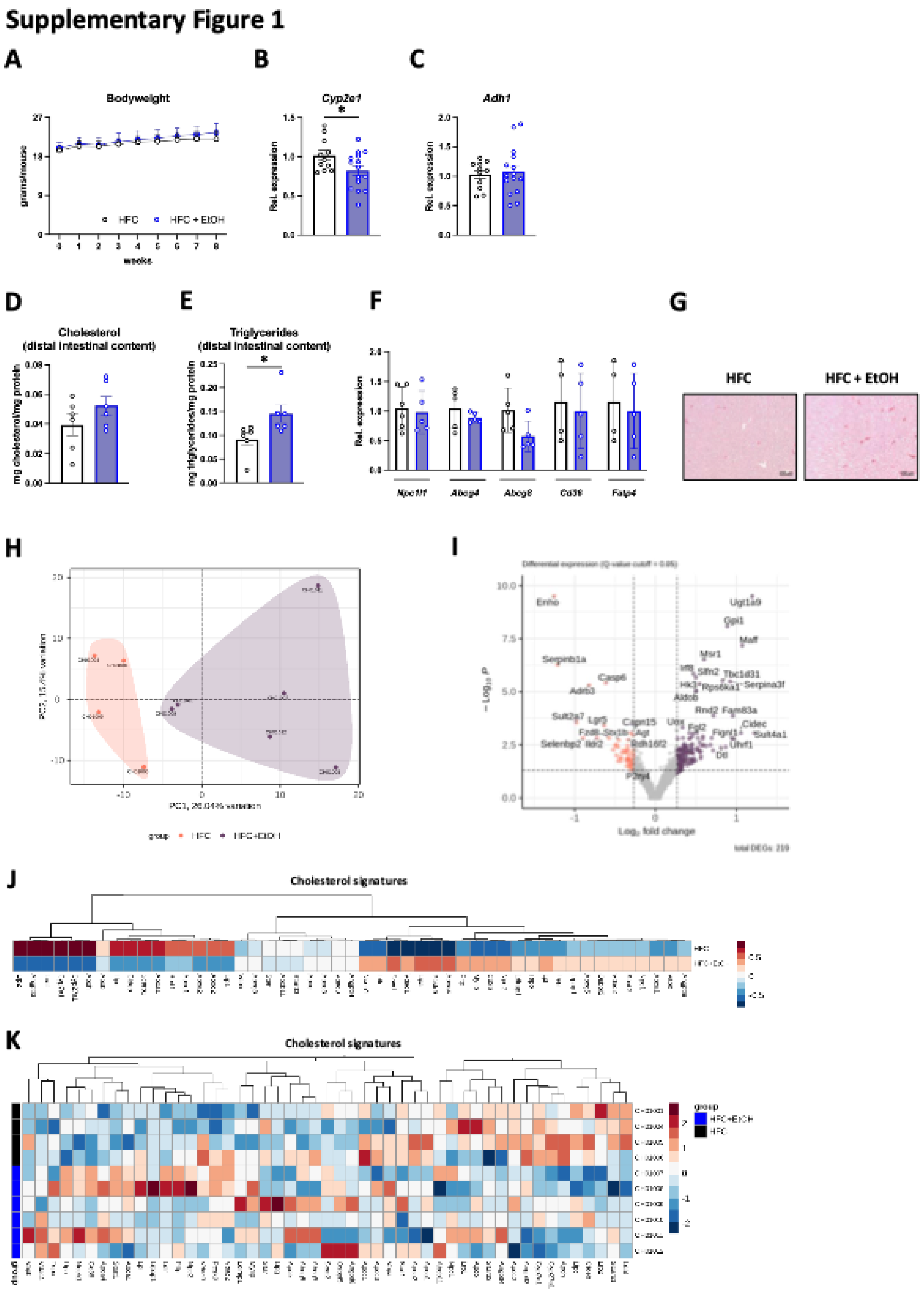

Supplementary Figure 2

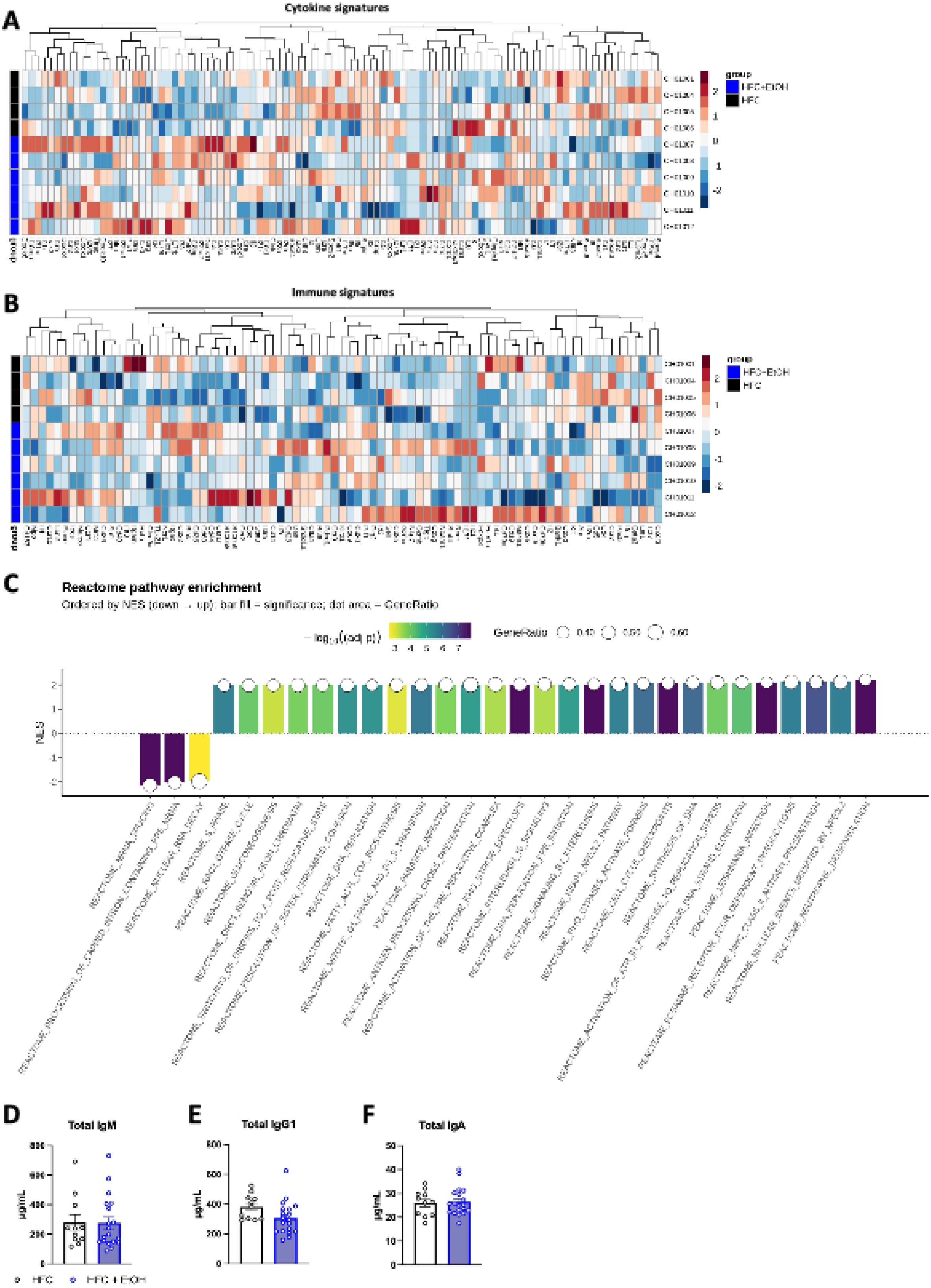

Supplementary Figure 3

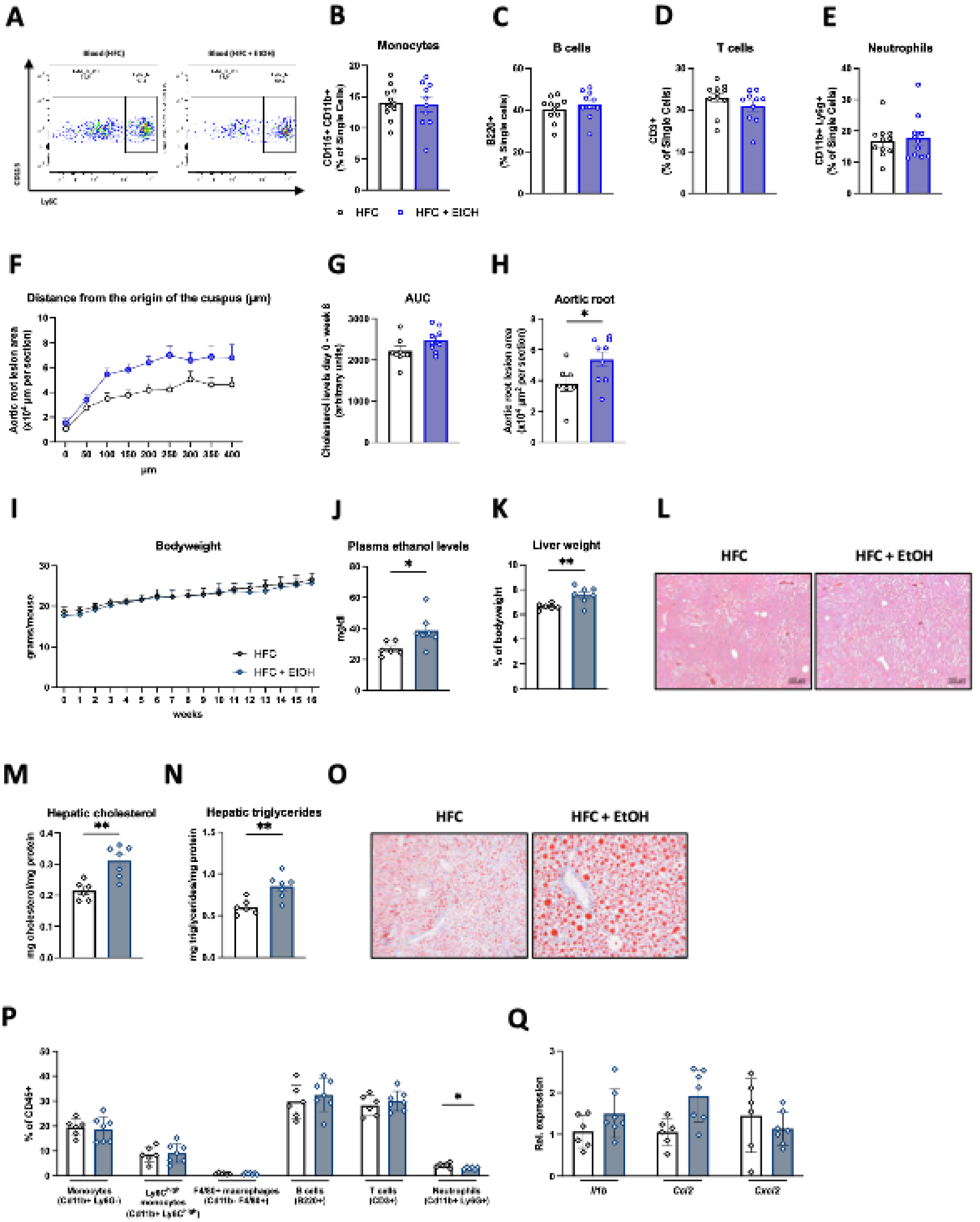

Supplementary Figure 4

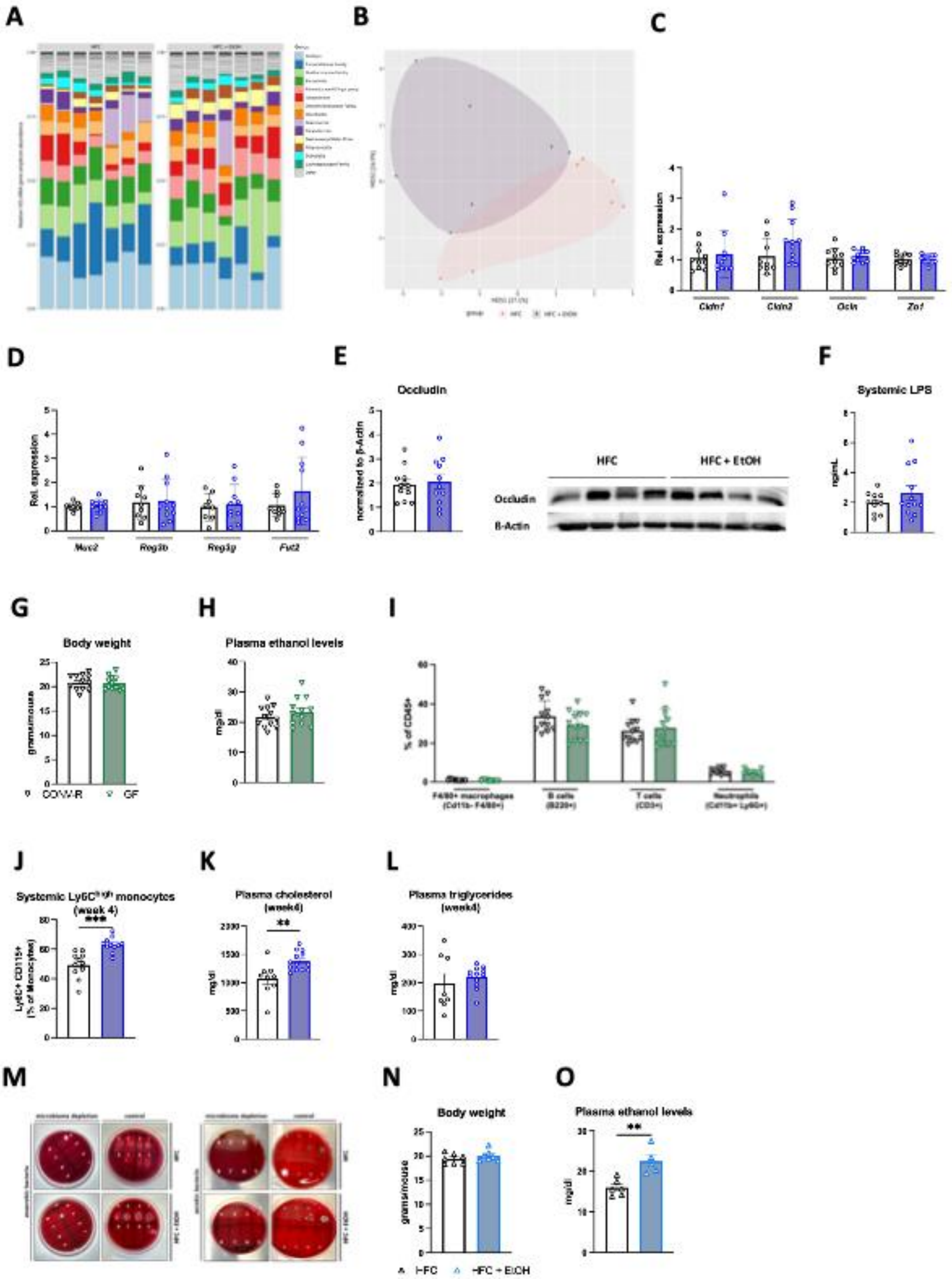

Supplementary Figure 5

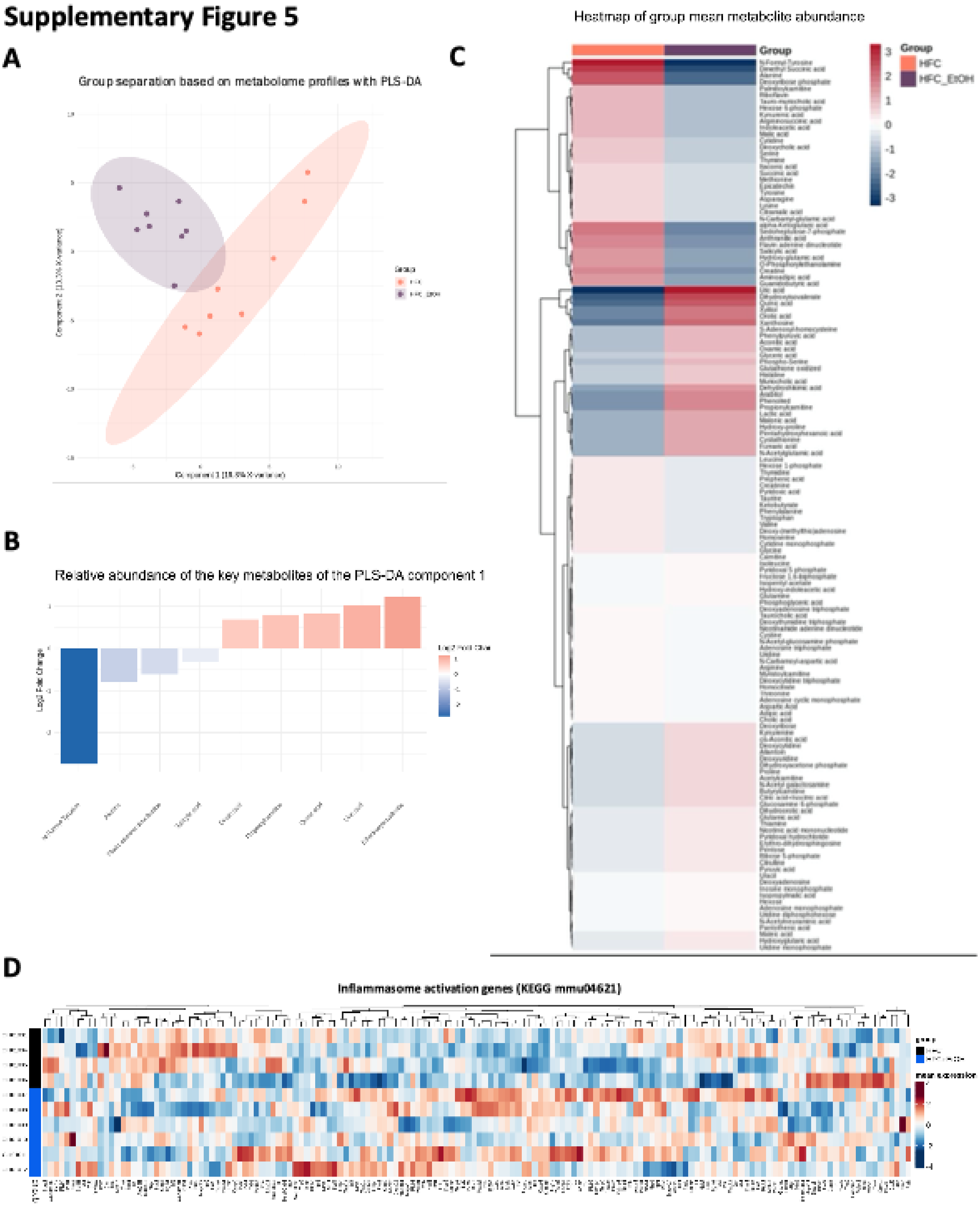

Supplementary Figure 6

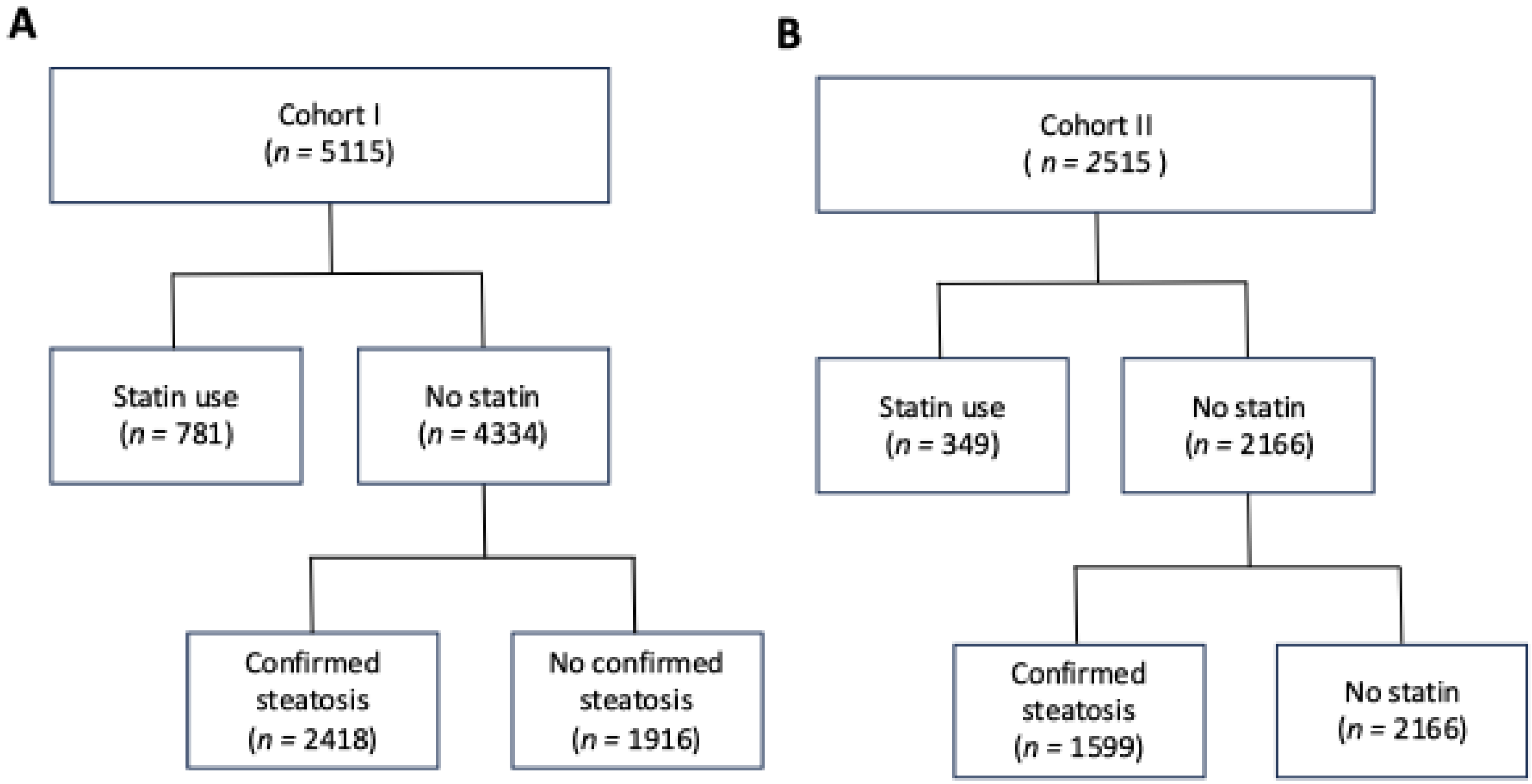

Supplementary figure legends

Fig. S1.

(A) Weekly bodyweight from day 0 through week 8. **(B-C)** Hepatic gene expression. **(D-E)** Lipid levels in distal small intestinal content. **(F)** Small intestinal gene expression. **(G)** Representative images of H&E-stained liver sections. **(H)** Principal component projection of bulk RNA sequencing data from HFC-fed *Ldlr*$^{-/-}$ mice with regular or ethanol-containing drinking water. The two components with the highest attributed variation are displayed. **(I)** Volcano plot of differentially expressed genes, indicating upregulation (right) and downregulation (left) in ethanol-consuming *Ldlr*$^{-/-}$ mice compared to controls. (**J, K**) Heatmap representation of the genes involved in cholesterol metabolism (KEGG pathway accession ko04979). **(J)** Average and **(K)** individual values for each group are presented.
Data are mean ± SEM (n = 4–11 for the HFC group and n = 5–15 for the HFC+EtOH group). mRNA was normalized to *18S* and expressed relative to HFC-fed *Ldlr*$^{-/-}$ mice on regular drinking water. Statistical significance was determined using a two-tailed unpaired Student's t-test; *$p \leq 0.05$, ** $p \leq 0.0$.

Fig. S2.

(A) Heatmap depicting the expression levels of all cytokine genes from the BioMart database and **(B)** of immune-related genes in livers of HFC-fed *Ldlr*$^{-/-}$ mice with regular and ethanol-containing drinking water. Individual values for each group are presented. **(C)** Gene set enrichment analysis (Reactome pathways) based on all hepatic DEG between the two groups. **(D)** Systemic IgM, **(E)** IgG1, and **(F)** IgA concentrations.
Data are mean ± SEM (n = 4–11 for the HFC group and n = 5–19 for the HFC+EtOH group).

Fig. S3.

(A) Representative flow cytometry gating plots for hepatic Ly6C^{high} monocytes. (**B-E**) Quantification of systemic immune cell populations by flow cytometry: monocytes, B cells**,** T cells, and neutrophils. **(F)** Quantification of aortic root lesion size, calculated as the average lesion size (µm²) from nine sections throughout the aortic root. **(G)** Area under the curve (AUC) for plasma cholesterol concentrations measured at baseline, week 4 and week 8 of the dietary intervention in selected mice with comparable cholesterol exposure over time. **(H)** Average aortic root lesion size (µm² per section) in selected mice with similar cholesterol exposure during the study. **(I-Q)** 16 weeks dietary intervention. **(I)** Weekly bodyweight from day 0 through week 16. **(J)** Plasma ethanol levels at the study endpoint. **(K)** Liver weight. **(L)** Representative images of H&E-stained liver sections. **(M)** Hepatic cholesterol and **(N)** triglyceride levels. **(O)** Representative images showing Oil Red O staining of liver sections. **(P)** Quantification of hepatic monocytes, Ly6C^{high} monocytes (, B cells ($CD45^{+}B220^{+}CD3^{-}$), T cells, and neutrophils. **(Q)** Hepatic gene expression.
(A-H) Data are mean ± SEM (n = 7–11 for the HFC group and n = 9–11 for the HFC+EtOH group). (I–Q) Data are mean ± SEM (n = 5–6 for the HFC group and n = 7 for the HFC+EtOH group). mRNA was normalized to *18S* and expressed relative to HFC-fed *Ldlr*$^{-/-}$ mice on regular drinking water. Statistical significance was determined using a two-tailed unpaired Student's t test; *$p \leq 0.05$, ** $p \leq 0.0$.

Fig. S4.

(A-F) 8-week dietary intervention. **(A)** 16S rRNA sequencing of fecal microbiota at the end of the study, illustrating the relative abundance of indicated bacterial genera. **(B)** Principal component analysis of 16S rRNA sequencing data from fecal bacterial microbiome. **(C)** Gene expression of tight junction genes and **(D)** of mucus barrier genes in the small intestine. **(E)** Hepatic Occludin western blot quantification with representative blots. β-Actin was used as loading control. **(F)** Systemic LPS levels. (**G-I**) 8-week dietary intervention with CONV-R and GF *Ldlr*$^{-/-}$ mice. **(G)** Body weight of CONV-R and GF *Ldlr*$^{-/-}$ mice at the study endpoint. **(H)** Plasma ethanol levels of CONV-R and GF *Ldlr*$^{-/-}$ mice. **(I)** Quantification of hepatic immune cell populations by flow cytometry: Kupffer cells, B cells, T cells, and hepatic neutrophils. **(J-L)** 8-week dietary intervention at timepoint 4 weeks. **(J)** Quantification of circulating Ly6C^{high} monocytes as a percentage of all monocytes, determined by flow cytometry. **(K)** Quantification of systemic cholesterol and **(L)** triglyceride levels. (**M-O**) 4-week dietary intervention with antibiotics-mediated microbiome depletion. **(M)** Representative images indicating successful antibiotics-mediated microbiome depletion. Anaerobic bacteria: control vs. antibiotic-treated HFC and HFC+EtOH groups (left); Aerobic bacteria: control vs antibiotic-treated HFC and HFC+EtOH groups (right). **(N)** Body weight of antibiotic-treated *Ldlr*$^{-/-}$ mice at the study endpoint. **(O)** Plasma ethanol levels of antibiotic-treated *Ldlr*$^{-/-}$ mice.

(A-F, J-K) Data are mean ± SEM (*n* = 8–12 for the HFC group and *n* = 11–12 for the HFC+EtOH group). (G–I) Data are presented as mean ± SEM (*n* = 12 for the CONV-R group and *n* = 12 for the GF group). (N, O) Data are presented as mean ± SEM (*n* = 6–7 for the antibiotic-treated HFC group *n* = 5-7 antibiotic-treated HFC+EtOH group). mRNA was normalized to *18S* and expressed relative to to HFC-fed *Ldlr*$^{-/-}$ mice on regular drinking water. Statistical significance was determined using a two-tailed unpaired Student's t test; ** $p \leq 0.01$, **$p \leq 0.001$

Fig. S5.

(A) The partial least squares discrimination analysis (PLS-DA) of the systemic metabolome profile of HFC-fed *Ldlr*$^{-/-}$ mice with regular and ethanol-containing drinking water. The two components with the highest attributed variation are displayed. **(B)** Relative abundance of key metabolites of the main component of PLS-DA in plasma of ethanol-consuming HFC-fed *Ldlr*$^{-/-}$ mice compared to controls. **(C)** Heatmap displaying the mean plasma metabolite abundance within the two experimental groups. **(D)** Heatmap representation of the genes involved in inflammasome activation (KEGG pathway accession mmu04621) in livers of mice depicted as individual values for each group.

Fig. S6.

(A) Flow chart describing Cohort I. **(B)** Flow chart describing Cohort II.

Supplementary tables

Table S1. Murine blood parameters after 16 weeks dietary intervention.

Parameter	Unit	HFC	HFC + EtOH	*P* value
**White blood cells**	10^3/µL	10.8 (2.1)	14.4 (3.5)	0.0513
**Lymphocytes**	10^3/µL	7.9 (1.5)	9.6 (2.0)	0.1018
**Lymphocytes**	%	73.0 (3.8)	67.5 (3.0)	**0.0146**
**Monocytes**	10^3/µL	0.75 (0.2)	1.1 (0.3)	**0.0451**
**Monocytes**	%	7.2 (1.1)	8.0 (0.6)	0.1232
**Granulocytes**	10^3/µL	2.2 (0.5)	3.7 (1.2)	**0.0232**
**Granulocytes**	%	19.7 (2.9)	24.4 (2.7)	**0.0124**
**Eosinophils**	%	1.9 (0.4)	2.3 (0.1)	**0.035**
**Red blood cells**	10^6/µL	15.9 (1.6)	16.0 (1.1)	0.2985
**Hemoglobin**	g/dL	24.5 (2.1)	24.6 (1.9)	0.9576
**Hematocrit**	%	83.78 (7.2)	84.3 (6.9)	0.8847
**Mean corpuscular volume**	µm	52.5 (1.5)	52.6 (1.1)	0.9244
**Mean corpuscular hemoglobin**	pg	15.4 (0.6)	15.4 (0.4)	0.7685
**Mean corpuscular hemoglobin concentration**	g/dL	29.3 (0.6)	29.2 (0.3)	0.6021
**Red blood cell distribution width**	%	15.2 (0.6)	15.1 (0.2)	0.525
**Platelets**	10^3/µL	1226 (270.3)	1165.4 (260.5)	0.6891
**Mean platelet volume**	µm^3	6.3 (0.2)	6.3 (0.1)	0.77
**Monocyte/Lymphocyte**	ratio	0.1 (0.02)	0.1 (0.01)	0.065

Statistical significance was determined using two-tailed, unpaired Student's *t*-tests.

Table S2. Characteristics of 5115 subjects of the general population including statin users (Cohort I), stratified by self-reported alcohol intake into abstainers, low consumption (<20/30g/d), moderate consumption (20-50/30-60g/d) and high consumption (>50/60g/d), respectively.

**Parameter**	**Overall (N=5115)**	**Abstinence (n=1912)**	**<20/30g/d (n=2650)**	**20/30-50/60g/d (n=507)**	**>50/60g/d (n=46)**	***P* value**
Age, years	59.4 (8.42)	60.1 (8.63)	58.6 (8.25)	60.7 (8.20)	60.5 (8.21)	**<0.001**
Male sex	2700 (52.8%)	606 (31.7%)	1727 (65.2%)	331 (65.3%)	36 (78.3%)	**<0.001**
BMI, kg/m²	27.2 (4.68)	27.7 (5.21)	26.9 (4.37)	26.7 (3.90)	28.3 (4.68)	**<0.001**
BMI ≥30 kg/m²	1200 (23.5%)	545 (28.5%)	544 (20.5%)	96 (18.9%)	15 (32.6%)	**<0.001**
Abdominal obesity	3782 (73.9%)	1473 (77.0%)	1887 (71.2%)	385 (75.9%)	37 (80.4%)	**<0.001**
Alcohol, g/week	20.0 [0-100]	0 [0-0]	80.0 [20.0-100]	280 [160-280]	500 [440-560]	**<0.001**
Diabetes	896 (17.5%)	383 (20.0%)	405 (15.3%)	97 (19.1%)	11 (23.9%)	**<0.001**
Uric acid, mg/dL	5.77 (1.53)	5.47 (1.48)	5.88 (1.50)	6.18 (1.57)	6.85 (2.19)	**<0.001**
AST, U/L	21.0 [17.0-25.0]	20.0 [17.0-24.0]	21.0 [18.0-26.0]	22.0 [18.0-28.0]	24.0 [20.0-31.0]	**<0.001**
AST xULN	0.486 [0.400-0.600]	0.514 [0.429-0.629]	0.480 [0.400-0.600]	0.514 [0.400-0.629]	0.520 [0.440-0.680]	**<0.001**
ALT, U/L	21.0 [16.0-30.0]	19.0 [15.0-28.0]	21.0 [16.0-30.3]	23.0 [17.0-34.0]	26.0 [18.0-33.0]	**<0.001**
ALT xULN	0.500 [0.371-0.680]	0.514 [0.380-0.686]	0.486 [0.371-0.660]	0.517 [0.380-0.740]	0.540 [0.429-0.720]	**0.050**
GGT, U/L	26.0 [18.0-44.0]	22.0 [16.0-35.0]	28.0 [19.0-45.0]	35.0 [22.0-59.0]	57.0 [33.0-108]	**<0.001**
GGT xULN	0.525 [0.367-0.825]	0.483 [0.350-0.750]	0.533 [0.375-0.825]	0.633 [0.433-1.09]	1.10 [0.700-1.80]	**<0.001**
Glucose, mg/dL	102 (23.9)	103 (24.3)	102 (22.8)	104 (28.1)	106 (20.6)	**0.043**
HbA1c (%)	5.58 (0.577)	5.65 (0.605)	5.53 (0.556)	5.55 (0.523)	5.59 (0.884)	**<0.001**
Cholesterol, mg/dL	222 (43.2)	220 (44.1)	222 (41.7)	228 (46.1)	229 (51.1)	**0.003**
HDL cholesterol, mg/dL	58.4 (16.6)	57.2 (15.6)	58.7 (16.9)	60.6 (18.6)	62.0 (19.6)	**<0.001**

LDL cholesterol, mg/dL	143 (39.3)	143 (39.8)	142 (38.2)	146 (41.7)	144 (49.3)	0.325
Triglycerides, mg/dL	107 [79.0-150]	107 [79.0-145]	106 [78.0-150]	116 [84.0-163]	135 [98.8-182]	**0.001**
SCORE2, points[1]	4.9 [3.0-7.4]	4.5 [2.7-6.7]	5.0 [3.0-7.5]	5.9 [3.9-8.8]	6.0 [4.7-8.2]	**<0.001**
FRS, points[2]	0.143 [0.0806-0.251]	0.127 [0.0719-0.225]	0.147 [0.0831-0.254]	0.186 [0.103-0.304]	0.204 [0.134-0.302]	**<0.001**
CRP, mg/dL	0.190 [0.100-0.360]	0.200 [0.100-0.403]	0.170 [0.100-0.310]	0.200 [0.100-0.300]	0.200 [0.100-0.500]	**<0.001**
Monocytes, %	7.30 [6.00-8.80]	6.80 [5.70-8.40]	7.50 [6.30-8.90]	7.70 [6.60-8.98]	7.15 [6.25-9.25]	**<0.001**
Monocytes, G/L	0.420 [0.330-0.520]	0.410 [0.320-0.500]	0.430 [0.340-0.540]	0.425 [0.363-0.575]	0.455 [0.320-0.688]	**0.007**
Monocyte to lymphocyte ratio	0.243 [0.191-0.318]	0.231 [0.189-0.297]	0.250 [0.191-0.329]	0.259 [0.210-0.336]	0.314 [0.220-0.387]	**0.003**

[1] Only calculated in 2576 patients aged 40-69, without diabetes and without established cardiovascular disease; [2] Only calculated in patients without established cardiovascular disease. Group comparisons used Kruskal–Wallis tests for continuous variables and chi-square tests for categorical variables; for normally-distributed variables (uric acid and total cholesterol), one-way ANOVA was used.

Table S3. Characteristics of 1916 subjects of the general population (Cohort I) with confirmed hepatic steatosis on ultrasound (n=1916) excluding statin users, stratified by self-reported alcohol intake into abstainers, low consumption (<20/30g/d), moderate consumption (20-50/30-60g/d) and high consumption (>50/60g/d), respectively.

**Parameter**	**Overall (N=1916)**	**Abstinence (n=672)**	**<20/30g/d (n=1001)**	**20/30-50/60g/d (n=213)**	**>50/60g/d (n=30)**	***P* value**
Age, years	59.6 (8.12)	60.5 (8.23)	58.8 (7.92)	60.5 (8.24)	61.7 (8.87)	**<0.001**
Male sex	1198 (62.5%)	263 (39.1%)	759 (75.8%)	154 (72.3%)	22 (73.3%)	**<0.001**
BMI, kg/m²	29.4 (4.60)	30.5 (4.97)	29.0 (4.32)	28.2 (3.92)	29.2 (5.04)	**<0.001**
BMI ≥30 kg/m²	740 (38.6%)	331 (49.3%)	337 (33.7%)	61 (28.6%)	11 (36.7%)	**<0.001**
Abdominal obesity	1633 (85.2%)	595 (88.5%)	834 (83.3%)	177 (83.1%)	27 (90.0%)	**0.002**
Alcohol, g/week	40.0 [0-112]	0 [0-0]	80.0 [20.0-100]	280 [220-300]	500 [440-560]	**<0.001**
Diabetes	392 (20.5%)	163 (24.3%)	168 (16.8%)	53 (24.9%)	8 (26.7%)	**0.002**
Uric acid, mg/dL	6.30 (1.44)	6.00 (1.37)	6.41 (1.41)	6.64 (1.44)	6.96 (2.48)	**<0.001**
AST, U/L	22.0 [18.0-28.0]	21.0 [18.0-26.0]	22.0 [19.0-28.0]	24.0 [19.0-31.0]	23.0 [19.3-28.5]	**<0.001**
AST xULN	0.514 [0.420-0.640]	0.543 [0.440-0.657]	0.486 [0.400-0.629]	0.540 [0.440-0.665]	0.493 [0.425-0.595]	**<0.001**
ALT, U/L	26.0 [19.0-36.0]	24.0 [18.0-34.0]	27.0 [20.0-37.0]	29.0 [20.0-40.0]	24.0 [17.3-30.8]	**<0.001**
ALT xULN	0.600 [0.440-0.820]	0.600 [0.457-0.800]	0.580 [0.440-0.820]	0.620 [0.480-0.900]	0.517 [0.407-0.635]	0.065
GGT, U/L	33.0 [22.0-54.0]	26.0 [19.0-42.0]	36.0 [25.0-56.0]	45.0 [28.0-74.0]	56.5 [44.3-101]	**<0.001**
GGT xULN	0.650 [0.433-1.00]	0.550 [0.400-0.867]	0.650 [0.450-1.02]	0.808 [0.550-1.24]	1.03 [0.788-1.69]	**<0.001**
Glucose, mg/dL	105 (22.0)	107 (24.2)	104 (20.1)	107 (22.8)	108 (22.7)	**0.047**
HbA1c (%)	5.65 (0.572)	5.76 (0.646)	5.59 (0.489)	5.64 (0.576)	5.63 (1.02)	**<0.001**
Cholesterol, mg/dL	225 (42.4)	220 (42.7)	226 (40.6)	235 (46.0)	235 (53.2)	**0.001**
HDL cholesterol, mg/dL	53.6 (15.1)	51.8 (12.9)	53.8 (15.0)	57.1 (19.3)	64.5 (22.2)	**<0.001**

LDL cholesterol, mg/dL	149 (38.1)	147 (38.0)	149 (36.8)	154 (41.6)	151 (52.4)	0.237
Triglycerides, mg/dL	127 [94.0-175]	124 [93.0-169]	128 [95.0-178]	133 [96.8-192]	129 [89.0-172]	0.367
SCORE2, points[1]	7.00 [4.50-10.2]	6.40 [4.20-9.70]	7.10 [4.50-10.2]	8.00 [5.15-11.4]	7.50 [5.40-12.7]	**0.007**
FRS, points[2]	0.180 [0.107-0.293]	0.158 [0.0961-0.272]	0.189 [0.114-0.292]	0.212 [0.134-0.355]	0.185 [0.133-0.281]	**<0.001**
CRP, mg/dL	0.230 [0.120-0.450]	0.290 [0.140-0.500]	0.210 [0.100-0.400]	0.200 [0.100-0.400]	0.290 [0.145-0.613]	**<0.001**
Monocytes, %	7.40 [6.25-8.80]	6.80 [5.70-8.40]	7.50 [6.40-9.08]	8.05 [6.98-9.03]	7.05 [6.08-8.35]	**<0.001**
Monocyte to lymphocyte ratio	0.248 [0.195-0.322]	0.226 [0.189-0.303]	0.263 [0.200-0.336]	0.273 [0.210-0.340]	0.244 [0.184-0.303]	**0.015**

[1] Only calculated in patients aged 40-69, without diabetes and without established cardiovascular disease; [2] Only calculated in patients without established cardiovascular disease. Group comparisons used Kruskal–Wallis tests for continuous variables and chi-square tests for categorical variables; for normally-distributed variables (uric acid and total cholesterol), one-way ANOVA was used.

Table S4. Characteristics of 2515 individuals referred to a hepatology outpatient clinic (Cohort II), stratified by self-reported alcohol intake into abstainers, low consumption (<20/30g/d), moderate consumption (20-50/30-60g/d) and high consumption (>50/60g/d), respectively.

**Parameter**	**Overall (N=2515)**	**Abstinence (n=730)**	**<20/30g/d (n=1290)**	**20/30-50/60g/d (n=290)**	**>50/60g/d (n=205)**	***P* value**
Age, years	52.5 (15.2)	51.1 (15.8)	51.5 (15.1)	56.4 (14.4)	57.8 (12.4)	**<0.001**
Male sex	1422 (56.5%)	275 (37.7%)	778 (60.3%)	194 (66.9%)	175 (85.4%)	**<0.001**
BMI, kg/m²	27.3 (7.65)	27.5 (5.62)	27.2 (9.32)	26.8 (4.19)	27.2 (5.35)	0.600
BMI ≥30 kg/m²	615 (24.5%)	219 (30.0%)	294 (22.8%)	54 (18.6%)	48 (23.4%)	**<0.001**
Diabetes	361 (14.4%)	122 (16.7%)	171 (13.3%)	29 (10.0%)	39 (19.0%)	**0.007**
Uric acid, mg/dL	5.79 (1.53)	5.46 (1.47)	5.80 (1.48)	6.08 (1.63)	6.50 (1.55)	**<0.001**
AST, U/L	31.0 [24.0-44.0]	30.0 [23.0-42.0]	31.0 [24.0-42.0]	33.0 [25.0-46.0]	42.0 [31.0-67.0]	**<0.001**
ALT, U/L	39.0 [26.0-63.0]	37.0 [24.3-63.0]	39.0 [26.0-63.0]	41.5 [28.0-63.8]	44.0 [30.0-62.0]	0.228
GGT, U/L	62.0 [30.0-134]	51.5 [24.0-117]	58.0 [28.0-118]	75.0 [38.0-156]	179 [73.0-407]	**<0.001**
Glucose, mg/dL	100 (25.7)	101 (29.3)	98.9 (23.8)	101 (22.8)	106 (27.0)	**0.002**
HbA1c (%)	5.48 (0.732)	5.59 (0.883)	5.46 (0.651)	5.42 (0.548)	5.35 (0.809)	**<0.001**
Cholesterol, mg/dL	203 (48.0)	198 (48.3)	205 (45.4)	209 (46.7)	203 (61.6)	**<0.001**
HDL cholesterol, mg/dL	58.0 (19.0)	56.8 (18.5)	57.8 (18.7)	60.2 (19.3)	60.3 (22.0)	**0.019**
LDL cholesterol, mg/dL	129 (42.7)	125 (42.0)	131 (40.8)	133 (42.8)	123 (53.6)	**<0.001**
Triglycerides, mg/dL	113 [80.0-165]	109 [80.0-159]	114 [79.0-167]	116 [81.0-160]	118 [90.0-186]	0.137
CRP, mg/dL	0.170 [0.100-0.400]	0.190 [0.100-0.420]	0.150 [0.100-0.360]	0.170 [0.100-0.388]	0.200 [0.100-0.540]	**0.007**
Monocytes, G/L	0.500 [0.400-0.635]	0.490 [0.390-0.618]	0.490 [0.390-0.620]	0.530 [0.410-0.660]	0.610 [0.490-0.760]	**<0.001**
Monocyte to lymphocyte ratio	0.278 [0.218-0.366]	0.257 [0.205-0.343]	0.277 [0.217-0.358]	0.302 [0.234-0.403]	0.350 [0.264-0.482]	**<0.001**

Group comparisons used Kruskal–Wallis tests for continuous variables and chi-square tests for categorical variables; for normally-distributed variables (uric acid and total cholesterol), one-way ANOVA was used.

Table S5. Characteristics of 1599 individuals referred to a hepatology outpatient clinic (Cohort II) with hepatic steatosis as assessed by controlled attenuation parameter (CAP) ≥248dB/m, stratified by self-reported alcohol intake into abstainers, low consumption (<20/30g/d), moderate consumption (20-50/30-60g/d) and high consumption (>50/60g/d), respectively.

Parameter	Overall (N=1599)	Abstinence (n=433)	<20/30g/d (n=811)	20/30-50/60g/d (n=202)	>50/60g/d (n=153)	***P* value**
Age, years	53.8 (14.2)	52.7 (14.6)	52.7 (14.2)	56.9 (14.1)	58.1 (12.1)	**<0.001**
Male sex	975 (61.0%)	180 (41.6%)	520 (64.1%)	146 (72.3%)	129 (84.3%)	**<0.001**
BMI, kg/m²	28.8 (8.64)	29.6 (5.18)	29.0 (11.0)	27.7 (3.88)	27.6 (5.51)	**0.020**
BMI ≥30 kg/m²	529 (33.1%)	182 (42.0%)	262 (32.3%)	45 (22.3%)	40 (26.1%)	**<0.001**
Diabetes	282 (17.6%)	94 (21.7%)	137 (16.9%)	24 (11.9%)	27 (17.6%)	**0.032**
Uric acid, mg/dL	6.07 (1.48)	5.77 (1.40)	6.07 (1.45)	6.30 (1.63)	6.57 (1.48)	**<0.001**
AST, U/L	33.0 [25.0-46.0]	32.0 [24.0-44.0]	32.0 [25.0-44.0]	33.0 [26.0-46.0]	43.0 [31.0-68.0]	**<0.001**
ALT, U/L	43.0 [29.0-66.0]	40.0 [27.0-65.0]	44.0 [29.0-67.0]	44.0 [32.0-66.0]	44.0 [30.0-62.0]	0.554
GGT, U/L	67.0 [35.0-145]	58.0 [29.0-120]	62.0 [35.0-122]	78.0 [38.3-159]	191 [73.0-388]	**<0.001**
Glucose, mg/dL	104 (27.7)	105 (32.9)	103 (26.3)	102 (18.5)	107 (28.3)	0.113
HbA1c (%)	5.57 (0.772)	5.71 (0.904)	5.54 (0.717)	5.49 (0.506)	5.37 (0.873)	**<0.001**
Cholesterol, mg/dL	204 (47.4)	195 (48.4)	208 (45.5)	214 (44.7)	200 (53.4)	**<0.001**
HDL cholesterol, mg/dL	54.8 (18.0)	52.3 (16.4)	53.9 (16.8)	59.0 (19.6)	61.2 (23.3)	**<0.001**
LDL cholesterol, mg/dL	131 (42.3)	125 (42.3)	134 (41.0)	138 (40.2)	121 (48.3)	**<0.001**
Triglycerides, mg/dL	129 [93.0-184]	127 [93.0-179]	133 [94.0-191]	129 [90.3-171]	119 [91.0-185]	0.538
CRP, mg/dL	0.200 [0.100-0.410]	0.220 [0.100-0.500]	0.180 [0.100-0.370]	0.190 [0.100-0.400]	0.200 [0.100-0.410]	**0.033**
Monocytes, G/L	0.520 [0.420-0.640]	0.490 [0.390-0.610]	0.510 [0.410-0.630]	0.530 [0.420-0.668]	0.600 [0.490-0.720]	**<0.001**
Monocytes, %	8.1 [6.9-9.5]	7.6 [6.4-9.0]	8.1 [7.0-9.4]	8.5 [7.2-9.9]	9.1 [7.7-10.4]	**<0.001**

Monocyte to lymphocyte ratio	0.279 [0.219-0.365]	0.248 [0.199-0.328]	0.279 [0.222-0.359]	0.304 [0.240-0.407]	0.350 [0.265-0.462]	**<0.001**

Group comparisons used Kruskal–Wallis tests for continuous variables and chi-square tests for categorical variables; for normally-distributed variables (uric acid and total cholesterol), one-way ANOVA was used.

Supplementary references

1. Semmler G, Wernly S, Bachmayer S, Leitner I, Wernly B, Egger M, et al. Metabolic Dysfunction-Associated Fatty Liver Disease (MAFLD)-Rather a Bystander Than a Driver of Mortality. J Clin Endocrinol Metab. 2021;106(9):2670-7.
2. Schonfeld M, O'Neil M, Villar MT, Artigues A, Averilla J, Gunewardena S, et al. A Western diet with alcohol in drinking water recapitulates features of alcohol-associated liver disease in mice. Alcohol Clin Exp Res. 2021;45(10):1980-93.
3. Gruber S, Hendrikx T, Tsiantoulas D, Ozsvar-Kozma M, Göderle L, Mallat Z, et al. Sialic Acid-Binding Immunoglobulin-like Lectin G Promotes Atherosclerosis and Liver Inflammation by Suppressing the Protective Functions of B-1 Cells. Cell Rep. 2016;14(10):2348-61.
4. Mieczkowski K, Bakiri L, Griss J, Wagner EF. A Sex-Specific Anti-Inflammatory Role for p62 in Psoriasis-Like Disease. J Invest Dermatol. 2025;145(11):2763-74.e11.
5. Burger K, Jung F, Baumann A, Brandt A, Staltner R, Sánchez V, et al. TNFα is a key trigger of inflammation in diet-induced non-obese MASLD in mice. Redox Biol. 2023;66:102870.
6. Brandt A, Jin CJ, Nolte K, Sellmann C, Engstler AJ, Bergheim I. Short-Term Intake of a Fructose-, Fat- and Cholesterol-Rich Diet Causes Hepatic Steatosis in Mice: Effect of Antibiotic Treatment. Nutrients. 2017;9(9).
7. Bieghs V, Van Gorp PJ, Wouters K, Hendrikx T, Gijbels MJ, van Bilsen M, et al. LDL receptor knock-out mice are a physiological model particularly vulnerable to study the onset of inflammation in non-alcoholic fatty liver disease. PLoS One. 2012;7(1):e30668.
8. Busch CJ, Hendrikx T, Weismann D, Jackel S, Walenbergh SM, Rendeiro AF, et al. Malondialdehyde epitopes are sterile mediators of hepatic inflammation in hypercholesterolemic mice. Hepatology. 2017;65(4):1181-95.
9. Hoebinger C, Rajcic D, Silva B, Hendrikx T. Chronic-binge ethanol feeding aggravates systemic dyslipidemia in Ldlr(-/-) mice, thereby accelerating hepatic fibrosis. Front Endocrinol (Lausanne). 2023;14:1148827.
10. Piollet M, Porsch F, Rizzo G, Kapser F, Schulz DJJ, Kiss MG, et al. TREM2 protects from atherosclerosis by limiting necrotic core formation. Nat Cardiovasc Res. 2024;3(3):269-82.
11. Drost HG, Paszkowski J. Biomartr: genomic data retrieval with R. Bioinformatics. 2017;33(8):1216-7.
12. Love MI, Huber W, Anders S. Moderated estimation of fold change and dispersion for RNA-seq data with DESeq2. Genome Biol. 2014;15(12):550.
13. Stephens M. False discovery rates: a new deal. Biostatistics. 2017;18(2):275-94.
14. Ignatiadis N, Klaus B, Zaugg JB, Huber W. Data-driven hypothesis weighting increases detection power in genome-scale multiple testing. Nat Methods. 2016;13(7):577-80.
15. Wu T, Hu E, Xu S, Chen M, Guo P, Dai Z, et al. clusterProfiler 4.0: A universal enrichment tool for interpreting omics data. Innovation (Camb). 2021;2(3):100141.
16. Kolberg L, Raudvere U, Kuzmin I, Vilo J, Peterson H. gprofiler2 -- an R package for gene list functional enrichment analysis and namespace conversion toolset g:Profiler. F1000Res. 2020;9.
17. Hänzelmann S, Castelo R, Guinney J. GSVA: gene set variation analysis for microarray and RNA-seq data. BMC Bioinformatics. 2013;14:7.
18. Bueschl C, Doppler M, Varga E, Seidl B, Flasch M, Warth B, et al. PeakBot: machine-learning-based chromatographic peak picking. Bioinformatics. 2022;38(13):3422-8.
19. Castellano-Escuder P, González-Domínguez R, Carmona-Pontaque F, Andrés-Lacueva C, Sánchez-Pla A. POMAShiny: A user-friendly web-based workflow for metabolomics and proteomics data analysis. PLoS Comput Biol. 2021;17(7):e1009148.

20. van den Berg RA, Hoefsloot HC, Westerhuis JA, Smilde AK, van der Werf MJ. Centering, scaling, and transformations: improving the biological information content of metabolomics data. BMC Genomics. 2006;7:142.
21. Chong J, Xia J. MetaboAnalystR: an R package for flexible and reproducible analysis of metabolomics data. Bioinformatics. 2018;34(24):4313-4.
22. Pjevac P, Hausmann B, Schwarz J, Kohl G, Herbold CW, Loy A, et al. An Economical and Flexible Dual Barcoding, Two-Step PCR Approach for Highly Multiplexed Amplicon Sequencing. Front Microbiol. 2021;12:669776.
23. Callahan BJ, McMurdie PJ, Rosen MJ, Han AW, Johnson AJ, Holmes SP. DADA2: High-resolution sample inference from Illumina amplicon data. Nat Methods. 2016;13(7):581-3.
24. Callahan BJ, Sankaran K, Fukuyama JA, McMurdie PJ, Holmes SP. Bioconductor Workflow for Microbiome Data Analysis: from raw reads to community analyses. F1000Res. 2016;5:1492.
25. Quast C, Pruesse E, Yilmaz P, Gerken J, Schweer T, Yarza P, et al. The SILVA ribosomal RNA gene database project: improved data processing and web-based tools. Nucleic Acids Res. 2013;41(Database issue):D590-6.
