# Supplementary material for: Alcohol intake reprograms hepatic immune-metabolic circuits to exacerbate murine atherosclerosis and human cardiovascular risk
Source: JHEP Rep. 2026 Jun 19;8(9):101932. doi: 10.1016/j.jhepr.2026.101932 (PMC13482647; doi:10.1016/j.jhepr.2026.101932)
Supplement: Supplementary file 2 [file mmc2.pdf]

JHEP|Reports

JHEP Reports

CTAT methods

Tables for a "Complete, Transparent, Accurate and Timely account" (CTAT) are now mandatory for all revised submissions. The aim is to enhance the reproducibility of methods.

- Only include the parts relevant to your study
- Refer to the CTAT in the main text as 'Supplementary CTAT Table'
- Do not add subheadings
- Add as many rows as needed to include all information
- Only include one item per row

If the CTAT form is not relevant to your study, please outline the reasons why:

1.1 Antibodies

Name	Citation/Method	Supplier	Cat no.	Clone no.
Anti-Ly6G	IHC	BD PharMingen	NIMP-R14	MA1-40038
Anti-F4/80	IHC	Cell signaling tech	7007S cell signaling	D2S9R
Anti-Mac2	IHC	BioLegend	B125401	M3/38
Goat anti-rabbit IgG biotinylated	IHC	Vector Laboratories	BA-1000	
Anti-Mouse IgM (coating)	ELISA	Sigma-Aldrich	AP500	polyclonal
Anti-Mouse IgM (detection)	ELISA	Sigma-Aldrich	A9688	polyclonal
Anti-Mouse IgG1 (coating)	ELISA	BioLegend	406602	RMG1-1
Anti-Mouse IgG1 (detection)	ELISA	Becton Dickinson	553441	A85-1
Anti-Mouse IgA (coating)	ELISA	Becton Dickinson	556969	C10-3
Anti-Mouse IgA (detection)	ELISA	Becton Dickinson	556978	C10-1
Anti-mouse IgG1 Antibody	Western Blot	BioLegend	406602	RMG1-1
Anti-Srebp1	Western Blot	Abcam	ab3259	2A4
Anti-Srebp2	Western Blot	Abcam		not specified
Anti-NLRP3	Western Blot	eubio		not specified
Anti-β-Actin	Western Blot	Abcam	ab8226	not specified
Anti-Tubulin	Western Blot	Abcam		not specified
Anti-CD16/32	Flow cytometry	eBioscience	14-0161-85	93
Anti-Ly6C (BV605)	Flow cytometry	BioLegend	128036	HK1.4
Anti-CD45 (PerCP-Cy5.5)	Flow cytometry	eBioscience	45-0451-82	30-F11
Anti-CD11b (FITC)	Flow cytometry	BioLegend	101205	M1/70
Anti-CD3e (PE)	Flow cytometry	eBioscience	12-0031-82	145-2C11

Anti-Ly6G (PE-Cy7)	Flow cytometry	BD Biosciences	560601	1A8
Anti-CD11c (APC-eFluor780)	Flow cytometry	eBioscience	47-0114-82	N418
Anti-B220 (AlexaFluor700)	Flow cytometry	BioLegend	B103231	RA3-6B2
Anti-CD115 (PerCP-Cy5.5)	Flow cytometry	BioLegend	135525	AFS98

1.2 Cell lines

Name	Citation	Supplier	Cat no.	Passage no.	Authentication test method
NA					

1.3 Organisms

Name	Supplier	Strain	Sex	Age	Overall n number
mouse	Medical University of Vienna	*Ldlr*$^{-/-}$	female	9-13 weeks	5-22; see figure legends
Mouse (GF)	University Medical Center Mainz	*Ldlr*$^{-/-}$	female	11-14 weeks	11-13; see figure legends
Mouse (CONV-R)	University Medical Center Mainz	*Ldlr*$^{-/-}$	female	11-14 weeks	11-12; see figure legends

1.4 Sequence based reagents

Target Gene	Forward Sequence (5'-3')	Reverse Sequence (5'-3')	Supplier
Abca1	GAGCAAAGCCAAGCATCTTC	AGCAGGGACCACATAATTGC	Sigma-Aldrich
Abcg4	CTGAGCTCTTCCAACACTTCG	GCCACAGAACACCAACTCTC	Sigma-Aldrich
Abcg8	CTCACCTACCAGGTGGACAT	GCATCTGTCCACTCCTCACT	Sigma-Aldrich
Adh1	GGGTTCTCAACTGGCTATGG	ACAGACAGACCGACACCTCC	Sigma-Aldrich
Aim2	AGGCTGCTACAGAAGTCTGTCC	TCAGCACCGTGACAACAAGTGG	Sigma-Aldrich
Casp1	GGCACATTTCCAGGACTGACTG	GCAAGACGTGTACGAGTGGTTG	Sigma-Aldrich
Ccl2	AGGTCCCTGTCATGCTTCTG	TCTGGACCCATTCCTTCTTG	Sigma-Aldrich
Cd36	GCCAAGCTATTGCGACATGA	AAAAGAATCTCAATGTCCGAGACTTT	Sigma-Aldrich
Cldn1	ATGCCAATTACCATCAAGGC	GAGGGACTGTGGATGTCCTG	Sigma-Aldrich

Cldn2	TTCTTGGATCCGAGCCTCTA	TCCAGAGCTCTTCGAAAGGA	Sigma-Aldrich
Cxcl1	GCTGGGATTCACCTCAAGAA	TCTCCGTTACTTGGGGACAC	Sigma-Aldrich
Cxcl2	AGTGAACTGCGCTGTCAATG	TTCAGGGTCAAGGCAAACTT	Sigma-Aldrich
Cyp2e1	CTTAGGGAAAACCTCCGCAC	GGGACATTCCTGTGTTCCAG	Sigma-Aldrich
Cyp7a1	CACCATTCCTGCAACCTTCTGG	ATGGCATTCCCTCCAGAGCTGA	Sigma-Aldrich
Fatp4	GACTTCTCCAGCCGTTTCCACA	CAAAGGACAGGATGCGGCTATTG	Sigma-Aldrich
Fut2	GGTGGATGATGGTGGAAGTC	TTCCCTGTTACCACAGCCAG	Sigma-Aldrich
Il1b	TGTGCAAGTGTCTGAAGCAGC	TGGAAGCAGCCCTTCATCTT	Sigma-Aldrich
Il18	GACAGCCTGTGTTCGAGGATATG	TGTTCTTACAGGAGAGGGTAGAC	Sigma-Aldrich
Lpl	GGGAGTTTGGCTCCAGAGTTT	TGTGTCTTCAGGGGTCCTTAG	Sigma-Aldrich
Muc2	AAGTTTGCCCCTGGCTATGAC	CCTTGCAGTCAAACTCAAAGT	Sigma-Aldrich
Nlrp3	TCACAACTCGCCCAAGGAGGAA	AAGAGACCACGGCAGAAGCTAG	Sigma-Aldrich
Npc1l1	ATCGCACTACCATCCAGGACCT	CCCAGAGTAGCCTTGGAATCCA	Sigma-Aldrich
Ocln	ATTTATGATGAACAGCCCCC	CATAGTCAGATGGGGGTGGA	Sigma-Aldrich
Reg3b	GGCTTCATTCTTGTCCTCCA	TCCACCTCCATTGGGTTCT	Sigma-Aldrich
Reg3g	AAGCTTCCTTCCTGTCCTCC	TCCACCTCTGTTGGGTTCAT	Sigma-Aldrich
S100a8	AAATCACCATGCCCTCTACAAG	CCCACTTTTATCACCATCGCAA	Sigma-Aldrich
Srebf1	CGACTACATCCGCTTCTTGCAG	CCTCCATAGACACATCTGTGCC	Sigma-Aldrich
Srebf2	AGAAAGAGCGGTGGAGTCCTTG	GAACTGCTGGAGAATGGTGAGG	Sigma-Aldrich
Trem2	CTACCAGTGTCAGAGTCTCCGA	CCTCGAAACTCGATGACTCCTC	Sigma-Aldrich
Zo-1	AGCTGTTTCCTCCATTGCTG	GAGATGTTTATGCGGACGG	Sigma-Aldrich

1.5 Biological samples

Description	Source	Identifier
Mouse plasma	Ldlr-/- mice	NA
Mouse liver tissue	Ldlr-/- mice	NA
Mouse aorta and heart tissue	Ldlr-/- mice	NA
Mouse fecal samples	Ldlr-/- mice	NA
Mouse intestinal tissue	Ldlr-/- mice	NA

1.6 Deposited data

Name of repository	Identifier	Link
NA		

1.7 Software

Software name	Manufacturer	Version
GraphPad Prism	GraphPad Software	v10
FlowJo	Tree Star Inc.	v10
ImageJ	NIH	V4
Adobe Photoshop Elements	Adobe	2021
R	GNU	v4.3.3
DESeq2	Bioconductor	v1.40.2
clusterProfiler	Bioconductor	v4.8.1
GSVA	Bioconductor	v1.50.5
MetaboAnalystR	MetaboAnalyst	
PeakBot	open source	v0.9.54
DADA2	Bioconductor	
CellSens Dimension	Olympus	v4.1
ZEN	Carl Zeiss	v2.3 Pro

1.8 Other (*e.g.* drugs, proteins, vectors etc.)

Item	Details
High-fat high-cholesterol diet	TD88137, ssniff Spezialdiäten GmbH
Antibiotics cocktail	metronidazole, ampicillin, vancomycin
Collagenase I	LifeTech
Collagenase XI	Sigma-Aldrich
DNase I	LifeTech
Hyaluronidase	Sigma-Aldrich
Oil Red O	Sigma-Aldrich
Sirius Red F3B	Sigma-Aldrich
Liquid DAB substrate	Dako
Citrate Buffer, PH 6.0, 10X	Sigma-Aldrich
Hematoxylin	AppliChem
Donkey serum	Sigma-Aldrich (cat. D9663)

1.9 Please provide the details of the corresponding methods author for the manuscript:

NA

2.0 Please confirm for randomised controlled trials all versions of the clinical protocol are included in the submission. These will be published online as supplementary information.

NA

JHEP Reports

Created : November, 2018
