# Supplementary material for: Alcohol intake reprograms hepatic immune-metabolic circuits to exacerbate murine atherosclerosis and human cardiovascular risk
Source: JHEP Rep. 2026 Jun 19;8(9):101932. doi: 10.1016/j.jhepr.2026.101932 (PMC13482647; doi:10.1016/j.jhepr.2026.101932)
Supplement: Supplementary file 4 [file mmc4.pdf]

Research article

Alcohol intake reprograms hepatic immune-metabolic circuits to exacerbate murine atherosclerosis and human cardiovascular risk

Authors

Constanze Hoebinger, Georg Semmler, Oleksandr Petrenko, …, Christian Datz, Christoph J. Binder, Tim Hendrikx

Correspondence

tim.hendrikx@meduniwien.ac.at (T. Hendrikx).

Graphical abstract

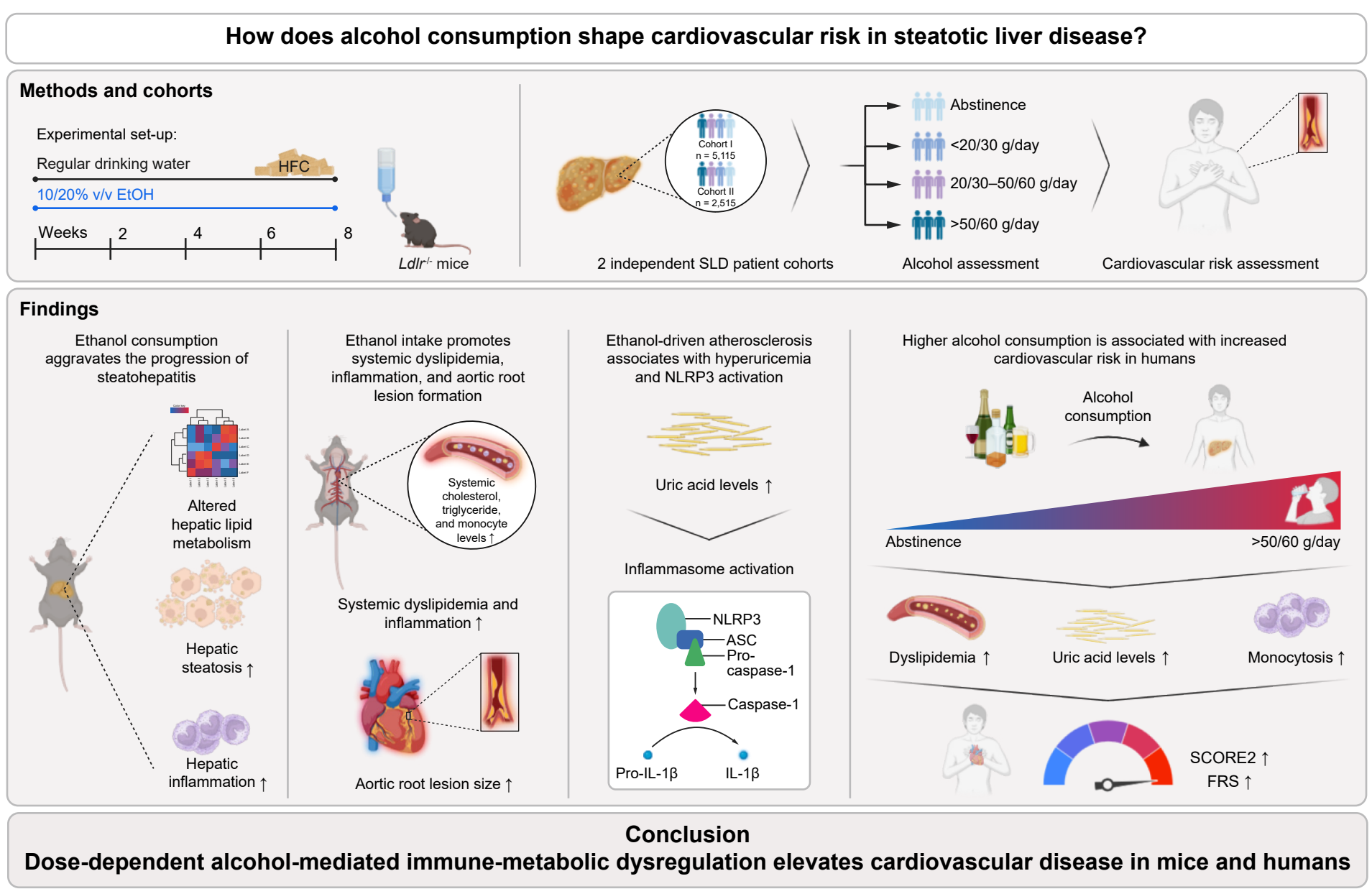

Highlights:

- Ethanol plus HFC feeding in *Ldlr*$^{-/-}$ mice accelerates early atherosclerosis.
- In HFC-fed *Ldlr*$^{-/-}$ mice, ethanol exacerbates hepatic and systemic dyslipidemia and induces pro-atherogenic monocytosis.
- Ethanol induces hyperuricemia and NLRP3 inflammasome signaling in HFC-fed *Ldlr*$^{-/-}$ mice, revealing a targetable pathway.
- Individuals with SLD show concordant, dose-dependent associations between alcohol intake and cardiometabolic risk.

Impact and implications:

Alcohol consumption, even at moderate levels, contributes to cardiovascular risk, particularly in individuals with steatotic liver disease (SLD). Combining murine models with human cohort data, we show that alcohol intake in SLD is associated with coordinated immune-metabolic alterations, including dyslipidemia, monocytosis, hyperuricemia, and enhanced NLRP3 inflammasome signaling, collectively indicating an elevated cardiovascular risk profile. These findings are relevant to hepatology and cardiovascular medicine, linking alcohol consumption to measurable systemic pathways beyond liver injury. Clinically, systematic assessment of alcohol intake and incorporation into cardiovascular risk evaluation may improve risk stratification and support closer surveillance and preventive strategies in patients with SLD.

https://doi.org/10.1016/j.jhepr.2026.101932

© 2026 The Author(s). Published by Elsevier B.V. on behalf of European Association for the Study of the Liver (EASL). This is an open access article under the CC BY license (http://creativecommons.org/licenses/by/4.0/). JHEP Reports, 2026, 8, 1–15

Alcohol intake reprograms hepatic immune-metabolic circuits to exacerbate murine atherosclerosis and human cardiovascular risk

Constanze Hoebinger[1], Georg Semmler[2,3,4,5], Oleksandr Petrenko[1,6], Dragana Rajcic[1], Dorothea Katharina Hoffelner[1], Tereza Duckova[1], Taras Baranovskyi[1], Laura Goederle[1], My Phung Khuu[7], Christoph Reinhardt[7,8], Petra Pjevac[9,10], Joana Séneca[1,9,10], Kamil Mieczkowski[1], Katharina Burger[11], Ina Bergheim[11], Sophie Gensluckner[12], Andreas Völkerer[13], Thomas Reiberger[2,3], Bernhard Scheiner[2,3], Elmar Aigner[12], Bernhard Wernly[12,13], Christian Datz[13], Christoph J. Binder[1], Tim Hendrikx[1,*]

JHEP Reports **2026**. vol. 8 | 1–15

Check for updates

Background & Aims: Recent reclassification of steatotic liver disease (SLD) distinguishes metabolic dysfunction-associated steatotic liver disease (MASLD) from MetALD, a newly defined entity combining MASLD with alcohol consumption. Since the mechanisms linking alcohol consumption in the context of SLD to cardiovascular disease (CVD) – the leading cause of SLD mortality – remain elusive, we investigated how metabolic dysregulation and alcohol intake synergistically promote atherosclerosis.

Methods: Low-density lipoprotein receptor-deficient (*Ldlr*$^{-/-}$) mice were fed a high-fat, high-cholesterol (HFC) diet with regular or ethanol-containing drinking water (10–20% v/v). Germ-free and antibiotic-treated *Ldlr*$^{-/-}$ mice were used to assess the contribution of ethanol-induced dysbiosis. Associations between alcohol consumption and cardiometabolic risk were assessed in two human cohorts (n = 5,115, n = 2,515).

Results: Ethanol intake in HFC diet-fed *Ldlr*$^{-/-}$ mice exacerbated hepatic steatosis and systemic dyslipidemia, despite only modest elevations in systemic ethanol levels (p values ≤0.05). Liver transcriptomic profiling revealed ethanol-induced alterations in lipid metabolism and enhanced proinflammatory signatures, accompanied by increased recruitment of Ly6C^{high} monocytes to the liver (p = 0.0121) and elevated levels in the circulation (p = 0.0043). Correspondingly, ethanol-consuming HFC diet-fed *Ldlr*$^{-/-}$ mice developed enlarged aortic root lesions (p = 0.0105). Neither germ-free conditions nor antibiotic treatment mitigated CVD progression. Metabolomic profiling revealed hyperuricemia in ethanol-exposed HFC diet-fed *Ldlr*$^{-/-}$ mice, which was associated with upregulation of inflammasome-related genes in the liver, along with an increase in hepatic NLRP3 protein expression (p values ≤0.05). Notably, human data mirrored these findings, demonstrating a dose-dependent association between alcohol intake, dyslipidemia, monocytosis, hyperuricemia, and increased cardiovascular risk (p values ≤0.05).

Conclusion: Our findings identify alcohol as an important immunomodulatory lifestyle factor that contributes to elevated cardiovascular risk.

© 2026 The Author(s). Published by Elsevier B.V. on behalf of European Association for the Study of the Liver (EASL). This is an open access article under the CC BY license (http://creativecommons.org/licenses/by/4.0/).

Introduction

The updated nomenclature for steatotic liver disease (SLD) distinguishes alcohol-associated liver disease (ALD) from metabolic dysfunction-associated steatotic liver disease (MASLD), which together represent the most prevalent chronic liver diseases worldwide, affecting over one-third of the global population.[1,2] MASLD is characterized by metabolic dysfunction, including dyslipidemia, obesity, and type 2 diabetes that drive disease progression, while ALD is defined by high alcohol intake, irrespective of cardiometabolic risk factors. Nonetheless, both conditions share similar pathophysiological mechanisms and exhibit comparable histopathological features, including steatosis, steatohepatitis, fibrosis, and cirrhosis.[3] To capture their overlap, the updated classification introduced a new diagnostic category, MetALD, defined as MASLD with moderate alcohol intake (140-350 g/week for women; 210-420 g/week for men).[4,5]

The intersection of metabolic dysfunction and alcohol consumption is supported by epidemiological evidence demonstrating increased morbidity and mortality associated with alcohol intake in patients with metabolic disorders, such as MASLD.[6,7] Nonetheless, alcohol consumption remains an underestimated risk factor. Studies indicate that 74% of Europeans consume alcohol, with nearly 10% drinking daily.[8] With projections suggesting that 55% of the population may develop SLD by 2040,[9] understanding the synergistic effects

* Corresponding author. Address: Department of Laboratory Medicine, KILM, Medical University of Vienna, Vienna, Austria.
E-mail address: tim.hendrikx@meduniwien.ac.at (T. Hendrikx).
https://doi.org/10.1016/j.jhepr.2026.101932

between metabolic dysfunction and alcohol exposure, as seen in MetALD, is essential.[10,11]

Metabolic dysregulation creates a pro-atherogenic milieu characterized by dyslipidemia, inflammation, oxidative stress, insulin resistance, and endothelial dysfunction, rendering cardiovascular diseases (CVDs) the leading cause of mortality among patients with MASLD.[12] Increased *de novo* lipogenesis, a hallmark of MASLD, elevates saturated fatty acid levels and contributes to dyslipidemia, characterized by elevated plasma cholesterol, triglycerides, and low-density lipoprotein cholesterol, together with reduced high-density lipoprotein cholesterol.[13] Hepatic lipid accumulation further triggers inflammatory and oxidative cascades that accelerate atherosclerosis.[14] In ALD, excessive ethanol consumption can further exacerbate dyslipidemia and thereby augment atherogenesis.[15–17] However, the effects of alcohol consumption on CVD in MetALD and the underlying mechanisms remain unclear, underscoring the need to investigate how alcohol contributes to cardiovascular risk in these patients.[11,18]

In this study, we demonstrate that ethanol consumption combined with a high-fat, high-cholesterol (HFC) diet in *Ldlr*$^{-/-}$ mice, which display a human-like lipoprotein profile, induces systemic dyslipidemia, inflammation, hyperuricemia, and accelerates the progression of steatohepatitis. These metabolic alterations subsequently accelerate atherosclerosis development and plaque progression. Our findings indicate that disease aggravation may be linked to elevated uric acid levels and NLRP3 inflammasome-related signaling, independent of ethanol-mediated effects on the intestinal microbiota. Notably, we observed similar alterations in systemic lipid levels and inflammatory markers upon alcohol consumption in humans. These findings identify individuals with underlying SLD who consume alcohol as having an increased risk of experiencing cardiovascular events.

Materials and methods

Murine models

Female *Ldlr*$^{-/-}$ mice were used to model SLD and atherosclerosis. Accordingly, *Ldlr*$^{-/-}$ mice were fed a HFC diet for 8 weeks and received either regular drinking water or ethanol-supplemented water, with concentrations alternating between 10% (v/v) for 3 days and 20% (v/v) for 4 days, provided *ad libitum* for 8 or 16 weeks. To assess microbiome-dependent effects, additional experiments were performed using broad-spectrum antibiotic treatment as well as germ-free and conventionally raised *Ldlr*$^{-/-}$ mice. Blood, liver, intestine, stool, aorta, and heart were collected for analyses. Further information can be found in the supplementary materials and methods.

Human cohorts

Two independent human cohorts were analyzed. Cohort I included 5,115 individuals aged 40-69 years undergoing first screening colonoscopy within the prospective SAKKOPI registry, and Cohort II comprised 2,515 first-time referrals to a hepatology outpatient clinic. In both cohorts, self-reported alcohol intake was converted to daily ethanol consumption and used to stratify participants into abstainers and low (<20 g/day for women, <30 g/day for men), moderate (20–50 g/day for women, 30–60 g/day for men), or high (>50 g/day for women, >60 g/day for men) alcohol consumption groups. Patients with liver diseases other than SLD were excluded. Cardiovascular risk was assessed using SCORE2 and the Framingham risk score. Laboratory parameters were compared across alcohol consumption groups using appropriate statistical tests. Subgroup analyses excluding statin users were additionally performed (see supplementary materials and methods for further information).

Biochemical analyses and statistics

Murine plasma and tissues were analyzed using biochemical assays, ELISA, quantitative PCR, western blotting, microscopy, (immuno)histochemistry, and flow cytometry to assess metabolic, inflammatory, and vascular parameters. Biochemical assays included the quantification of plasma and hepatic lipids (cholesterol, triglycerides, free fatty acids), as well as uric acid, ethanol, endotoxin (lipopolysaccharide), cytokines, and immunoglobulins. Atherosclerotic lesion burden was assessed in the aortic root and by *en face* analysis of the aorta. In addition, liver transcriptomics, plasma metabolomics, and fecal 16S rRNA sequencing were performed to characterize ethanol-associated immune-metabolic alterations. Additional details are provided in the supplementary materials and methods.

Results

Ethanol feeding aggravates systemic dyslipidemia and hepatic steatosis by altering lipid metabolism in the liver of *Ldlr*$^{-/-}$ mice

To study the impact of alcohol consumption on CVD progression, *Ldlr*$^{-/-}$ mice were fed a HFC diet for 8 weeks and received either regular drinking water or ethanol-supplemented drinking water (Fig. 1A). Notably, ethanol intake resulted in only a modest increase in plasma ethanol levels compared with controls (Fig. 1B), thereby distinguishing our model from murine ALD, where systemic ethanol levels are reported to be up to ten-fold higher.[19] Body weight gain was comparable between groups (Fig. S1A), and hepatic expression of *Cyp2e1* and *Adh1*, two key enzymes in ethanol metabolism, was unchanged or only modestly altered in ethanol-fed mice (Fig. S1B and C).

Furthermore, ethanol intake aggravated systemic dyslipidemia in HFC-fed *Ldlr*$^{-/-}$ mice, as indicated by increased plasma cholesterol, triglycerides, and free fatty acid levels after 8 weeks (Fig. 1C–E). While lipid quantification in the small intestinal content showed no difference in cholesterol levels, triglyceride levels were increased in HFC diet-fed *Ldlr*$^{-/-}$ mice receiving ethanol compared with mice receiving regular drinking water (Fig. S1D and E). Small intestinal mRNA levels of genes involved in lipid uptake and export (*Npc1l1*, *Abcg4*, *Abcg8*, *Cd36*, *Fatp4*) remained unchanged (Fig. S1F). Yet, HFC diet-fed *Ldlr*$^{-/-}$ mice drinking ethanol displayed increased liver-to-body weight ratio, accompanied by elevated cholesterol and triglyceride content in the liver, paralleled by histological evidence of increased macrosteatosis (Figs 1F-J and S1G).

Hepatic bulk RNA sequencing revealed distinct transcriptional profiles between ethanol-exposed and control mice (Fig. S1H). Under the defined thresholds, the two experimental

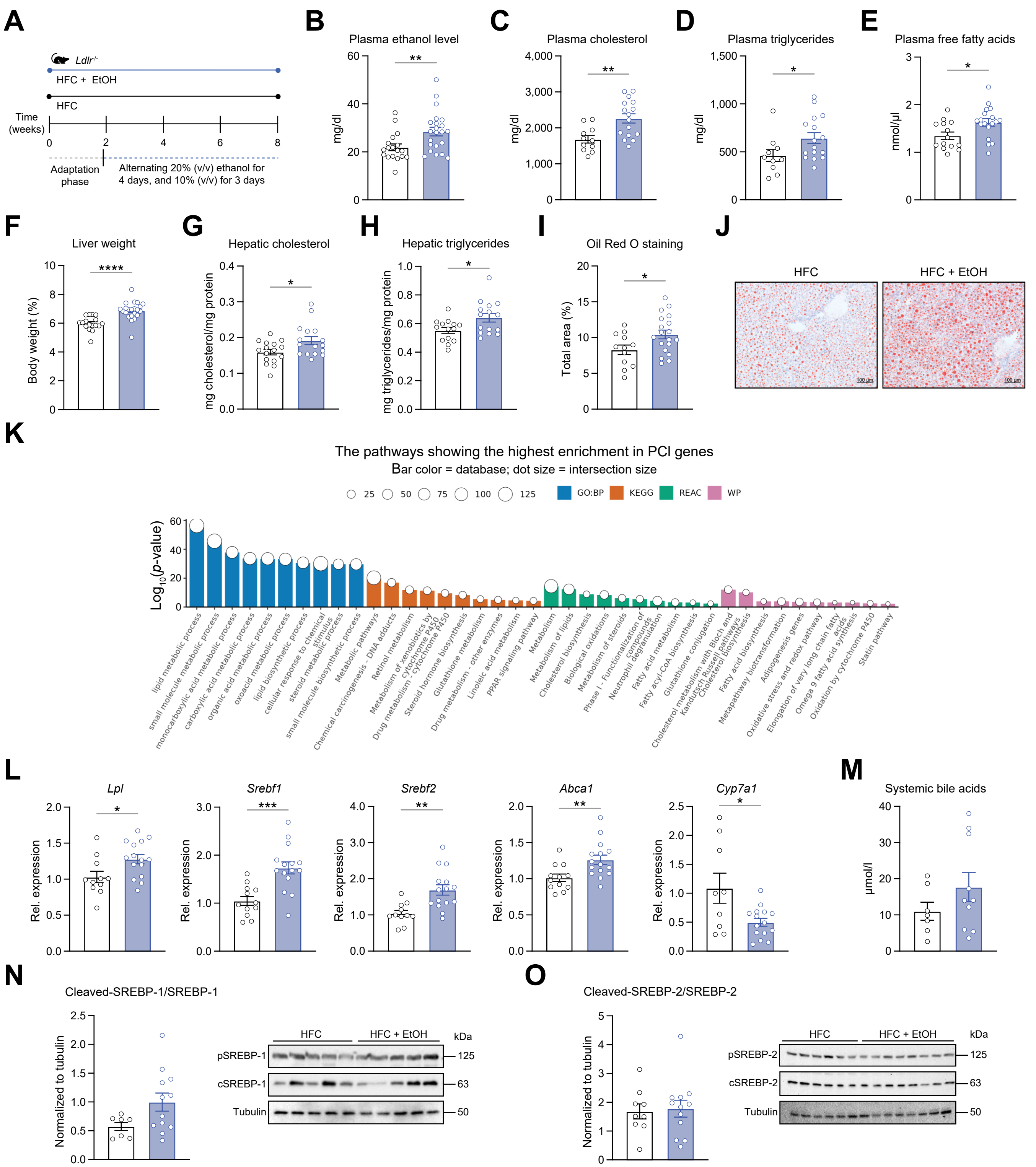

Fig. 1. Ethanol intake exacerbates dyslipidemia and alters hepatic lipid metabolism in *Ldlr$^{-/-}$* mice fed a HFC diet. (A) Study design. (B) Plasma ethanol levels. (C-E) Plasma lipid levels. (F) Liver weight. (G-H) Hepatic lipid levels. (I) Quantification and (J) representative images of Oil Red O staining of liver sections. (K) Significant pathways showing the highest enrichment score (up to the top 10) with their respective significance values. Colors indicate database source, and bubble size the degree of overlap between the annotated pathway and the query gene set. (L) Hepatic gene expression. (M) Systemic bile acid levels. (N-O) Cleaved SREBP-1 and SREBP-2 protein levels. Data are mean ± SEM (n = 4–18 for the HFC group; n = 6–22 for the HFC+EtOH group). mRNA was normalized to *18S* and expressed relative to HFC-fed *Ldlr$^{-/-}$* mice receiving regular water. Statistical significance was determined using a two-tailed, unpaired Student's *t* test. *$p \leq 0.05$, **$p \leq 0.01$, ****$p \leq 0.0001$. EtOH, ethanol; HFC, high-fat, high-cholesterol.

groups had 219 differentially expressed genes (Fig. S1I), of which a selection was confirmed by qPCR analyses (Fig. 1L). The 500 genes with the largest absolute loadings on principal component 1, which captured most of the variance between groups, were significantly enriched for pathways related to lipid, cholesterol, and fatty acid turnover, suggesting that these transcriptional changes contribute to increased hepatic steatosis upon ethanol intake in HFC-fed *Ldlr*$^{-/-}$ mice (Fig. 1K).

Targeted per-gene analyses of cholesterol-related signatures revealed that *Apoa4*, *Pcsk9*, *Soat1, and Lipa* exhibited the most pronounced expression differences, with higher expression in HFC-fed *Ldlr*$^{-/-}$ mice receiving ethanol (Fig. S1J and K). While genes such as *Soat1* and *Lipa* were increased, suggesting enhanced intracellular cholesterol esterification and turnover, genes involved in cholesterol clearance were decreased in HFC-fed *Ldlr*$^{-/-}$ mice subjected to ethanol feeding. In particular, *Lcat*, which esterifies free cholesterol into cholesteryl esters in HDL particles to promote reverse cholesterol transport, and *Cyp7a1*, the rate-limiting enzyme for bile acid synthesis, were markedly downregulated (Figs 1L and S1J,K). Yet, total circulating bile acid levels remained unchanged (Fig. 1M). Despite elevated transcriptional levels, mean hepatic cleaved SREBP-1 levels were only mildly elevated upon alcohol intake (Fig. 1N), while cleaved SREBP-2 was comparable between groups (Fig. 1O). These results indicate selective activation of the lipogenic SREBP-1 pathway in HFC-fed *Ldlr*$^{-/-}$ mice upon ethanol intake, suggesting that ethanol primarily alters cholesterol clearance rather than enhancing cholesterol synthesis. Collectively, these findings imply that specific alterations in hepatic lipid homeostasis promote enhanced hepatic steatosis and systemic hyperlipidemia in ethanol-exposed HFC-fed *Ldlr*$^{-/-}$ mice.

HFC diet in combination with ethanol is associated with accelerated hepatic inflammation in *Ldlr*$^{-/-}$ mice

Immune profiling of the liver transcriptome dataset uncovered an altered cytokine signature in mice drinking ethanol (Figs 2A and S2A). *Il16*, *Il33*, and *Il1a* were among the most differentially upregulated cytokines in *Ldlr*$^{-/-}$ mice receiving HFC diet and ethanol. Moreover, we observed increased expression of immune cell markers associated with macrophages and monocytes (*Cd68*, *Cd38*), cytotoxic T/NK cells (*Prf1*, *Tigit*, *Nkg7*), neutrophils (*S100a8, S100a9*), and B cells (*Cd22, Cd79a, Blnk, Bank1*) (Figs 2B and S2B). Conversely, markers characteristic of T helper 1 cells and regulatory T cells (*Ifng*, *Cxcr3*, *Il2ra*, *Ahr*) were suppressed (Figs 2B and S2B). Reactome pathway analysis further indicated that signatures of neutrophil degranulation, FcγR-dependent phagocytosis, and IL-10 signaling, among others, prevailed in mice with ethanol intake compared with controls (Fig. S2C).

Consistent with bulk RNA-seq data, gene expression of the monocyte-recruiting chemokine *Ccl2* and the macrophage-associated marker *Trem2* were elevated in the livers of HFC diet-fed *Ldlr*$^{-/-}$ mice exposed to ethanol intake (Fig. 2C). These transcriptional changes were accompanied by a relative increase in infiltrating monocytes, particularly the proinflammatory Ly6C^{high} subset (Fig. 2D), whereas resident macrophages were modestly reduced (Fig. 2E,F). In line with signatures of neutrophil degranulation, mRNA levels of *Cxcl2*, a neutrophil-attracting chemokine, and *S100a8,* an alarmin predominantly produced by activated neutrophils and monocytes, were increased (Fig. 2C). Although relative hepatic neutrophil abundance was unchanged (Fig. 2G), absolute numbers of Ly6G^{+} neutrophils were elevated in ethanol-exposed mice (Fig. 2H). No difference in the relative amount of T cells was found in the liver (Fig. 2D), while hepatic B cells showed a relative decrease in HFC-fed *Ldlr*$^{-/-}$ mice subjected to ethanol (Fig. 2D). Yet, circulating B cell numbers and plasma IgM, IgG1, or IgA levels remained unchanged (Figs S2D–F and S3C).

Evaluation of hepatic fibrosis revealed increased expression of profibrotic genes (*Col1a1*, *Col3a1*) in the liver (Fig. 2I), while collagen deposition was slightly higher in ethanol-fed *Ldlr*$^{-/-}$ mice (Fig. 2J). Overall, these data indicate that in *Ldlr*$^{-/-}$ mice, ethanol exposure combined with HFC diet feeding particularly aggravates the development of steatohepatitis.

Ethanol intake in HFC diet-fed *Ldlr*$^{-/-}$ mice exacerbates systemic inflammation and aortic lesion formation

Characterization of systemic effects of ethanol in combination with a HFC diet showed that ethanol administration resulted in elevated total monocytes and an increased monocyte-to-lymphocyte ratio, a parameter proposed to predict the severity of coronary artery disease (Table 1).[20] Within the monocyte compartment, systemic proinflammatory Ly6C^{high} monocytes were specifically elevated in ethanol-supplemented mice (Figs 3A and S3A). Ethanol intake was further associated with elevated red blood cell counts, hemoglobin, and hematocrit, while mean corpuscular hemoglobin was reduced. Although total white blood cell counts were not significantly affected, relative lymphocyte abundance was decreased, while granulocytes and eosinophils were increased (Table 1). Strikingly, other hematological parameters and systemic immune cell composition did not show any significant changes (Table 1, Fig. S3B–E).

Of note, ethanol administration markedly increased the aortic root lesion size in HFC diet-fed *Ldlr*$^{-/-}$ mice compared with HFC diet-fed *Ldlr*$^{-/-}$ mice receiving regular drinking water (Figs 3B and S3F), accompanied by only a modest increase in necrotic core size (Fig. 3C). Despite elevated monocytosis, the relative macrophage content within the cellular area of aortic root lesions was found to be comparable between the groups (Fig. 3D). Furthermore, collagen content within atherosclerotic lesions did not differ between the study groups (Fig. 3E). To determine whether accelerated atherosclerosis was attributable to systemic hypercholesterolemia, we compared lesion size in mice with matched cholesterol exposure and found that ethanol remained associated with larger lesions, indicating that ethanol-driven atherogenesis is at least partly independent of systemic cholesterol (Fig. S3G and H). Bold values indicate statistically significant differences with a p value $\leq$ 0.05.

After 16 weeks of dietary intervention (Fig. 3F), and consistent with findings from the 8-week setting, systemic ethanol levels were only modestly increased and body weight gain remained comparable between groups (Fig. S3I and J), yet hepatic steatosis progression was increased in HFC-fed *Ldlr*$^{-/-}$ mice subjected to ethanol feeding (Fig. S3K-O). Notably, although plasma cholesterol levels were similar after 16 weeks of dietary intervention, circulating triglyceride levels were elevated in HFC diet-fed *Ldlr*$^{-/-}$ mice upon ethanol intake

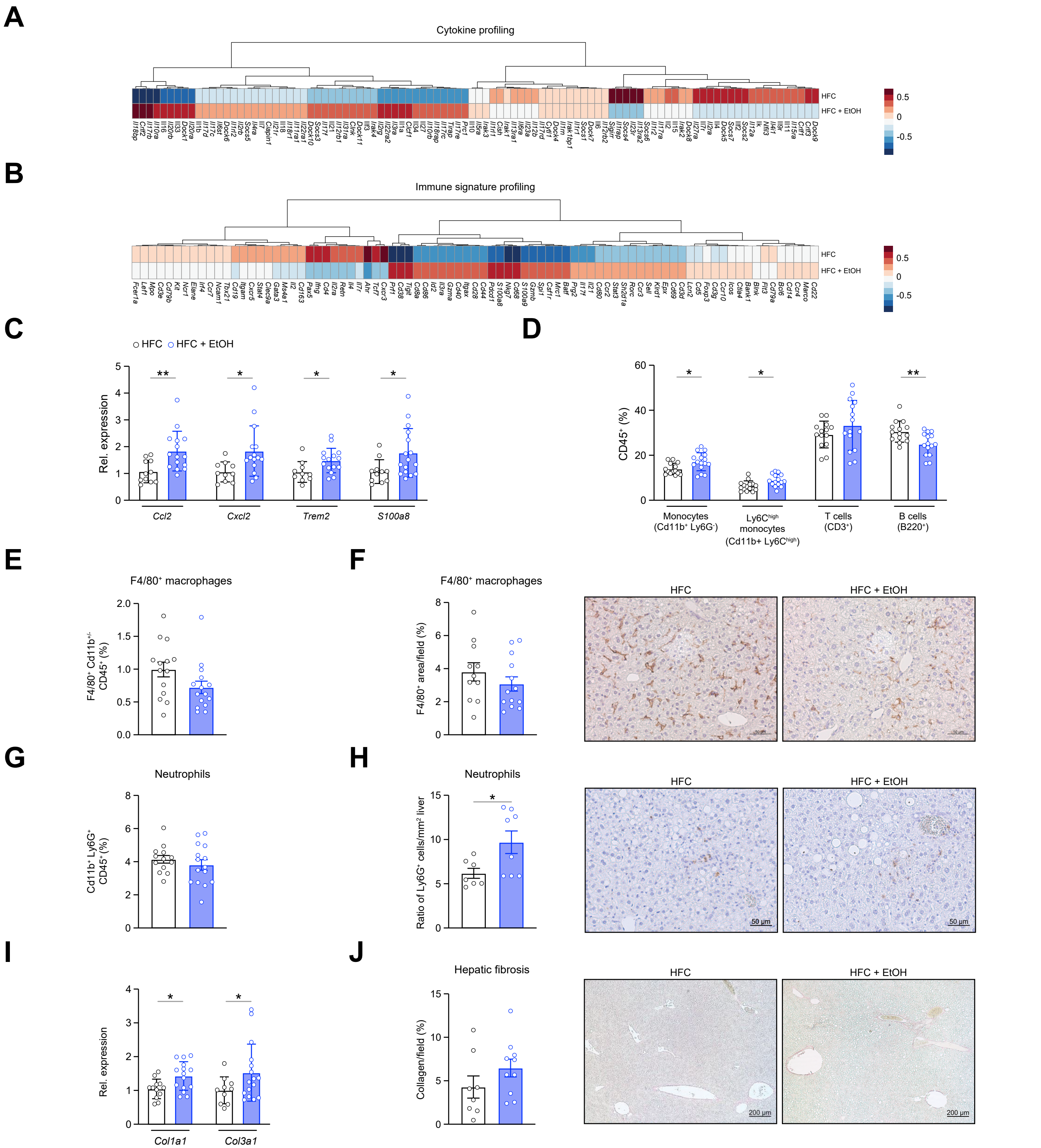

Fig. 2. *Ldlr*$^{-/-}$ mice exhibit accelerated hepatic inflammation following HFC diet combined with ethanol intake. (A) Heatmap showing the expression levels of cytokine genes from the BioMart database and (B) of immune-related genes in livers of HFC-fed *Ldlr*$^{-/-}$ mice with regular and ethanol-containing drinking water. Abundance values are scaled and averaged per group. (C) Hepatic gene expression. (D) Quantification of hepatic immune cell populations by flow cytometry. (E) Quantification of hepatic Kupffer cells by flow cytometry. (F) Quantification and representative images of F4/80-stained liver sections. (G) Quantification of hepatic neutrophils by flow cytometry. (H) Quantification and representative images of Ly6G-stained liver sections. (I) Hepatic gene expression. (J) Quantification and representative images of Sirius Red-stained liver sections. Data are mean ± SEM (n = 7–14 for the HFC group and n = 8–16 for the HFC+EtOH group). mRNA was normalized to *18S* and expressed relative to HFC-fed *Ldlr*$^{-/-}$ mice on regular water. Statistical significance was determined using a two-tailed unpaired Student's *t* test; **p* ≤0.05, ***p* ≤0.01. EtOH, ethanol; HFC, high-fat, high-cholesterol.

Table 1. Murine blood parameters after 8-week dietary intervention.

Parameter	HFC	HFC + EtOH	*p* values
White blood cells, 10^3/μl	9.5 (2.5)	8.6 (2.1)	0.09126
Lymphocytes, 10^3/μl	7.5 (2.2)	6 (1.3)	0.0712
Lymphocytes, %	79.9 (2.7)	71.2 (4.7)	**0.001**
Monocytes, 10^3/μl	0.5 (0.3)	0.6 (0.2)	0.2351
Monocytes, %	5.8 (0.8)	7.0 (1.1)	**0.0113**
Granulocytes, 10^3/μl	1.4 (0.3)	2.0 (0.9)	0.0744
Granulocytes, %	14.3 (2.9)	21.4 (5.1)	**0.0022**
Eosinophils, %	1.3 (0.2)	1.8 (0.6)	**0.0282**
Red blood cells, 10^6/μl	16.4 (2.6)	12.9 (3.4)	**0.0211**
Hemoglobin, g/dl	23.3 (3.3)	18.8 (5)	**0.0298**
Hematocrit, %	83.0 (11.8)	67.2 (17.7)	**0.0337**
Mean corpuscular volume, μm^3	50.7 (1.1)	52 (1.3)	**0.0287**
Mean corpuscular hemoglobin, pg	13.6 (0.9)	12.5 (0.5)	**0.0135**
Mean corpuscular hemoglobin concentration, d/dl	26.8 (1.5)	27.9 (1.0)	0.3853
Red blood cell distribution width, %	14.54 (0.9)	14.6 (0.8)	0.2083
Platelets, 10^3/μl	981.6 (105.7)	838.5 (193.3)	0.0627
Mean platelet volume, μm^3	5.9 (0.2)	6.0 (0.2)	0.0627
Monocyte/lymphocyte, ratio	0.09 (0.1)	0.09 (0.02)	**0.0181**

EtOH, ethanol; HFC, high-fat, high-cholesterol.
Statistical significance was determined using two-tailed, unpaired Student's *t* tests.

(Fig. 3G,H). No major changes in hepatic immune cell composition or proinflammatory gene expression in the liver were observed between the groups (Fig. S3P,Q). Yet, ethanol intake in HFC diet-fed *Ldlr*$^{-/-}$ mice resulted in an increase in circulating proinflammatory Ly6C^{high} monocytes (Fig. 3I, Table 2), despite no differences between groups in atherosclerotic lesion area, necrotic core size, or relative macrophage or collagen content within aortic root lesions (Fig. 3J-M). In summary, these findings suggest that ethanol intake significantly accelerates early atherosclerosis development, whereas this effect appears attenuated at later stages following prolonged exposure.

Accelerated cardiometabolic features occur independently of ethanol-induced dysbiosis

Mounting evidence suggests that ethanol intake alters microbiome composition and intestinal barrier function, both of which are associated with the progression of SLD and CVD.[21–25] Accordingly, 16S rRNA gene amplicon sequencing of fecal samples from the 8-week dietary intervention revealed distinct clustering between ethanol-exposed and control mice (Fig. S4A and B). At the genus level, genera within the family *Tannerellaceae* and genus *Akkermansia*, both reported to exert protective effects in liver disease and atherosclerosis development,[26,27] showed the largest decrease in relative abundance in ethanol-consuming mice, while *Muribaculaceae* displayed a markedly increased relative abundance (Fig. S4A). Importantly, these changes in fecal microbiome composition did not appear to affect intestinal barrier integrity, as indicated by comparable mRNA and protein levels of tight junction genes (Fig. S4C and E), as well as mucus barrier genes across the groups (Fig. S4D). Consistently, systemic lipopolysaccharide levels were comparable between HFC diet-fed *Ldlr*$^{-/-}$ mice receiving regular and ethanol-containing drinking water (Fig. S4F).

To assess the functional contribution of ethanol-induced dysbiosis to steatohepatitis and atherosclerosis, we compared germ-free (GF) and conventionally raised (CONV-R) *Ldlr*$^{-/-}$ mice after 8 weeks of HFC diet feeding with ethanol supplementation (Fig. 4A). While body weight and plasma ethanol levels were comparable between GF and CONV-R *Ldlr*$^{-/-}$ mice receiving ethanol (Fig. S4G and H), hepatic steatosis was increased in GF mice (Fig. 4B,C). In contrast, hepatic immune cell populations and proinflammatory gene expression profiles did not differ between groups (Figs 4D-H and S4I). Systemic dyslipidemia and circulatory Ly6C^{high} monocytes remained unaffected by the absence of the microbiome in GF *Ldlr*$^{-/-}$ mice (Fig. 4I-K). Most notably, quantification of atherosclerotic lesions revealed comparable plaque areas between CONV-R and GF *Ldlr*$^{-/-}$ mice receiving HFC diet combined with ethanol-supplemented drinking water (Fig. 4L).

In a complementary approach, *Ldlr*$^{-/-}$ mice were fed a HFC diet with either regular or ethanol-containing drinking water for 4 weeks in combination with broad-spectrum antibiotic treatment to deplete the intestinal microbiome (Fig. 4M), a duration sufficient to induce increased systemic dyslipidemia and elevated circulating Ly6C^{high} monocytes upon ethanol intake (Fig. S4J-L). Microbiota ablation was verified by the absence of bacterial growth on agar plates inoculated with cecum contents (Fig. S4M). While ethanol intake in the setting of antibiotic treatment did not affect body weight, plasma ethanol levels were higher in mice receiving ethanol (Fig. S4N and O). Supporting our findings in GF settings, ethanol-fed mice displayed elevated plasma cholesterol and triglyceride levels, as well as increased circulating Ly6C^{high} monocytes despite microbiome depletion (Fig. 4N-P). Collectively, these data indicate that ethanol intake amplifies systemic dyslipidemia, inflammation and aortic root lesion formation in *Ldlr*$^{-/-}$ mice on a HFC diet, independently of ethanol-induced gastrointestinal dysbiosis.

Accelerated atherosclerosis in mice drinking ethanol correlates with exacerbated hyperuricemia and NLRP3 inflammasome activation

Untargeted plasma metabolite profiling revealed a clear separation between experimental groups based on systemic metabolomic profiles (Fig. S5A), providing insight into ethanol-driven metabolic alterations in the context of SLD. The most

Table 2. Characteristics of 4,334 individuals in the general population (Cohort I) excluding statin users (n = 781, 15.3%), stratified by self-reported alcohol intake.

Parameter	Overall (N = 4,334)	Abstinence (n = 1,595)	<20/30 g/day (n = 2,284)	20/30-50/60 g/day (n = 418)	>50/60 g/day (n = 37)	*p* values
Age, years	58.7 (8.25)	59.5 (8.58)	57.9 (7.95)	59.7 (8.11)	60.3 (8.70)	**<0.001**
Male sex	2,199 (50.7%)	479 (30.0%)	1,434 (62.8%)	258 (61.7%)	28 (75.7%)	**<0.001**
BMI, kg/m^2	26.9 (4.60)	27.4 (5.11)	26.7 (4.28)	26.4 (3.94)	28.2 (4.97)	**<0.001**
BMI ≥30 kg/m^2	932 (21.5%)	428 (26.8%)	423 (18.5%)	70 (16.7%)	11 (29.7%)	**<0.001**
Abdominal obesity	3,145 (72.6%)	1,214 (76.1%)	1,593 (69.7%)	308 (73.7%)	30 (81.1%)	**<0.001**
Alcohol, g/week	20.0 [0-100]	0 [0-0]	80.0 [20.0-100]	280 [160-280]	500 [440-560]	**<0.001**
Diabetes	586 (13.5%)	242 (15.2%)	268 (11.7%)	68 (16.3%)	8 (21.6%)	**0.005**
Uric acid, mg/dl	5.70 (1.51)	5.39 (1.44)	5.81 (1.49)	6.11 (1.56)	6.90 (2.35)	**<0.001**
AST, U/L	20.0 [17.0-25.0]	20.0 [17.0-24.0]	21.0 [17.0-25.0]	22.0 [18.0-27.0]	22.0 [20.0-29.0]	**<0.001**
AST xULN	0.486 [0.400-0.600]	0.514 [0.429-0.629]	0.480 [0.400-0.600]	0.520 [0.420-0.629]	0.486 [0.420-0.600]	**<0.001**
ALT, U/L	20.0 [15.0-29.0]	19.0 [14.0-26.0]	21.0 [16.0-29.0]	23.0 [16.0-32.0]	23.0 [17.0-30.0]	**<0.001**
ALT xULN	0.486 [0.371-0.660]	0.486 [0.371-0.680]	0.480 [0.371-0.657]	0.514 [0.380-0.720]	0.514 [0.360-0.620]	0.128
GGT, U/L	25.0 [17.0-42.0]	22.0 [15.0-33.0]	27.0 [18.0-43.0]	33.0 [21.0-58.0]	53.0 [32.0-99.0]	**<0.001**
GGT xULN	0.500 [0.350-0.800]	0.475 [0.350-0.725]	0.517 [0.367-0.800]	0.633 [0.433-1.07]	0.917 [0.617-1.65]	**<0.001**
Glucose, mg/dl	99.4 (18.1)	99.7 (19.7)	98.9 (16.6)	101 (18.6)	105 (21.3)	**0.017**
HbA1c (%)	5.53 (0.496)	5.59 (0.555)	5.48 (0.436)	5.52 (0.491)	5.57 (0.956)	**<0.001**
Cholesterol, mg/dl	227 (40.9)	226 (41.9)	227 (39.5)	234 (42.6)	233 (51.9)	**0.004**
HDL cholesterol, mg/dl	59.4 (16.8)	58.0 (15.6)	59.7 (16.9)	61.9 (19.3)	64.4 (20.3)	**<0.001**
LDL cholesterol, mg/dl	148 (37.3)	149 (37.5)	147 (36.4)	151 (39.3)	150 (50.7)	0.325
Triglycerides, mg/dl	104 [77.0-145]	104 [77.0-143]	103 [76.0-145]	114 [82.0-158]	117 [85.5-166]	**0.006**
SCORE2, points[1]	4.8 [2.9-7.3]	4.3 [2.6-6.6]	4.9 [3.0-7.4]	5.8 [4.0-9.3]	7.1 [4.9-8.6]	**<0.001**
FRS, points[2]	0.132 [0.0738-0.229]	0.115 [0.0658-0.208]	0.134 [0.0766-0.237]	0.165 [0.0925-0.278]	0.178 [0.122-0.277]	**<0.001**
CRP, mg/dl	0.180 [0.100-0.360]	0.200 [0.100-0.400]	0.160 [0.100-0.300]	0.160 [0.100-0.300]	0.200 [0.100-0.500]	**<0.001**
Monocytes, %	7.20 [6.00-8.70]	6.80 [5.70-8.40]	7.40 [6.20-8.90]	7.60 [6.65-8.70]	7.05 [6.08-8.35]	**<0.001**
Monocytes, G/L	0.410 [0.330-0.510]	0.400 [0.320-0.490]	0.420 [0.330-0.520]	0.410 [0.355-0.545]	0.330 [0.310-0.403]	**0.045**
Monocyte to lymphocyte ratio	0.241 [0.190-0.313]	0.231 [0.190-0.303]	0.247 [0.187-0.319]	0.249 [0.207-0.309]	0.244 [0.184-0.303]	0.207

ALT, alanine aminotransferase; AST, aspartate aminotransferase; CRP, C-reactive protein; FRS, Framingham risk score; GGT, gamma-glutamyltransferase; ULN, upper limit of normal. Bold values indicate statistically significant differences with a *p* value ≤0.05.

[1]Only calculated in 2,397 patients aged 40-69, without diabetes and without established cardiovascular disease.

[2]Only calculated in patients without established cardiovascular disease. Group comparisons used Kruskal–Wallis tests for continuous variables and chi-square tests for categorical variables; for normally-distributed variables (uric acid and total cholesterol), one-way ANOVA was used.

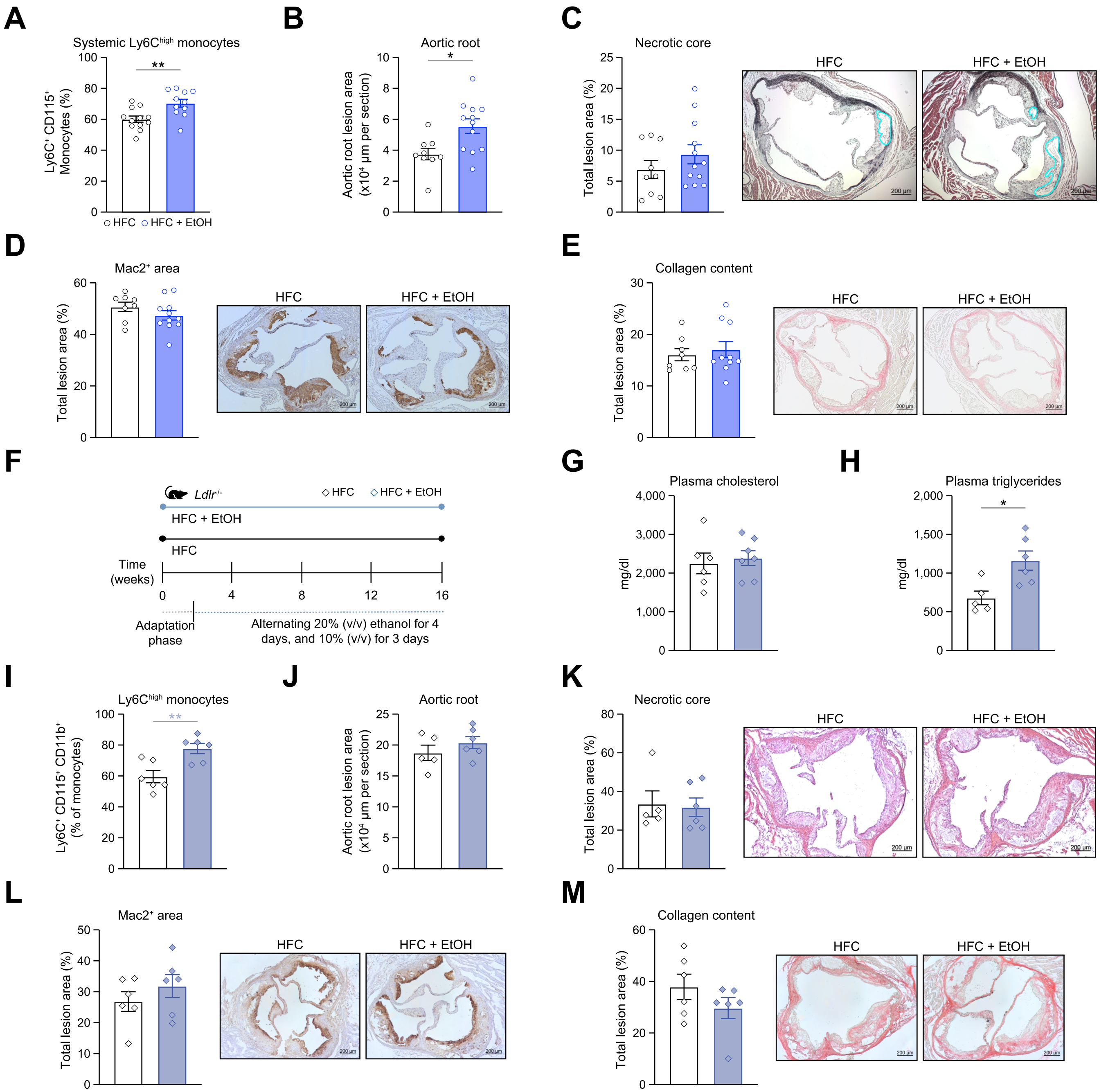

Fig. 3. HFC diet with ethanol intake results in increased atherosclerosis in *Ldlr*$^{-/-}$ mice. (A-H) 8-week dietary intervention. (A) Quantification of circulating Ly6C^{high} monocytes determined by flow cytometry. (B) Average aortic root lesion size expressed as µm^2 per section. (C) Relative size of necrotic core expressed as a percentage of total plaque area and representative images of Masson's trichrome-stained aortic root sections. (D) Quantification and representative images of infiltrating macrophages assessed by Mac2$^+$ staining of aortic root sections. (E) Quantification and representative images of collagen content assessed by Sirius Red staining of aortic root sections. (F-M) 16 weeks dietary intervention. (F) Study design. (G-H) Plasma lipid levels. (I) Quantification of circulating Ly6C^{high} monocytes determined by flow cytometry. (J) Quantification of aortic root lesion size, calculated as the average lesion size (µm^2) from seven sections throughout the aortic root. (K) Quantification of relative size of necrotic core expressed as a percentage of total plaque area and representative images of H&E-stained aortic root sections. (L) Quantification and representative images of infiltrating macrophages assessed by Mac2$^+$ staining of aortic root sections. (M) Quantification and representative images of collagen content assessed by Sirius Red staining of aortic root sections. Data in (A-E) are mean ± SEM (n = 6–12 for the HFC group and n = 10–12 for the HFC+EtOH group). Data in (F–M) are mean ± SEM (n = 5–6 for the HFC group and n = 5–6 for the HFC+EtOH group). Statistical significance was determined using a two-tailed unpaired Student's *t* test; *p ≤0.05, **p ≤0.01. EtOH, ethanol; HFC, high-fat, high-cholesterol.

upregulated metabolite driving this separation in HFC diet-fed *Ldlr*$^{-/-}$ mice receiving ethanol was dihydroxy-isovalerate, together with isoleucine, indicating alterations in branched-chain amino acid metabolism (Figs 5A and S5B). Notably, functional annotation of the differentially regulated metabolites revealed higher levels of uric acid, allantoin, and xanthosine, all key components of purine metabolism, upon ethanol intake (Figs 5A and S5C). Metabolic set enrichment analysis further

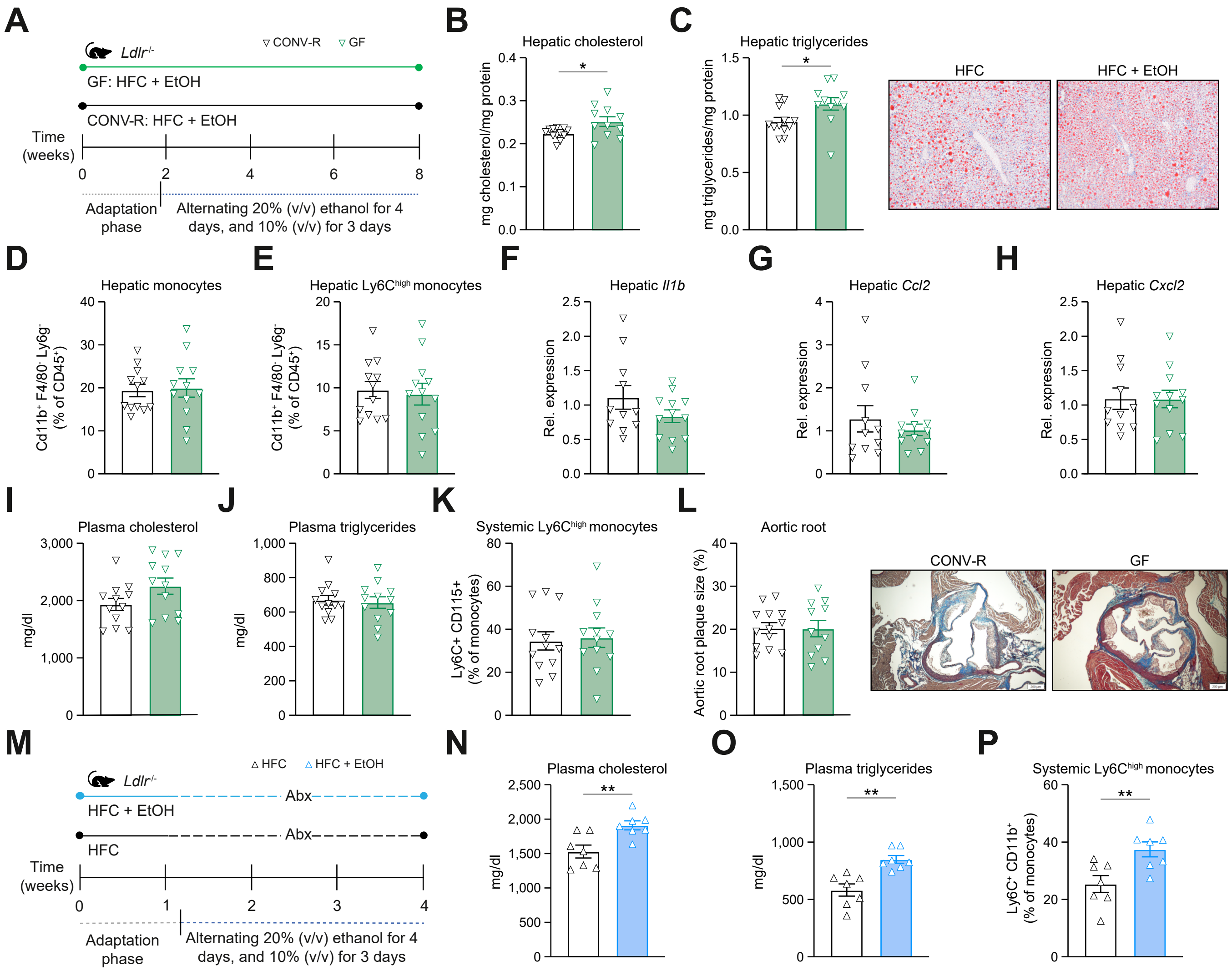

Fig. 4. Exacerbated atherosclerosis occurs independent of ethanol-induced dysbiosis. (A-L) 8-week dietary intervention with CONV-R and GF *Ldlr*$^{-/-}$ mice. (A) Schematic of the 8-week dietary intervention study in CONV-R and GF *Ldlr*$^{-/-}$ mice. (B) Hepatic cholesterol and (C) triglyceride levels and representative images of Oil Red O staining of liver sections. (D) Quantification of hepatic monocytes and (E) Ly6C^{high} assessed by flow cytometry. (F-H) Hepatic gene expression. (I-J) Plasma lipid levels. (K) Quantification of circulating Ly6C^{high} monocytes assessed by flow cytometry. (L) Quantification of aortic root lesion size, expressed as the percentage lesion area relative to total aortic valve area and representative images of Masson's Trichrome-stained aortic root sections. (M-P) 4-week dietary intervention with antibiotic-induced microbiome depletion. (M) Schematic of the 4-week dietary intervention study in *Ldlr*$^{-/-}$ mice, combined with a 3-week antibiotic-induced microbiome depletion. (N-O) Plasma lipid levels. (P) Quantification of circulating Ly6C^{high} monocytes assessed by flow cytometry. Data in (A-L) are mean ± SEM (n = 11–12 for the CONV-R group and n = 11–12 for the GF group). Data in (M–P) are presented as mean ± SEM (n = 7 for the HFC group and n = 7 for the HFC+EtOH group). Statistical significance was determined using a two-tailed unpaired Student's *t* test; **p* ≤0.05, ***p* ≤0.01. CONV-R, conventionally raised; EtOH, ethanol; GF, germ-free; HFC, high-fat, high-cholesterol.

identified "Transport of inorganic cations/anions and amino acids/oligopeptides," "SLC transporter disorders," and "Amino-acid metabolism" among metabolites with higher abundance, whereas those with lower abundance were enriched in "Ornithine aminotransferase deficiency," "HHH-syndrome," and related ornithine-arginine pathways (Fig. 5B). These enrichment patterns were concordant with the purine metabolite accumulation. Consistently, uric acid concentrations were elevated in both plasma and liver of mice consuming ethanol (Fig. 5C,D).

These findings align with previous studies documenting an association between hyperuricemia and alcohol consumption in humans.[28] Moreover, uric acid has been shown to activate the NLRP3 inflammasome, thereby contributing to disease progression.[29] To assess inflammasome activation in our model, we analyzed transcriptomic signatures for genes involved in inflammasome pathways. Notably, expression of *Nlrp3*, *Casp1*, *Aim2*, *Il1b*, and *Il18* was upregulated in HFC diet-fed *Ldlr*$^{-/-}$ mice subjected to ethanol (Figs 5E and S5D), accompanied by an increased inflammasome pathway activity score (Fig. 5F). The inflammasome-related transcriptional changes (Fig. 5G) were corroborated by increased IL-1β concentrations and higher NLRP3 levels in the livers of ethanol- and HFC diet-fed *Ldlr*$^{-/-}$ mice (Fig. 5H,I). Importantly, ethanol intake also resulted in increased expression of inflammasome-related genes in the aortic root of HFC diet-

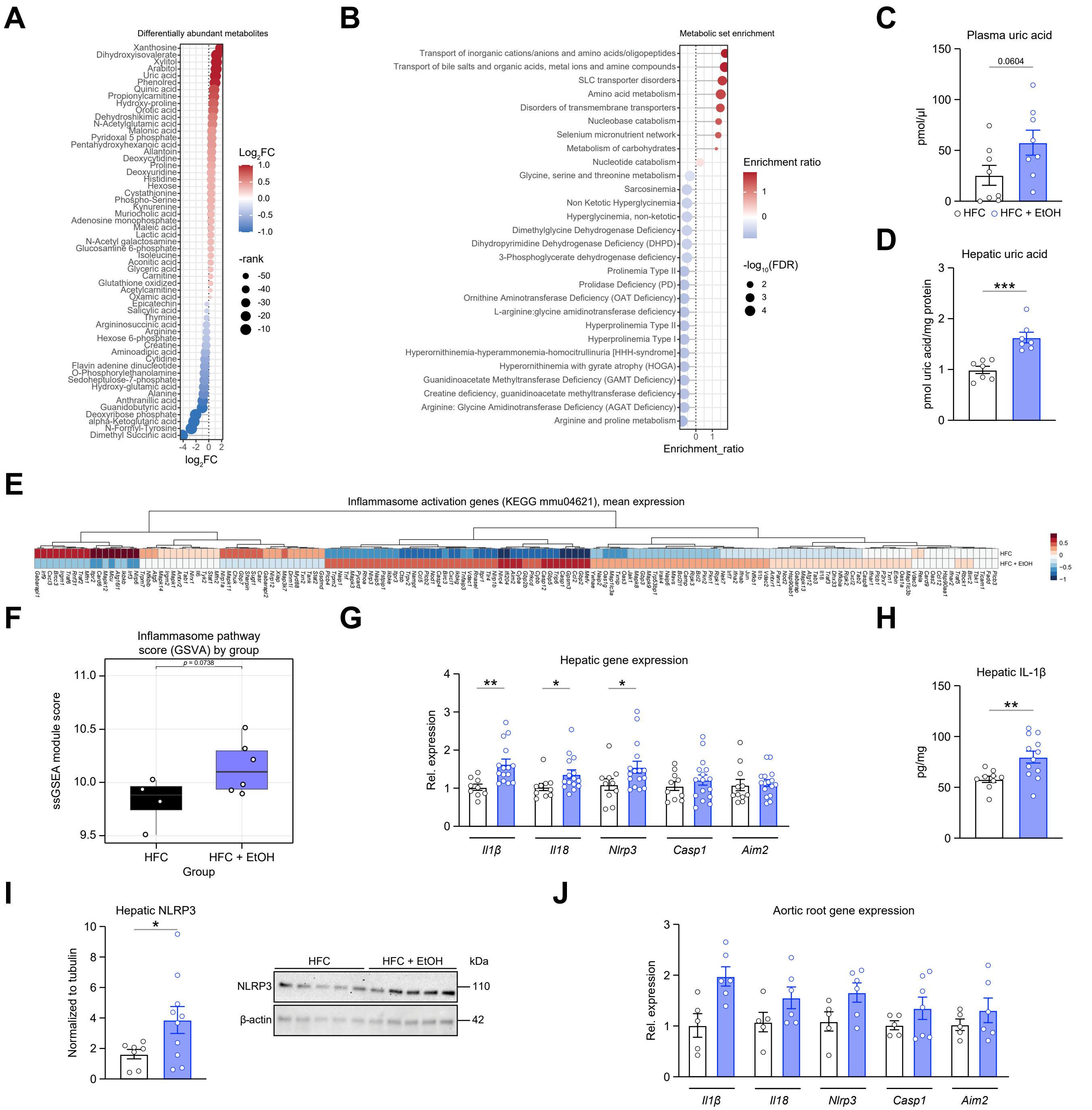

Fig. 5. Ethanol intake results in accelerated hyperuricemia and NLRP3 inflammasome activation. (A) Differentially abundant systemic metabolites (n = 8 for the HFC group and n = 9 for the HFC+EtOH group). The color scale indicates the direction of regulation, and dot size is inversely correlated with the rank of a metabolite in the list. (B) Metabolic pathway enrichment based on plasma metabolomics data (n = 8 for the HFC group and n = 8 for the HFC+EtOH group). The color scale indicates the direction of regulation. The enrichment scores are the result of over-representation testing against the MetaboAnalyst pathways universe. (C) Plasma and (D) hepatic uric acid levels. (E) Heatmap showing the expression of genes involved in inflammasome activation (KEGG pathway accession mmu04621) in livers of mice depicted in Fig. 1A. Average values for each group are presented. (F) Inflammasome activation pathway score based on DEG in liver bulk RNA sequencing. (G) Hepatic gene expression. (H) Hepatic IL-1β protein concentration assessment. (I) Hepatic NLRP3 protein levels. (J) Aortic root gene expression. Data are mean ± SEM (n = 5–11 for the HFC group; n = 6–16 for the HFC+EtOH group). mRNA was normalized to *18S* and expressed relative to HFC-fed *Ldlr*$^{-/-}$ mice on regular drinking water. Statistical significance was determined using a two-tailed unpaired Student's *t* tests; ***p* ≤0.01, ****p* ≤0.001. DEG, differentially expressed genes; EtOH, ethanol; HFC, high-fat, high-cholesterol.

fed *Ldlr*$^{-/-}$ mice (Fig. 5J). Collectively, these findings support a potential role for uric acid-driven NLRP3 inflammasome activation in promoting atherosclerotic disease progression in mice drinking ethanol.

Alcohol consumption in humans is associated with elevated cholesterol, uric acid, and monocyte numbers in blood, contributing to higher cardiovascular risk

To interrogate these relationships in a human setting, we analyzed a population-based cohort (Cohort I; n = 5,115; Fig. S6A). After exclusion of statin users, individuals were stratified as abstinent or according to alcohol intake (<20/30 g/day, 20/30–50/60 g/day, and >50/60 g/day). Higher alcohol consumption was associated with progressive increases in systemic levels of gamma-glutamyltransferase, alanine aminotransferase, and aspartate aminotransferase, along with a dose-dependent elevation in cholesterol and triglyceride levels, consistent with our observations in mice (Table 2, Fig. 6A). Lipid-related associations remained robust upon inclusion of statin-treated individuals (Table S2). In line with our murine findings, besides elevated C-reactive protein, alcohol intake resulted in elevated monocyte counts in the blood (Table 2, Fig. 6B). Additionally, uric acid, one of the metabolites identified as elevated in our murine metabolomic profiling and previously reported to be increased in patients with cardiovascular disease, exhibited a dose-dependent increase in circulating levels with increasing alcohol intake[14,30,31] (Table 2, Fig. 6C). Consequently, alcohol consumption was associated with a dose-dependent increase in cardiovascular risk, as assessed by SCORE2 and the Framingham risk score (Table 2, Fig. 6D,E). A subgroup analysis of 1,916 individuals with ultrasound-confirmed hepatic steatosis showed consistent results after stratification by alcohol intake, independent of statin use (Table S3).

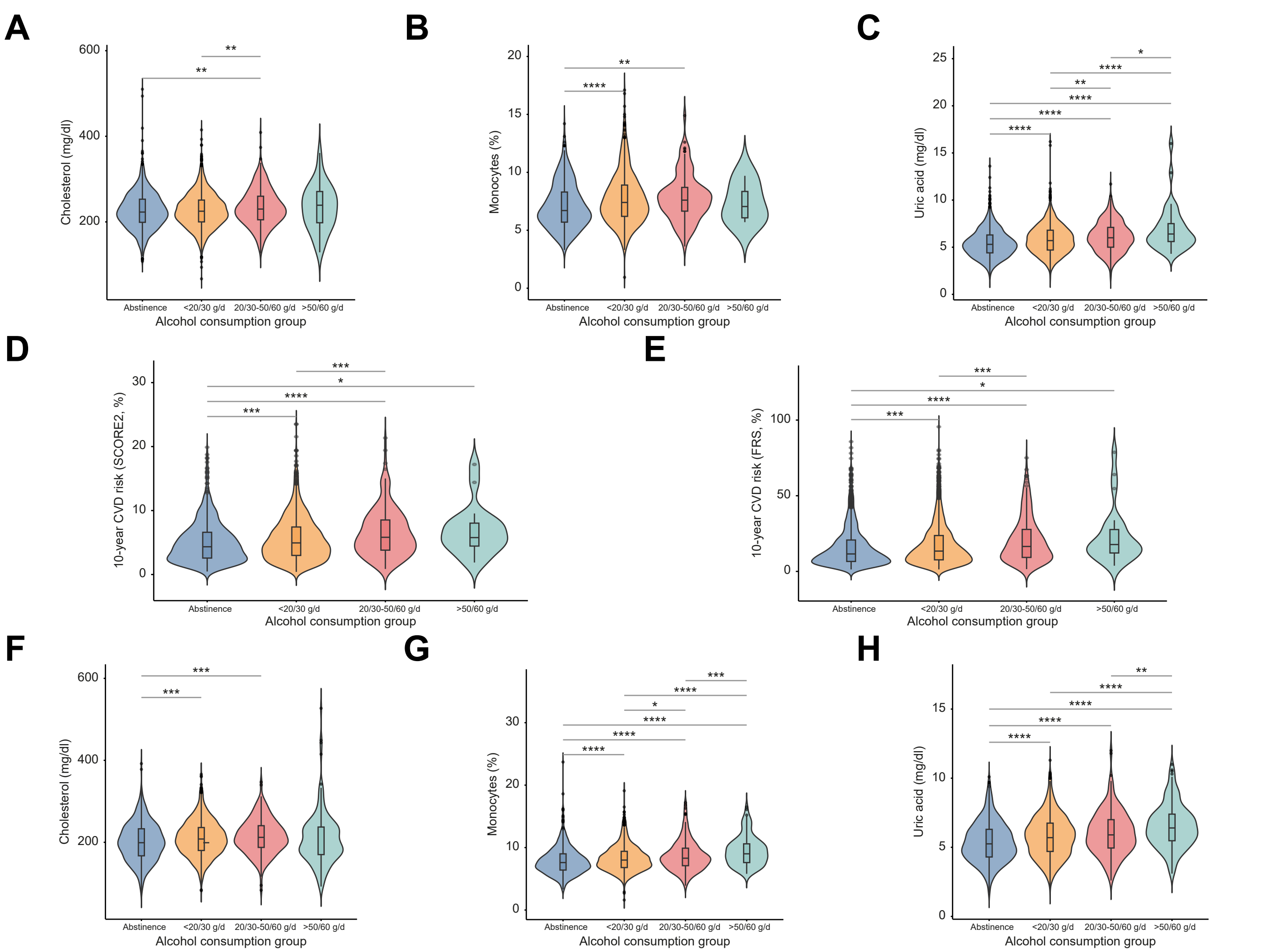

Fig. 6. Increased alcohol consumption associates with enhanced systemic dyslipidemia, higher uric acid, elevated monocyte counts and increased cardiovascular risk scores. (A) Systemic cholesterol levels, (B) relative monocyte counts, (C) uric acid levels, (D) SCORE2 risk score, and (E) Framingham risk score in 4,334 individuals of the general population, stratified by alcohol consumption: abstinent, <20/30 g/day, 20/30–50/60 g/day, or >50/60 g/day (Cohort I; excluding statin users). (F) Systemic cholesterol levels, (G) relative monocyte counts, and (H) uric acid levels in 2,166 individuals from a hepatology outpatient clinic, stratified by alcohol consumption: abstinent, <20/30 g/day, 20/30–50/60 g/day, or >50/60 g/day (Cohort II; excluding statin users). Graphs showing violin plots integrated with boxplots of key metabolic parameters in Cohort I (A-E), and Cohort II (F-H). Brackets and asterisks indicate significant differences in post-hoc comparisons applying either Tukey's (cholesterol, uric acid) or Dunn's (Monocytes, SCORE2, Framingham risk score) *post hoc* comparisons, as applicable. *p ≤0.05, **p ≤0.01, p ≤0.001 and ****p ≤0.0001.

Table 3. Characteristics of 2,166 individuals referred to a hepatology outpatient clinic (Cohort II) excluding statin users (n = 349, 13.9%), stratified by self-reported alcohol intake.

Parameter	Overall (N = 2,166)	Abstinence (n = 636)	<20/30 g/day (n = 1,115)	20/30-50/60 g/day (n = 243)	>50/60 g/day (n = 172)	*p* values
Age, years	50.8 (15.0)	49.6 (15.8)	49.8 (14.8)	54.7 (14.3)	55.8 (12.0)	**<0.001**
Male sex	1,226 (56.6%)	245 (38.5%)	675 (60.5%)	158 (65.0%)	148 (86.0%)	**<0.001**
BMI, kg/m^2	27.1 (7.94)	27.3 (5.56)	27.1 (9.83)	26.8 (4.18)	26.6 (4.66)	0.738
BMI ≥30 kg/m^2	502 (23.2%)	182 (28.6%)	245 (22.0%)	44 (18.1%)	31 (18.0%)	**<0.001**
Diabetes	251 (11.6%)	87 (13.7%)	115 (10.3%)	20 (8.2%)	29 (16.9%)	**0.013**
Uric acid, mg/dl	5.75 (1.52)	5.37 (1.43)	5.78 (1.49)	6.03 (1.55)	6.52 (1.56)	**<0.001**
AST, U/L	31.0 [24.0-44.0]	29.0 [23.0-42.0]	30.0 [23.0-41.0]	32.0 [24.5-46.0]	44.5 [32.0-76.5]	**<0.001**
ALT, U/L	39.0 [25.0-63.0]	37.0 [24.0-64.0]	39.0 [25.0-63.0]	41.0 [27.0-63.5]	44.5 [30.8-63.0]	**<0.001**
GGT, U/L	59.0 [29.0-128]	49.0 [23.0-113]	56.0 [27.0-112]	74.0 [38.0-154]	190 [70.8-444]	**<0.001**
Glucose, mg/dl	98.5 (24.2)	99.1 (27.8)	96.9 (22.0)	100 (22.9)	104 (23.9)	**0.002**
HbA1c (%)	5.43 (0.683)	5.53 (0.844)	5.41 (0.598)	5.37 (0.530)	5.29 (0.688)	**<0.001**
Cholesterol, mg/dl	208 (45.9)	201 (46.4)	210 (42.7)	214 (42.1)	208 (63.1)	**<0.001**
HDL cholesterol, mg/dl	58.4 (19.1)	57.5 (18.4)	58.1 (18.7)	61.3 (19.7)	60.0 (22.5)	**0.034**
LDL cholesterol, mg/dl	133 (40.5)	128 (40.1)	135 (38.1)	137 (39.0)	128 (54.8)	**<0.001**
Triglycerides, mg/dl	111 [78.0-162]	107 [78.0-154]	112 [78.0-168]	116 [79.5-154]	117 [89.0-184]	0.059
CRP, mg/dl	0.170 [0.100-0.390]	0.180 [0.100-0.413]	0.150 [0.100-0.360]	0.150 [0.100-0.400]	0.200 [0.100-0.543]	**0.009**
Monocytes, G/L	0.500 [0.400-0.630]	0.480 [0.388-0.620]	0.480 [0.390-0.610]	0.520 [0.410-0.640]	0.615 [0.490-0.763]	**<0.001**
Monocytes, %	8.0 [6.8-9.4]	7.6 [6.4-9.0]	8.0 [6.8-9.4]	8.3 [7.1-9.9]	9.0 [7.6-10.6]	**<0.001**
Monocyte to lymphocyte ratio	0.273 [0.215-0.360]	0.255 [0.204-0.343]	0.271 [0.214-0.347]	0.292 [0.232-0.385]	0.353 [0.265-0.482]	**<0.001**

ALT, alanine aminotransferase; AST, aspartate aminotransferase; CRP, C-reactive protein; FRS, Framingham risk score; GGT, gamma-glutamyltransferase.
Group comparisons used Kruskal–Wallis tests for continuous variables and chi-square tests for categorical variables; for normally-distributed variables (uric acid and total cholesterol), one-way ANOVA was used. Bold values indicate statistically significant differences with a *p* value ≤0.05.

T- hese findings were independently recapitulated in patients referred to a hepatology outpatient clinic (Cohort II; n = 2,515; Fig. S6B), in which higher alcohol intake was again associated with increased circulating cholesterol, monocyte counts, and uric acid levels (Table 3, Fig. 6F-H), including in patients with confirmed hepatic steatosis (controlled attenuation parameter ≥248 dB/m) (Table S5). Overall, these findings provide translational evidence that higher alcohol intake not only aggravates liver disease progression but also promotes systemic dyslipidemia, monocytosis, and an increase in uric acid levels, thereby contributing to an increased cardiovascular risk.

Discussion

SLD is the leading cause of chronic liver disease worldwide and its prevalence continues to rise due to changes in lifestyle factors, such as the adoption of a Western diet, sedentary lifestyles, and harmful alcohol consumption.[1] Given the high global prevalence of SLD and the frequent coexistence of metabolic dysfunction and alcohol consumption, the term MetALD has been introduced.[4,10] Although cardiovascular diseases are known to be the leading cause of mortality in patients with SLD, the contribution of alcohol consumption to atherosclerosis remains to be elucidated.[32,33]

In this study, we describe a preclinical mouse model of SLD in the setting of alcohol consumption and show that combined HFC diet and ethanol intake synergistically promote immune-metabolic disturbances, closely recapitulating early metabolic and transcriptomic characteristics of human SLD. Importantly, our results indicate that ethanol-fed hyperlipidemic mice exhibit accelerated atherosclerosis development, and that the systemic alterations in lipid metabolism, inflammation, and hyperuricemia, which are associated with increased cardiovascular risk, are also observed in humans. Of note, our data suggest that disease aggravation may be driven by hyperuricemia, which potentially activates the NLRP3 inflammasome and occurs independently of microbiome composition.

Due to the recent introduction of the term MetALD, murine models that distinguish MetALD from MASLD and ALD are still lacking. Here, we describe a mouse model that recapitulates key hepatic features of human MetALD while also enabling investigation of its cardiovascular consequences. A retrospective study analyzing 39 murine MASLD models demonstrated that Western-style diets closely mimic the progression of human MASLD from early steatosis and inflammation to metabolic dysfunction-associated steatohepatitis with fibrosis, making these diets suitable for studying MetALD by additionally exposing mice to ethanol.[34] Despite the common practice of combining high-caloric meals with moderate, regular alcohol consumption, to our knowledge, only one murine MetALD model has been introduced so far.[35] In contrast to the model of Babuta and colleagues, which employed a 12-week dietary intervention with 58 kcal% fat, 1.0% cholesterol, supplemental sucrose and fructose, and periodic binge ethanol exposure resulting in advanced fibrosis, we used a diet containing 21% fat and 0.21% cholesterol over a period of 8 weeks. Our dietary regimen recapitulates early MASLD characteristics, including hepatic steatosis and onset of inflammation, while Babuta and colleagues' model mimics progression to accelerated fibrosis.[35] Furthermore, mice in our study exhibited plasma ethanol levels of around 30 mg/dl, compared with 115 mg/dl

reported by Babuta and colleagues.[35] Although the relatively low plasma ethanol levels in our model indicate simulation of moderate alcohol consumption, their physiological relevance is limited, as measurements were obtained at a single time point at euthanasia. Consequently, they do not capture the time course of ethanol consumption, thereby constraining conclusions regarding systemic ethanol exposure. Moreover, the lack of quantification of alcohol intake in g/kg/day represents an additional limitation of this study. However, the human-like lipoprotein profile of *Ldlr*$^{-/-}$ mice, together with their susceptibility to atherosclerosis, enhances the translational relevance of our model for investigating alcohol-induced immunometabolic dysregulation and the resulting cardiovascular risk, as well as for exploring novel therapeutic strategies in a preclinical model.[36]

Although it is well established that both a Western diet and alcohol consumption independently modulate the gastrointestinal microbiome and contribute to non-communicable diseases such as SLD and atherosclerosis, their combined impact on microbial composition and disease progression has not been thoroughly studied.[21,22,24,25] In our model, ethanol intake resulted in a reduced abundance of gut microbes such as *Akkermansia muciniphila*, a bacterium associated with metabolic health, while *Streptococcus* species, linked to subclinical atherosclerosis, were enriched.[27,37] However, depletion of the gut microbiota did not alter disease severity. These findings suggest that specific microbial taxa or their metabolites, rather than global community structure, may drive disease progression and represent potential therapeutic targets.

Interestingly, between-group differences in aortic root lesion area were attenuated at 16 weeks. Although plaque composition was evaluated by quantifying collagen and relative macrophage content, no significant differences were detected, likely due to the limited sample size. Nevertheless, ethanol-supplemented HFC-fed *Ldlr*$^{-/-}$ mice showed a modest increase in Mac2-positive and a minor reduction in Sirius Red-positive area within aortic root lesions. Therefore, one may speculate that this pattern reflects a shift toward a more proinflammatory, collagen-poor plaque phenotype associated with reduced stability. Alternatively, prolonged HFC feeding may mask ethanol-specific effects at later disease stages, highlighting the importance of early disease interception to mitigate accelerated cardiovascular risk.

Mechanistically, we observed an ethanol-mediated increase in circulating monocytes and uric acid levels in both mice and humans, accompanied by upregulation of NLRP3 inflammasome-related gene and protein expression in mice, suggesting their involvement in disease progression. Ly6C^{high} monocytes, expressing *Ccr2* and *Cx3cr1*, preferentially infiltrate artery walls and atherosclerotic plaques, where they differentiate into M1 macrophages and form foam cells through modified lipoprotein uptake, thereby amplifying local inflammation and tissue damage.[38,39] Previous studies have also shown that hyperuricemia correlates with atherosclerosis progression and systemic dyslipidemia, and that soluble uric acid promotes inflammation and atherogenesis through AMPK-mediated activation of the NLRP3 inflammasome.[30,31] Further, uric acid has been shown to regulate hepatic steatosis development through NLRP3 inflammasome-dependent mechanisms.[29] Importantly, xanthine oxidase inhibitors have shown promise in reducing hyperuricemia and atherosclerosis burden.[40] Collectively, uric acid may serve as a potential biomarker that, together with established risk markers and cardiovascular risk scores, provides a more comprehensive assessment of cardiovascular risk, while uric acid-lowering therapy could represent a strategy to help prevent or manage atherosclerosis in individuals with MetALD. However, to delineate the functional link between alcohol consumption, monocytosis, hyperuricemia, and NLRP3 inflammasome activation, and their collective contribution to cardiovascular risk, targeted modulation of purine metabolism, inhibition of NLRP3 activation, and suppression of monocyte-driven inflammation will be essential.

The Framingham risk score and SCORE2 are multivariable algorithms estimating 10-year cardiovascular risk based on parameters including sex, age, systolic blood pressure, and lipid profiles, several of which can be modulated by chronic alcohol intake. SCORE2 was specifically developed and extensively validated across European populations and is currently the European Society of Cardiology-recommended tool for cardiovascular risk estimation in primary prevention.[41,42] Accordingly, while alcohol-related modulation of individual SCORE2 components constitutes a limitation, it simultaneously underscores the close pathophysiological interconnection between alcohol exposure and established cardiometabolic risk determinants captured by these scores.

In conclusion, we demonstrate that a combination of a HFC diet with ethanol intake alters hepatic lipid metabolism, leading to sustained dyslipidemia, heightened inflammatory processes, and hyperuricemia, which is associated with inflammasome activation. Together, these factors accelerate atherosclerosis development and elevate cardiovascular risk. Our findings underscore alcohol as a long-overlooked lifestyle factor that merits consideration in managing SLD and its cardiovascular complications.

Affiliations

[1]*Department of Laboratory Medicine, KILM, Medical University of Vienna, Vienna, Austria;* [2]*Division of Gastroenterology and Hepatology, Department of Medicine III, Medical University of Vienna, Vienna, Austria;* [3]*Clinical Research Group MOTION, Medical University of Vienna, Vienna, Austria;* [4]*FLASH Centre for Liver Research, Department of Gastroenterology and Hepatology, Odense University Hospital, Odense, Denmark;* [5]*Institute of Clinical Research, Faculty of Health Sciences, University of Southern Denmark, Odense, Denmark;* [6]*Ukrainian Institute for Systems Biology and Medicine, Kyiv, Ukraine;* [7]*Center for Thrombosis and Hemostasis (CTH), University Medical Center of the Johannes Gutenberg-University Mainz, Mainz, Germany;* [8]*Research Center for Immunotherapy (FZI), University Medical Center Mainz, Johannes Gutenberg-University Mainz, Mainz, Germany;* [9]*Joint Microbiome Facility of the Medical University of Vienna and the University of Vienna, Vienna, Austria;* [10]*Division of Microbial Ecology, Centre for Microbiology and Environmental Systems Science, University of Vienna, Vienna, Austria;* [11]*Department of Nutritional Sciences, Molecular Nutritional Science, University of Vienna, Vienna, Austria;* [12]*First Department of Medicine, Paracelsus Medical University Salzburg, Salzburg, Austria;* [13]*Department of Internal Medicine, General Hospital Oberndorf, Teaching Hospital of the Paracelsus Medical University Salzburg, Oberndorf, Austria*

Abbreviations

ALD, alcohol-associated liver disease; CONV-R, conventionally raised; CVD, cardiovascular disease; GF, germ-free; HFC, high-fat, high-cholesterol; *Ldlr*$^{-/-}$, low-density lipoprotein receptor knockout (mice); MASLD, metabolic dysfunction-associated steatotic liver disease; MetALD, metabolic dysfunction-associated steatotic liver disease with moderate ethanol consumption; SLD, steatotic liver disease.

Financial support

This research was in part supported by a Zukunftskollegs grant (FWF; ZK81B), a WEAVE grant (FWF; I6853), and a Stand Alone grant (FWF; P36774) to TH. GS, TR and BS are supported by the Clinical Research Group MOTION, Medical University of Vienna, Vienna, Austria – a project funded by the Clinical Research Groups Program of the Ludwig Boltzmann Gesellschaft (Grant Nr: LBG_KFG_22_32) with funds from the Fonds Zukunft Österreich.

Conflicts of interest

GS received travel support from Amgen. SG received travel support from Roche and Ipsen. TR received grant support from AbbVie, Boehringer-Ingelheim, Gilead, MSD, Philips Healthcare, Pliant Pharmaceuticals, W.L. Gore & Associates, and Siemens, served as a speaker for AbbVie, Gilead, Gore, Intercept, Roche, MSD, and W.L. Gore & Associates, received consulting/advisory board fee from AbbVie, Bayer, Boehringer-Ingelheim, Gilead, Intercept, MSD, and Siemens, and travel support from Boehringer-Ingelheim, Gilead, and Roche. BS received travel support from AbbVie, AstraZeneca, Ipsen, and Gilead, grant support from AstraZeneca and Eisai and speaker's honoraria from AstraZeneca and Eisai. CD is part of the scientific advisory board of SPAR Österreich AG. All other authors declare no conflict of interest.

Please refer to the accompanying ICMJE disclosure forms for further details.

Authors' contributions

CH designed the study concept and experimental approaches, performed the studies, acquired, and analyzed the data, wrote, and edited the manuscript. GS provided and analyzed the human data, and provided data tables and plots, and critically revised and edited the manuscript. OP analyzed -omics data from murine studies and edited the manuscript. DR, DKH, TD, TB, KM, and KB provided technical assistance and critically revised the manuscript. LG provided technical assistance for atherosclerosis analyses. MPK performed the studies in germ-free mice, acquired and analyzed part of the data in germ-free studies, and edited the manuscript. CR provided access to germ-free mice for our studies, and critically revised and edited the manuscript. PP and JS acquired and analyzed the 16S rRNA gene amplicon sequencing data and revised the manuscript. IB critically revised the manuscript. SG, AV, TR, BS, EA, BW, CD provided access to human data, and critically revised and edited the manuscript. CJB provided part of the mice, and critically revised and edited the manuscript. TH designed the study concept and experimental approaches, performed part of the studies, and acquired part of the data, wrote and edited the manuscript, acquired funding.

Data availability

Data are available upon reasonable request to the corresponding author.

Acknowledgements

We thank the people from the Core Facility Flow Cytometry, Genomics and Imaging of the Medical University of Vienna for their support and critical help in the acquisition of our data. We thank the Life Science Compute Cluster (LiSC) of the University of Vienna for supporting the raw processing of amplicon sequencing data presented in this study. We also express our gratitude to all animal caretakers from the Medical University of Vienna responsible for animals used in this project.

Supplementary data

Supplementary data to this article can be found online at https://doi.org/10.1016/j.jhepr.2026.101932.

References

Author names in bold designate shared co-first authorship

[1] Israelsen M, Francque S, Tsochatzis EA, et al. Steatotic liver disease. Lancet 2024;404:1761–1778.
[2] **Semmler G**, **Thöne P**, Embacher J, et al. Prevalence of fibrosis and applicability of lab-based noninvasive tests from primary to tertiary care. Clin Gastroenterol Hepatol 2025. Sep 16:S1542-3565(25)00798-0.
[3] Rinella ME, Lazarus JV, Ratziu V, et al. A multisociety Delphi consensus statement on new fatty liver disease nomenclature. J Hepatol 2023;79:1542–1556.
[4] Israelsen M, Torp N, Johansen S, et al. Validation of the new nomenclature of steatotic liver disease in patients with a history of excessive alcohol intake: an analysis of data from a prospective cohort study. Lancet Gastroenterol Hepatol 2024;9:218–228.
[5] **Mandorfer M**, **Semmler G**, Aigner E, et al. Austrian multisociety consensus on metabolic dysfunction-associated steatotic liver disease : Austrian Society of Gastroenterology and Hepatology (ÖGGH), Austrian Society of Diabetology (ÖDG), Austrian Society of Obesity (ÖAG). Wien Klin Wochenschr 2025;137:307–319.
[6] Younossi ZM, Stepanova M, Ong J, et al. Effects of alcohol consumption and metabolic syndrome on mortality in patients with nonalcoholic and alcohol-related fatty liver disease. Clin Gastroenterol Hepatol 2019;17:1625–1633.e1621.
[7] **Marti-Aguado D**, **Calleja JL**, Vilar-Gomez E, et al. Low-to-moderate alcohol consumption is associated with increased fibrosis in individuals with metabolic dysfunction-associated steatotic liver disease. J Hepatol 2024;81:930–940.
[8] Eurostat. One in twelve adults in the EU consumes alcohol every day. 2021.
[9] Le MH, Yeo YH, Zou B, et al. Forecasted 2040 global prevalence of nonalcoholic fatty liver disease using hierarchical bayesian approach. Clin Mol Hepatol 2022;28:841–850.
[10] **Arab JP**, **Díaz LA**, **Rehm J**, et al. Metabolic dysfunction and alcohol-related liver disease (MetALD): position statement by an expert panel on alcohol-related liver disease. J Hepatol 2025;82:744–756.
[11] Biddinger KJ, Emdin CA, Haas ME, et al. Association of habitual alcohol intake with risk of cardiovascular disease. JAMA Netw Open 2022;5:e223849.
[12] Targher G, Byrne CD, Tilg H. MASLD: a systemic metabolic disorder with cardiovascular and malignant complications. Gut 2024;73:691–702.
[13] Berger JM, Moon YA. Increased hepatic lipogenesis elevates liver cholesterol content. Mol Cells 2021;44:116–125.
[14] Lechner K, McKenzie AL, Kränkel N, et al. High-risk atherosclerosis and metabolic phenotype: the roles of ectopic adiposity, atherogenic dyslipidemia, and inflammation. Metab Syndr Relat Disord 2020;18:176–185.
[15] **Israelsen M**, **Kim M**, Suvitaival T, et al. Comprehensive lipidomics reveals phenotypic differences in hepatic lipid turnover in ALD and NAFLD during alcohol intoxication. JHEP Rep 2021;3:100325.
[16] Hoebinger C, Rajcic D, Silva B, et al. Chronic-binge ethanol feeding aggravates systemic dyslipidemia in Ldlr(-/-) mice, thereby accelerating hepatic fibrosis. Front Endocrinol (Lausanne) 2023;14:1148827.
[17] Hoek AG, van Oort S, Mukamal KJ, et al. Alcohol consumption and cardiovascular disease risk: placing new data in context. Curr Atheroscler Rep 2022;24:51–59.
[18] **Lee HH**, **Lee HA**, Kim EJ, et al. Metabolic dysfunction-associated steatotic liver disease and risk of cardiovascular disease. Gut 2024;73:533–540.
[19] Hendrikx T, Lang S, Rajcic D, et al. Hepatic pIgR-mediated secretion of IgA limits bacterial translocation and prevents ethanol-induced liver disease in mice. Gut 2023;72:1959-1970.
[20] **Ji H**, **Li Y**, Fan Z, et al. Monocyte/lymphocyte ratio predicts the severity of coronary artery disease: a syntax score assessment. BMC Cardiovasc Disord 2017;17:90.
[21] Jonsson AL, Bäckhed F. Role of gut microbiota in atherosclerosis. Nat Rev Cardiol 2017;14:79–87.
[22] Severino A, Tohumcu E, Tamai L, et al. The microbiome-driven impact of western diet in the development of noncommunicable chronic disorders. Best Pract Res Clin Gastroenterol 2024;72:101923.
[23] Mouries J, Brescia P, Silvestri A, et al. Microbiota-driven gut vascular barrier disruption is a prerequisite for non-alcoholic steatohepatitis development. J Hepatol 2019;71:1216–1228.

[24] Bajaj JS. Alcohol, liver disease and the gut microbiota. Nat Rev Gastroenterol Hepatol 2019;16:235–246.
[25] Hsu CL, Schnabl B. The gut-liver axis and gut microbiota in health and liver disease. Nat Rev Microbiol 2023;21:719–733.
[26] **Rao Y**, **Kuang Z**, Li C, et al. Gut Akkermansia muciniphila ameliorates metabolic dysfunction-associated fatty liver disease by regulating the metabolism of L-aspartate via gut-liver axis. Gut Microbes 2021;13:1–19.
[27] Li J, Lin S, Vanhoutte PM, et al. Akkermansia muciniphila protects against atherosclerosis by preventing metabolic endotoxemia-induced inflammation in Apoe-/- mice. Circulation 2016;133:2434–2446.
[28] Gaffo AL, Roseman JM, Jacobs Jr DR, et al. Serum urate and its relationship with alcoholic beverage intake in men and women: findings from the Coronary Artery Risk Development in Young Adults (CARDIA) cohort. Ann Rheum Dis 2010;69:1965–1970.
[29] **Wan X**, **Xu C**, Lin Y, et al. Uric acid regulates hepatic steatosis and insulin resistance through the NLRP3 inflammasome-dependent mechanism. J Hepatol 2016;64:925–932.
[30] Kuwabara M, Borghi C, Cicero AFG, et al. Elevated serum uric acid increases risks for developing high LDL cholesterol and hypertriglyceridemia: a five-year cohort study in Japan. Int J Cardiol 2018;261:183–188.
[31] Kimura Y, Yanagida T, Onda A, et al. Soluble uric acid promotes atherosclerosis via AMPK (AMP-Activated protein kinase)-mediated inflammation. Arterioscler Thromb Vasc Biol 2020;40:570–582.
[32] **Chew NWS**, **Mehta A**, Goh RSJ, et al. Cardiovascular-liver-metabolic health: recommendations in screening, diagnosis, and management of metabolic dysfunction-associated steatotic liver disease in cardiovascular disease via modified delphi approach. Circulation 2025;151:98–119.
[33] **Moon JH**, **Jeong S**, Jang H, et al. Metabolic dysfunction-associated steatotic liver disease increases the risk of incident cardiovascular disease: a nationwide cohort study. EClinicalMedicine 2023;65:102292.
[34] **Vacca M**, **Kamzolas I**, **Harder LM**, et al. An unbiased ranking of murine dietary models based on their proximity to human metabolic dysfunction-associated steatotic liver disease (MASLD). Nat Metab 2024;6:1178–1196.
[35] Babuta M, Morel C, de Carvalho Ribeiro M, et al. A novel experimental model of MetALD in male mice recapitulates key features of severe alcohol-associated hepatitis. Hepatol Commun 2024;8.
[36] Gisterå A, Ketelhuth DFJ, Malin SG, et al. Animal models of atherosclerosis-supportive notes and tricks of the trade. Circ Res 2022;130:1869–1887.
[37] Emoto T, Yamashita T, Sasaki N, et al. Analysis of gut microbiota in coronary artery disease patients: a possible link between gut microbiota and coronary artery disease. J Atheroscler Thromb 2016;23:908–921.
[38] Roy P, Orecchioni M, Ley K. How the immune system shapes atherosclerosis: roles of innate and adaptive immunity. Nat Rev Immunol 2022;22:251–265.
[39] Woollard KJ, Geissmann F. Monocytes in atherosclerosis: subsets and functions. Nat Rev Cardiol 2010;7:77–86.
[40] Okafor ON, Farrington K, Gorog DA. Allopurinol as a therapeutic option in cardiovascular disease. Pharmacol Ther 2017;172:139–150.
[41] Visseren FLJ, Mach F, Smulders YM, et al. 2021 ESC Guidelines on cardiovascular disease prevention in clinical practice. Eur J Prev Cardiol 2022;29:5–115.
[42] collaboration SwgaECr. SCORE2 risk prediction algorithms: new models to estimate 10-year risk of cardiovascular disease in Europe. Eur Heart J 2021;42:2439–2454.

Keywords: MetALD; ethanol; cardiovascular disease; uric acid; NLRP3 inflammasome.
Received 21 August 2025; received in revised form 9 June 2026; accepted 12 June 2026; Available online 19 June 2026

Journal of Hepatology, Volume *8*

Supplemental information

Alcohol intake reprograms hepatic immune-metabolic circuits to exacerbate murine atherosclerosis and human cardiovascular risk

Constanze Hoebinger, Georg Semmler, Oleksandr Petrenko, Dragana Rajcic, Dorothea Katharina Hoffelner, Tereza Duckova, Taras Baranovskyi, Laura Goederle, My Phung Khuu, Christoph Reinhardt, Petra Pjevac, Joana Séneca, Kamil Mieczkowski, Katharina Burger, Ina Bergheim, Sophie Gensluckner, Andreas Völkerer, Thomas Reiberger, Bernhard Scheiner, Elmar Aigner, Bernhard Wernly, Christian Datz, Christoph J. Binder, and Tim Hendrikx

Alcohol intake reprograms hepatic immune-metabolic circuits to exacerbate murine atherosclerosis and human cardiovascular risk

Constanze Hoebinger, Georg Semmler, Oleksandr Petrenko, Dragana Rajcic, Dorothea Katharina Hoffelner, Tereza Duckova, Taras Baranovskyi, Laura Goederle, My Phung Khuu, Christoph Reinhardt, Petra Pjevac, Joana Séneca, Kamil Mieczkowski, Katharina Burger, Ina Bergheim, Sophie Gensluckner, Andreas Völkerer, Thomas Reiberger, Bernhard Scheiner, Elmar Aigner, Bernhard Wernly, Christian Datz, Christoph J. Binder, Tim Hendrikx

Table of contents

Supplementary materials and methods…………………………………………….……2

Supplementary figures……………………………………………………………...13

Supplementary figure legends………………………………………………………..19

Supplementary tables…………………………………………………………….21

Supplementary references…………………………………………………...….30

Supplementary materials and methods

This study adheres to all relevant ethical regulations. All experimental studies conducted in Vienna were approved by the Animal Ethics Committee of the Medical University of Vienna and the Austrian Federal Ministry of Education, Science, and Research (license no. BMWF 2022-0.574.136). These studies were carried out in accordance with Good Scientific Practice and national and international institutional guidelines. Experimental studies conducted in Mainz were approved by the Landesuntersuchungsamt Rheinland-Pfalz, Koblenz, Germany (license no. G20-1-119) and carried out in accordance with Good Scientific Practice and national and international institutional guidelines.

Human data

Cohort I: 5115 subjects aged 40-69 years undergoing first screening colonoscopy within the prospective SAKKOPI registry between 2007 and 2020 were included (i) if data on alcohol consumption and (ii) laboratory data on lipid levels and uric acid were available (1). Alcohol consumption was evaluated using a structured questionnaire completed by participants before the health check-up. Participants reported the frequency of consuming standard beverage units (0.5 L beer, 0.25 L wine, and 0.02 L liquor/spirits) using predefined categories ("never," "seldom," "once per week," "several times per week," "once per day," "twice per day," or "more than twice per day"). Reported frequencies were converted into estimates of total alcohol intake (grams of ethanol per day), which were subsequently used to classify individuals according to established thresholds for SLD categories.

Cardiovascular risk was assessed by SCORE2 and the Framingham Risk Score (FRS) estimating the 10-year risk of fatal and non-fatal cardiovascular events. Of note, SCORE2 was only calculated in patients aged 40-69, without diabetes and without established cardiovascular disease, while FRS was only calculated in those without established cardiovascular disease.

Cohort II: 2515 first-time referrals to the hepatology outpatient clinic of the University Clinic Salzburg between 2016 and 2024 were included (i) if data on self-reported alcohol consumption and (ii) laboratory data on lipid levels and uric acid were available. Alcohol intake was documented based on patient self-report obtained during routine clinical interviews, and converted into daily ethanol intake for classification according to established SLD thresholds.

For both cohorts, patients with other liver diseases than SLD were excluded. Subjects were stratified into abstainers, low consumption (<20/30g/d), moderate consumption (20-50/30-60g/d) and high consumption (>50/60g/d), respectively. Subgroup analysis excluding statin users (Cohort I: n=781, 15.3%; Cohort II: n=349, 13.9%) were provided.

The study was approved by the local ethics committees (Cohort I: 415-E/1262/2-2010; Cohort II: PMU-EK-2024-0059). All patients from Cohort I provided written informed consent for the analysis of their data.

Patient characteristics were summarized overall and by alcohol groups using descriptive statistics (continuous variables reported as median [IQR] and/or mean [SD], categorical variables as counts and percentages). Group comparisons used Kruskal–Wallis tests for continuous variables and chi-square tests for categorical variables; for normally-distributed variables (uric acid and total cholesterol), one-way ANOVA was used.

Results were visualized for selected variables of interest using violin plots and annotated for significant pairwise differences. Specifically, when the global test was significant ($p \leq 0.05$),

pairwise post-hoc comparisons were performed using Tukey's HSD after ANOVA (family-wise error control) or Dunn's tests with Benjamini–Hochberg adjustment after Kruskal–Wallis. All tests were two-sided with a significance level of 0.05.

Animal models

Ldlr$^{-/-}$ mice were provided by Prof. C. J. Binder, Medical University of Vienna, Vienna, Austria. These mice (B6.129S7-Ldlrtm1Her/J, JAX stock no. 002207) were originally obtained from The Jackson Laboratory and were subsequently bred in-house. All mice were on a C57BL/6J background, and age- and sex-matched littermate controls were used in all experiments. Mice were bred under specific pathogen-free conditions in the Department of Biomedical Research or the Department of Laboratory Animal Science and Genetics of the Medical University of Vienna, Austria.

Age-matched female *Ldlr*$^{-/-}$ mice (9–13 weeks old) were used for all experiments. The mice were bred and maintained in individually ventilated cages (IVCs) under a 12-hour light/dark cycle with ad libitum access to sterilized food and water, under barrier-specific pathogen-free conditions at the Core Facility for Animal Breeding and Husbandry of the Center for Biomedical Research at the Medical University of Vienna. Ambient temperature was maintained at approximately 22 °C (range: 20–24 °C) with a relative humidity of around 55% (range: 45–65%). To induce MASLD and atherosclerosis, mice were fed a high-fat, high-cholesterol diet (HFC) containing 21% milk fat and 0.21% cholesterol (TD88137, ssniff-Spezialdiäten GmbH) with ad libitum access to pellets. To induce MetALD and atherosclerosis, we adapted the protocol described by *Schonfeld et al* (2). Mice received HFC diet with *ad libitum* access to pellets in combination with ethanol in the drinking water. Ethanol was administered as the sole source of drinking water at specified concentrations for treatment durations of 8 or 16 weeks. In the MetALD groups, the concentration of ethanol in the drinking water was progressively increased in a stepwise manner: Mice received 1%, 3%, 10%, 15%, and 20% (v/v) ethanol for 3 days each. Upon reaching 20% (v/v), the regimen continued with an alternating pattern of 20% (v/v) for 4 days followed by 10% (v/v) for 3 days.

For experiments evaluating the role of the intestinal microbiota in liver disease and atherosclerosis development, additional groups were incorporated. In these studies, mice were maintained on the HFC diet for 4 weeks to induce enhanced dyslipidemia and monocytosis, in combination with ethanol in the drinking water. In the MetALD group, mice received 1%, 3%, 10%, 15%, and 20% (v/v) alcohol for 2 days each, and upon reaching 20% (v/v), the regimen continued with alternating 20% (v/v) ethanol for 4 days and 10% (v/v) for 3 days. During the final 3 weeks of the 4-week period, mice received daily oral gavage with an antibiotic cocktail (0.1 mg/g metronidazole, 0.26 mg/g ampicillin, and 0.13 mg/g vancomycin) to deplete the intestinal microbiota. Mice were housed in groups of 2 to 3 per cage, and body weight was assessed weekly. Blood samples and fecal pellets were collected at baseline (prior to antibiotic treatment, week 1), week 4, week 8 and week 16. At the end of the experiment, mice were killed by CO2 overdose under isoflurane anesthesia, and blood was drawn via the vena cava. The number of mice in each experimental group is indicated in the figure legends.

GF and CONV-R *Ldlr*$^{-/-}$ mice shown in Fig. 4 and Fig. S4 were bred at the Translational Animal Research Center of the University Medical Center Mainz. *Ldlr*$^{-/-}$ mice were rederived as GF by aseptic hysterectomy and maintained as a GF mouse colony in sterile flexible film mouse isolator systems. CONV-R *Ldlr*$^{-/-}$ mice were housed under specific-pathogen-free conditions.

All experimental animals were 11-14 weeks old and maintained in EU type II cages under a 12-hour light/dark cycle with ad libitum access to sterilized food and water. Mice received standard autoclaved laboratory diet and water ad libitum, 22 °C ± 2 °C room temperature,a 12-h light-dark cycle, and 55–65% humidity. Induction of MetALD was performed as described above. Mice were housed in groups of 3 to 5 per cage and body weight was assessed twice per week. Blood samples and fecal pellets were collected at week 8. At the end of the experiment, mice were killed by CO2 overdose, and blood was drawn via the vena cava. The number of mice in each experimental group is indicated in the figure legends.

Biochemical analyses in murine models

Blood was collected via tail vein bleeding directly into EDTA collection tubes (Greiner Bio-One, Germany) during the dietary intervention, and postmortem from the vena cava using a 23-gauge needle and transferred into EDTA collection tubes at the experiment's end. All blood collections were performed after a 4-hour fasting period. Plasma was obtained by centrifugation at 2,000 × g for 10 minutes at 8 °C. Plasma and hepatic triglyceride and cholesterol levels were measured according to the manufacturer's instructions using liquid reagents kit (GPO-PAP Triglyceride Liquicolor kit, CHOD-PAP Cholesterol Liquicolor kit, HUMAN Biochemica, and Diagnostica mbH, Wiesbaden, Germany). Protein content was measured using the Pierce BCA Protein Assay Kit (Thermo Fisher Scientific, Waltham, MA, USA). Plasma and hepatic uric acid levels were measured according to the manufacturer's instructions using Uric Acid Assay Kit (ab65344, Abcam, Netherlands). Plasma free fatty acids were quantified using the MAK044-1KT kit (Sigma-Aldrich, Vienna, Austria), and plasma ethanol levels were measured with the Ethanol assay kit (MAK076-1KT, Sigma-Aldrich, Vienna, Austria) following the manufacturer's protocol. Systemic LPS levels were assessed according to the manufacturer's protocol using Mouse Lipopolysaccharides (LPS) ELISA Kit (CSB-E13066m, Hölzel Diagnostika Handels GmbH, Köln, Germany). Hepatic IL-1β protein concentration was determined using the DuoSet™ ELISA Development System Mouse IL-1β/IL-1F2 (DY401), according to the manufacturer's protocol. Plasma immunoglobulins were quantified by sandwich ELISA. IgM levels were measured using anti-mouse IgM for coating (AP500, Sigma-Aldrich, Austria) and alkaline-phosphatase–conjugated anti-mouse IgM for detection (A9688, Sigma-Aldrich, Austria), as previously described (3). For IgG1, plates were coated with anti-mouse IgG1 (clone RMG1-1; 406602, BioLegend, San Diego, USA) and detected with biotinylated anti-mouse IgG1 (clone A85-1; 553441, Becton Dickinson, Austria). Total IgA concentrations were measured using a chemiluminescent sandwich ELISA with anti-mouse IgA for capture (clone C10-3; 556969, Becton Dickinson, Austria) and biotinylated anti-mouse IgA for detection (clone C10-1; 556978, Becton Dickinson, Austria).

RNA Isolation

For whole-liver RNA isolation, 50 mg tissue samples were collected from the left lateral liver lobe and snap-frozen. For aortic RNA isolation, the entire aorta was harvested and snap-frozen. For small intestine RNA isolation, a small portion of the whole intestinal tissue was snap-frozen in liquid nitrogen and used for RNA extraction. The frozen tissues were homogenized in QIAzol lysis reagent (Cat. No. 79306, Qiagen, Hilden, Germany) using 1.0 mm TriplePure M-Bio Grade High Impact Zirconium beads (Lot: 44544432, Benchmark) in a TissueLyser II (Qiagen, Hilden, Germany). RNA extraction was performed according to the manufacturer's

instructions, and RNA quantity and quality were subsequently assessed using a NanoDrop spectrophotometer (Peqlab).

cDNA Synthesis and Quantitative Real-Time PCR (qPCR)

Up to 1 µg of total RNA was reverse transcribed into cDNA using the High-Capacity cDNA Reverse Transcription Kit (Applied Biosystems, Thermo Fisher, Waltham, MA, USA) following the manufacturer's protocol. qPCR was carried out on a CFX96 Real-Time PCR System (Bio-Rad Laboratories, Hercules, CA, USA) using the KAPA SYBR FAST kit (Thermo Fisher, Waltham, MA, USA) with the primers listed below. Relative gene expression was calculated by the $2^{(-\Delta\Delta Ct)}$ method, with normalization to the housekeeping gene 18S. Primer sequences are provided in Table 4.

Primer details

Target Gene	Forward Sequence (5'-3')	Reverse Sequence (5'-3')
Abca1	GAGCAAAGCCAAGCATCTTC	AGCAGGGACCACATAATTGC
Abcg4	CTGAGCTCTTCCAACACTTCG	GCCACAGAACACCAACTCTC
Abcg8	CTCACCTACCAGGTGGACAT	GCATCTGTCCACTCCTCACT
Adh1	GGGTTCTCAACTGGCTATGG	ACAGACAGACCGACACCTCC
Aim2	AGGCTGCTACAGAAGTCTGTCC	TCAGCACCGTGACAACAAGTGG
Casp1	GGCACATTTCCAGGACTGACTG	GCAAGACGTGTACGAGTGGTTG
Ccl2	AGGTCCCTGTCATGCTTCTG	TCTGGACCCATTCCTTCTTG
Cd36	GCCAAGCTATTGCGACATGA	AAAAGAATCTCAATGTCCGAGACTTT
Cldn1	ATGCCAATTACCATCAAGGC	GAGGGACTGTGGATGTCCTG
Cldn2	TTCTTGGATCCGAGCCTCTA	TCCAGAGCTCTTCGAAAGGA
Cxcl1	GCTGGGATTCACCTCAAGAA	TCTCCGTTACTTGGGGACAC
Cxcl2	AGTGAACTGCGCTGTCAATG	TTCAGGGTCAAGGCAAACTT
Cyp2e1	CTTAGGGAAAACCTCCGCAC	GGGACATTCCTGTGTTCCAG
Cyp7a1	CACCATTCCTGCAACCTTCTGG	ATGGCATTCCCTCCAGAGCTGA
Fatp4	GACTTCTCCAGCCGTTTCCACA	CAAAGGACAGGATGCGGCTATTG
Fut2	GGTGGATGATGGTGGAAGTC	TTCCCTGTTACCACAGCCAG
Il1b	TGTGCAAGTGTCTGAAGCAGC	TGGAAGCAGCCCTTCATCTT
Il18	GACAGCCTGTGTTCGAGGATATG	TGTTCTTACAGGAGAGGGTAGAC
Lpl	GGGAGTTTGGCTCCAGAGTTT	TGTGTCTTCAGGGGTCCTTAG
Muc2	AAGTTTGCCCCTGGCTATGAC	CCTTGCAGTCAAACTCAAAGT
Nlrp3	TCACAACTCGCCCAAGGAGGAA	AAGAGACCACGGCAGAAGCTAG
Npc1l1	ATCGCACTACCATCCAGGACCT	CCCAGAGTAGCCTTGGAATCCA
Ocln	ATTTATGATGAACAGCCCCC	CATAGTCAGATGGGGGTGGA
Reg3b	GGCTTCATTCTTGTCCTCCA	TCCACCTCCATTGGGTTCT
Reg3g	AAGCTTCCTTCCTGTCCTCC	TCCACCTCTGTTGGGTTCAT
S100a8	AAATCACCATGCCCTCTACAAG	CCCACTTTTATCACCATCGCAA
Srebf1	CGACTACATCCGCTTCTTGCAG	CCTCCATAGACACATCTGTGCC
Srebf2	AGAAAGAGCGGTGGAGTCCTTG	GAACTGCTGGAGAATGGTGAGG
Trem2	CTACCAGTGTCAGAGTCTCCGA	CCTCGAAACTCGATGACTCCTC

Zo-1	AGCTGTTTCCTCCATTGCTG	GAGATGTTTATGCGGACGG

Flow Cytometry

For flow cytometric analysis, livers were harvested, flushed *in situ* with PBS to reduce blood-derived contamination, mechanically dissociated, and enzymatically digested for 30 minutes at 37 °C in PBS supplemented with 450 U/ml collagenase I (LifeTech Austria, Vienna, Austria), 125 U/ml collagenase XI (Sigma-Aldrich, Vienna, Austria), 60 U/ml DNase I (LifeTech Austria, Vienna, Austria), and 60 U/ml hyaluronidase (Sigma-Aldrich, Vienna, Austria). The digested tissue was passed through a 40µm cell strainer to obtain a single-cell suspension, and residual erythrocytes were removed using red blood cell lysis buffer (Morphisto, Offenbach am Main, Germany). Blood was collected as described above and also processed with red blood cell lysis (Morphisto, Offenbach am Main, Germany) before the staining procedure. Cells were first incubated with an anti-mouse CD16/32 blocking antibody (clone 93, 0.5 µg per 0.5×10^6 cells; eBioscience) for 20 minutes at 4 °C. This was followed by staining for 30 minutes with a mixture of the following antibodies: BV605-labeled anti-Ly6C (clone HK1.4, 1:1000, BioLegend), PerCP-Cy5.5-labeled anti-CD45 (clone 30-F11, 1:800, eBioscience), FITC-labeled anti-CD11b (clone M1/70, 1:400, BioLegend), PE-labeled anti-CD3e (clone 145-2C11, 1:400, eBioscience), PE-Cy7-labeled anti-Ly6G (clone 1A8, 1:400, BD Biosciences), APC-eFluor780-labeled anti-CD11c (clone N418, 1:800, eBioscience), Alexa Fluor700-labeled anti-B220/CD45R (clone RA3-6B2, 1:800, BioLegend), APC-labeled anti-F4/80 (clone BM8, 1:800, BioLegend), PerCP-Cy5.5-labeled anti-CD115 (CSF-1R) (clone AFS98, 1:400, BioLegend), FITC-labeled anti-CD45 (clone 30-F11, 1:400, BioLegend), and APC-Vio770-labeled anti-B220/CD45R (clone REA755, 1:800, Miltenyi Biotec). All staining procedures were performed in 100 µl of FACS buffer (PBS supplemented with 2% FBS) for 30 minutes at 4 °C in the dark, followed by two washing steps. Stained cell populations were recorded using a BD FACS Fortessa multiparameter flow cytometer. During analysis using FlowJo version 10 (Tree Star Inc., Ashland, OR, USA), dead cells and doublets were excluded based on forward- and side-scatter. Gating strategies to identify hepatic immune cell populations by flow cytometry: Kupffer cells ($CD45^+$ $Ly6G^-CD11b^+F4/80^+$), monocytes ($CD45^+CD11b^+F4/80^-Ly6G^-$), $Ly6C^{high}$ monocytes ($CD45^+CD11b^+Ly6G^-F4/80^{-/low}Ly6C^{high}$), neutrophils ($CD45^+Ly6G^+CD11b^+F4/80^-$), T cells ($CD45^+B220^-CD3^+$), and B cells ($CD45^+B220^+CD3^-$). Gating strategies to identify systemic immune cell populations by flow cytometry: monocytes ($CD115^+CD11b^+Ly6G^-$), $Ly6C^{high}$ monocytes ($CD45^+CD115^+CD11b^+Ly6G^-Ly6C^{high}$), neutrophils ($CD11b^+Ly6G^-$), T cells ($B220^-CD3^+$, and B cells ($B220^+CD3^-$).

Western blot

Liver

Western blotting was done as previously described (4). Briefly, liver protein samples were prepared in RIPA buffer (50 nM Tris-HCl, pH 8, 0.5% SDS, 150 mM NaCl, 1% Triton X-100, 0.5% sodium deoxycholate) supplemented with protease inhibitor cocktail tablets (Roche) and phosphatase inhibitor cocktail 2 (Sigma-Aldrich). Protein concentration was measured using the Pierce BCA Protein Assay Kit (Thermo Fisher Scientific, Waltham, MA, USA). 50 µg of protein per lane were loaded, resolved in SDS-PAGE, and transferred onto a nitrocellulose

membrane. The membranes were blocked in 5% skimmed milk in PBS-T and immunoblotted overnight with the following primary antibodies at 4 °C: SREBP1 (Abcam, clone 2A4, dilution 5 µg/mL), SREBP2 (Abcam, dilution 1:500), TUBULIN (Abcam, dilution 1 µg/mL), NLRP3 (eubio, dilution 1 µg/mL), and ß-ACTIN (Abcam, 1 µg/mL). Next, after washing steps in PBS-T, secondary antibodies ECL™ donkey anti-rabbit IgG-HRP (Amersham) or ECL™ sheep anti-mouse IgG-HRP (Amersham) were used. Chemiluminescence detection was performed with Clarity™ Western ECL Substrate (Bio-Rad) and acquired with a ChemiDoc Imager (Bio-Rad). Densitometry quantification of bands representing detected proteins was done with ImageJ software. Densitometry data are presented as mean cleaved-to-precursor protein ratios adjusted to the loading control Tubulin. Densitometry quantification was performed on all available samples.

Intestine

RIPA protein lysates of small intestinal tissue (20 µg/lane for OCCLUDIN; 10 µg/lane for ß-ACTIN) were separated on 10% SDS-polyacrylamide gels and transferred to polyvinylidene difluoride membranes (Bio-Rad Laboratories) as detailed before (5). Membranes were further incubated with specific primary antibodies (Occludin, Invitrogen; ß-Actin, Santa Cruz) and the respective secondary antibody (anti-mouse IgG, HRP-linked; Cell Signaling Technology, Massachusetts, USA). To detect the protein bands, the Super Signal Western Dura kit (Thermo Fisher Scientific) was used, and densitometric analysis were performed using ChemiDoc XRS System (Bio-Rad Laboratories) as detailed previously (6).

Histology and immunohistochemistry

Liver

The left lobes of the livers were harvested and divided into four equal pieces, each snap-frozen in liquid nitrogen for subsequent analyses. Frozen liver sections (7 µm) were prepared and stained for infiltrating neutrophils as previously described by us (7). Neutrophils were detected using a mouse anti-Ly6G antibody (1:100; BD PharMingen), and cell nuclei were counterstained with hematoxylin (Klinipath). The number of positively stained cells was counted in 5 pictures per liver per mouse to determine the level of liver inflammation. To assess lipid content, additional frozen liver sections were stained with Oil Red O (Sigma-Aldrich, Vienna, Austria) and quantified as previously described (8). Pictures were taken with a Zeiss AxioImager A1 microscope equipped with an AxioCam MRC5 camera and ZEN 2.3 Pro software (Carl Zeiss). Formalin-fixed liver samples (Tissues were collected and stored in 3.7% formaldehyde for 16-24h, and then transferred to PBS and kept at 4°C) were embedded in paraffin, and 4 µm sections were stained with Mayer's Hematoxylin solution (254766, AppliChem, Darmstadt, Germany) and Eosin Y solution (HT110321, Sigma-Aldrich, Vienna, Austria) for assessing general tissue morphology. Collagen deposition was evaluated by Sirius Red staining of liver sections as previously described (9). F4/80 immunohistochemical staining was performed on paraffin-embedded liver sections. Slides were deparaffinized and rehydrated, followed by heat-induced antigen retrieval in citrate buffer (pH 6.0; Sigma-Aldrich) at 98-99 °C. Sections were blocked with 10% normal donkey serum (Sigma-Aldrich) in PBS containing 0.05% Tween-20, followed by avidin/biotin blocking (Abcam) according to the manufacturer's instructions. Sections were then incubated overnight at 4 °C with a rabbit anti-

mouse F4/80 primary antibody (1:500; Cell Signaling Technology, clone D2S9R) diluted in PBS containing 4% fetal bovine serum. Endogenous peroxidase activity was quenched using 3% H_2O_2 in PBS/Tween. Slides were subsequently incubated with a biotinylated goat anti-rabbit IgG secondary antibody, followed by streptavidin-horseradish peroxidase (1:1000; Sigma-Aldrich). Signal detection was performed using a DAB substrate kit (DAKO). Sections were counterstained with hematoxylin, mounted using Aquamount (Merck), and imaged using a Zeiss AxioImager A1 microscope equipped with an AxioCam MRC5 camera and ZEN 2.3 Pro software (Carl Zeiss). Images were analyzed for F4/80$^+$ macrophages using ImageJ (v1.53), and the percentage of positive staining per liver section was quantified. At least five randomly selected fields per liver section were analyzed for each mouse.

Aorta, heart, and innominate artery preparation:
Immediately post-mortem, mice underwent perfusion via the left ventricle first with 20 mL of PBS, (followed by perfusion with 3.7% formaldehyde (Merck Millipore) for mice undergoing 8 weeks dietary regimen). Perivascular tissue was removed in situ, and the hearts, brachiocephalic (innominate) arteries, and aortas (extending to the iliac bifurcation) were isolated and stored in 3.7% formaldehyde for 16–24 hours. Tissues were then transferred to PBS until further processing. Ventricular portions were excised along with the atria to expose the aortic root for subsequent cross-sectional analyses.

Aortic root cross-section and lesion quantification
8-week dietary intervention study: Hearts were prepared and analyzed as previously described by us (10). 16-week dietary intervention study: Hearts, including the aortic root, were embedded in OCT compound, and aortic root sections were prepared using a cryostat (Leica CM1950) at a thickness of 10 µm, beginning at the anatomical level where all three aortic valve leaflets became visible. Sections were sequentially collected throughout the entire aortic root region. For cross-sectional analysis, sections were stained with Mayer’s Hematoxylin solution (254766, AppliChem, Darmstadt, Germany) and Eosin Y solution (HT110321, Sigma-Aldrich, Vienna, Austria). Images were photographed on a Zeiss AxioImager A1 using AxioCam MRC5 and ZEN 2.3 Pro software (Carl Zeiss). Lesion size in all three leaflets was assessed by computer-assisted image analysis using Photoshop Elements and ImageJ software. For cross-sectional lesion analysis, data are presented as the average total lesion size across eight sections collected at 80 µm intervals, spanning 560 µm of the aortic root, with total lesion size defined as the sum of lesions at the three leaflets at each location.

8-week dietary intervention study in GF and CONV-R mice: Hearts, including the aortic root, were fixated in 4% PFA for 24 h. Paraffin embedding was performed by the Histology Core Facility (University Medical Center Mainz, Germany). Serial cross-sections of the aortic root were collected at a thickness of 5 µm using a rotatory microtome (Leica Biosystems) and stained with Trichrome Stain Kit (ab150686, Abcam, Cambridge, UK) for atherosclerotic lesion size. Images were acquired on an Olympus BX51 microscope (Olympus). Lesion size in all three leaflets was assessed by computer-assisted image analysis using CellSens Dimension (v.4.1) (Carl Zeiss). For cross-sectional analysis, lesion size was expressed as the average total lesion area across eight sections, collected at 50 µm intervals over a span of 400 µm over the

aortic origin, with total lesion size defined as the sum of lesions at the three leaflets at each location.

Aorta Sudan IV staining and lesion quantification

For *en face* preparations, aortas were pinned onto standard wax dissection pans submerged in PBS, and remaining adventitial tissue was removed. The aortas were washed in Sørensen's phosphate buffer (pH 7.38) and subsequently incubated for 15 min in Sudan IV Staining Solution (0.5% Sudan IV in 1:1 acetone and 70% ethanol, filtered; Sigma-Aldrich) with periodic shaking. Aortas were subsequently incubated in 80% ethanol for 5 min, followed by washing in water. Images were acquired on a Zeiss Stemi Dissecting Microscope with Axiocam 208 Color and ZEN Blue software (Carl Zeiss). Lesion size was assessed in a double-blinded fashion by computer-assisted analysis using Photoshop Elements (Adobe) and ImageJ. Data are expressed as the percent of Sudan IV^+ area per total aortic area.

Immunohistochemistry of the heart

Lesional macrophage content was assessed by immunostaining of paraffin-embedded sections obtained after 8-week dietary intervention using antibodies against MAC2 (BioLegend, #125401, clone M3/38; dilution 1:1,500) and analyzed as previously described (10). Cryosections from mice subjected to 16-week dietary intervention were stained according to the same protocol using adjusted antibody dilutions (1:300). For cryosections, dehydration and antigen retrieval were omitted. Instead, sections were air-dried for 45 min, incubated in ice-cold acetone for 10 min, followed by an additional 15 min air-drying step prior to initiation of the staining procedure.

Sirius Red staining of the heart

For quantification of collagen deposition, paraffin-embedded sections and cryosections of aortic root lesions were stained with Sirius Red F3B (CI 35782; Sigma-Aldrich). Paraffin- and cryosections were prepared as described above. After washing in PBS and rinsing in distilled water, sections were treated with 0.2% phosphomolybdic acid and stained with 0.1% Sirius Red in picric acid. Sections were subsequently washed in 0.01 N HCl, dehydrated, cleared, and mounted with Entellan. Collagen content within aortic root lesions was quantified using Photoshop Elements and ImageJ software.

Transcriptomic data acquisition and analysis

Data acquisition

Sequencing libraries from total RNA of the samples were prepared at the Core Facility Genomics, Medical University of Vienna, using the NEBNext Poly(A) mRNA Magnetic Isolation Module and the NEBNext UltraExpress RNA Library Prep Kit for Illumina with Unique Dual Indices (UDIs) according to manufacturer's protocols (New England Biolabs). Libraries were QC-checked on a Bioanalyzer 2100 (Agilent) using a High Sensitivity DNA Kit for correct insert size and quantified using Qubit dsDNA HS Assay (Invitrogen). Libraries were sequenced on a P3 flowcell of a NextSeq2000 instrument in 1x50bp single-end sequencing mode. On average, 34 million reads per sample were generated. Reads in fastq format were

generated using the Illumina bcl2fastq command line tool (v2.19.1.403) incl. trimming of the sequencing adapters.

Data analysis

This analysis has been completed in R (v.4.3.3). Protein-coding genes were identified with biomaRt and utilized in the downstream analysis (11). The exploratory principal component analysis has been performed using PCAtools with the removal of 10% of genes with the lowest variance. Outlier identification has been performed based on the intersections of the first two principal components, after which the outliers (n = 2) were removed.

DESeq2 (v.1.40.2) with the design formula ~group was used for differential expression analysis using pairwise Wald testing (12). The shrinkage of log_2(Fold Change) inflation has been performed using ashr (v.2.2-63) (13). Multiple testing correction has been performed with independent hypothesis weighting (IHW, v.1.28.0) (14). The thresholds for differentially expressed genes (DEGs) were set as follows: shrunk log2(Fold Change) > |0.27| (equalizing to >20% expression difference between the groups), $P_{adjusted} < 0.05$. The DEGs visualization has been made with EnhancedVolcano (v.1.20.0)

Functional analysis of DEGs has been performed using clusterProfiler (v.4.8.1) (15) in gene set enrichment analysis mode against Reactome, Biocarta, Immunosig and Wikipathways databases. For this analysis, genes were ranked using the $sign(log_2FoldChange) \times -log_{10}(Pvalue)$ formula. For ranking genes of the principal components and network members, gProfiler2 (v.0.2.3) (16) with the over-representation testing were used against Gene Ontology (MF), KEGG, Reactome, and WikiPathways databases. The Z-normalized expression differences of all genes of the selected significant pathways from the over-representation testing were then presented as heatmaps.

Inflammasome activation analysis

A curated list of 192 inflammasome activation-related genes was derived from the KEGG mmu04621 pathway. For variance analysis genes without variance across samples or those without detected transcripts in all samples were removed. For variance analysis and visualization, DESeq2-normalized (v.1.40.2) (12), and scaled counts of the remaining genes were used, and displayed as per-sample variance using pheatmap (v.1.10.12).

For the whole pathway-level analysis, we employed gene set variance analysis (GSVA, v.1.50.5) (17). In brief, we used full counts matrix normalized with DESeq2's Variance stabilizing transformation as input for GSVA with Gaussian kernel density estimation, receiving pathway activity score per-sample. The normality of the score distribution was assessed using the Shapiro-Wilk test independently for each experimental group. Homogeneity of variance between group was tested using Levene's test (median-centered), confirming the assumption of equal variances ($p = 0.57$). The normality assumptions were satisfied as well ($p > 0.05$ for both groups), hence a Welch's two-sample t-test was conducted to determine difference in the pathway activation.

Data Availability Statement

The data for this study have been deposited in the European Nucleotide Archive (ENA) at EMBL-EBI under accession number PRJEB106963.

Metabolomics

Plasma extraction

Twenty microliters of plasma sample were spiked with a mixture of heavy isotope labeled internal standards. Samples were extracted using methanol and ultrasonic assisted extraction (on ice). Samples were centrifugated and the supernatants were evaporated using a nitrogen flow. Dry samples were reconstituted with ultrapure water before the HPLC-MS analysis.

Hydrophilic metabolites acquisition

A 1290 Infinity II UHPLC system (Agilent Technologies) coupled with a 6470 triple quadrupole mass spectrometer (Agilent Technologies) was used for the LC-MS/MS analysis. Chromatographic separation was performed on a ZORBAX RRHD Extend-C18, 2.1 x 150 mm, 1.8 µm analytical column (Agilent Technologies) in ion pairing mode, maintained at 40°C. A 4 µL sample was injected per run. The mobile phase A consisted of 3% methanol (v/v), 10 mM tributylamine, and 15 mM acetic acid in water, while mobile phase B contained 10 mM tributylamine and 15 mM acetic acid in methanol. A gradient elution at a flow rate of 250 µL/min was conducted for 24 minutes. Following this, back-flushing of the column was done using a 6 port/2-position divert valve with acetonitrile for 8 minutes, followed by 8 minutes of column equilibration with 100% mobile phase A. The triple quadrupole mass spectrometer was operated in negative electrospray ionization mode, with a spray voltage of 2 kV, gas temperature of 150°C, gas flow of 1.3 L/min, nebulizer pressure of 45 psi, sheath gas temperature of 325°C, and sheath gas flow of 12 L/min. Metabolites were detected using dynamic MRM mode. Ten-point calibration curves with internal standardization were used for absolute quantification. Data processing was performed using PeakBot software (version 0.9.54) (18).

Metabolomic data analysis

Metabolites with missing values at threshold ≥ 20% were excluded from the analysis (n = 62, 30.8%). The remaining 139 metabolites were processed with POMA (19). The missing values were imputed with kNN and relative abundances were normalized using the log-Pareto method (20). The exploratory principal component analysis was performed using PCAtools, which removed 10% of the genes with the lowest variance. Differential abundance and over-representation analysis were performed with MetaboAnalystR (21). Briefly, compound names were mapped against metabolic databases, and their differential abundance was quantified using the MetaboAnalyst FoldChange module. The metabolites reaching >|0.27| log2FoldChange threshold (i.e., >20% abundance difference) were considered as significant. For the enrichment analysis, MetaboAnalystR implementation of the hypergeometric test was applied, and normalized enrichment scores were used to identify up-/downregulated pathways.

16S rRNA amplicon sequencing of the fecal microbiome

DNA was extracted from fecal/cecum samples using the QIAamp Fast DNA Stool Mini Kit (Qiagen), and 16S rRNA gene amplification, barcoding and sequencing of the hypervariable

V4 region of bacterial and archaeal 16S rRNA genes was performed at the Joint Microbiome Facility (JMF) of the Medical University of Vienna and the University of Vienna under Project ID JMF-2307-04 as previously described by us (22). Amplicon libraries were sequenced on an Illumina Miseq (v3 chemistry), and amplicon pools were extracted from raw sequencing data using the FASTQ workflow in BaseSpace (Illumina) with default parameters. Data was filtered for PhiX contamination with BBDuk (BBTools, Bushnell B), demultiplexing was done using the python package demultiplex (Laros JFJ) allowing one mismatch to barcodes, and two mismatches for linker sequences and primers. Barcodes, linkers, and primers were trimmed off using BBDuk, and forward and reverse FASTQ reads were trimmed at 220 and 150 nt, respectively, with allowed expected errors of 2, followed by ASV inference using the recommended DADA2 workflow in R (23, 24). Taxonomic classification against the SILVA (25) SSU Ref NR 99 rRNA database v138.1 was performed using the classifier implemented in DADA2 using a confidence threshold of 0.5. All downstream analyses were performed in R version 4.0.2.

Data Availability Statement
The 16S rRNA gene amplicon sequencing datasets supporting the conclusions of this article are available in the NCBI repository under BioProject accession number PRJNA1261036.

Statistical analysis
Statistical analyses were performed using GraphPad Prism v10. Data comparing the groups were analyzed by two-tailed unpaired Student's t-tests following evaluation of Gaussian distribution. Results are expressed as mean ± standard error of mean (SEM) unless stated otherwise.

Additional details are provided in the Supplementary CTAT Table.

Supplementary figures

Supplementary Figure 1

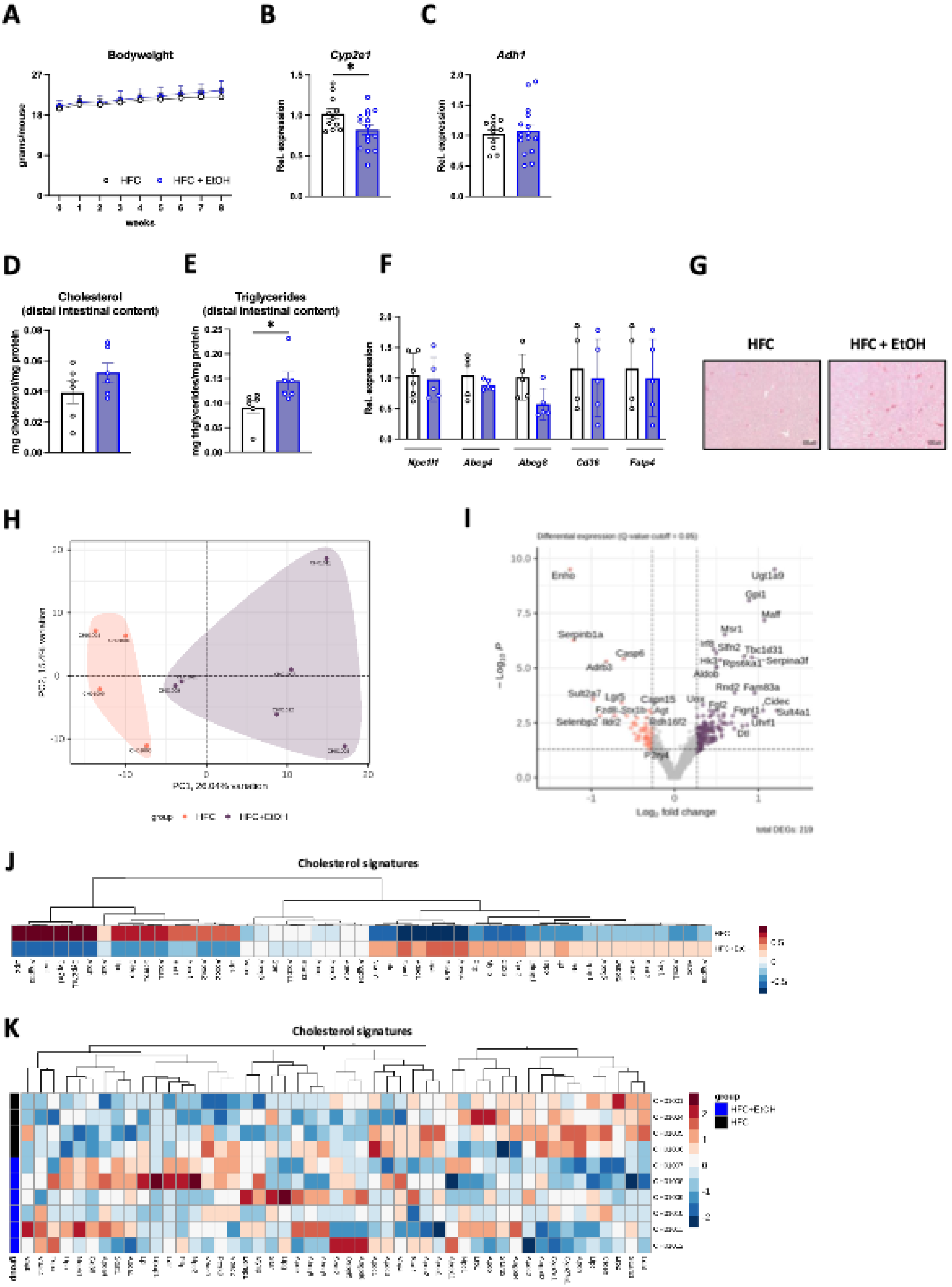

Supplementary Figure 2

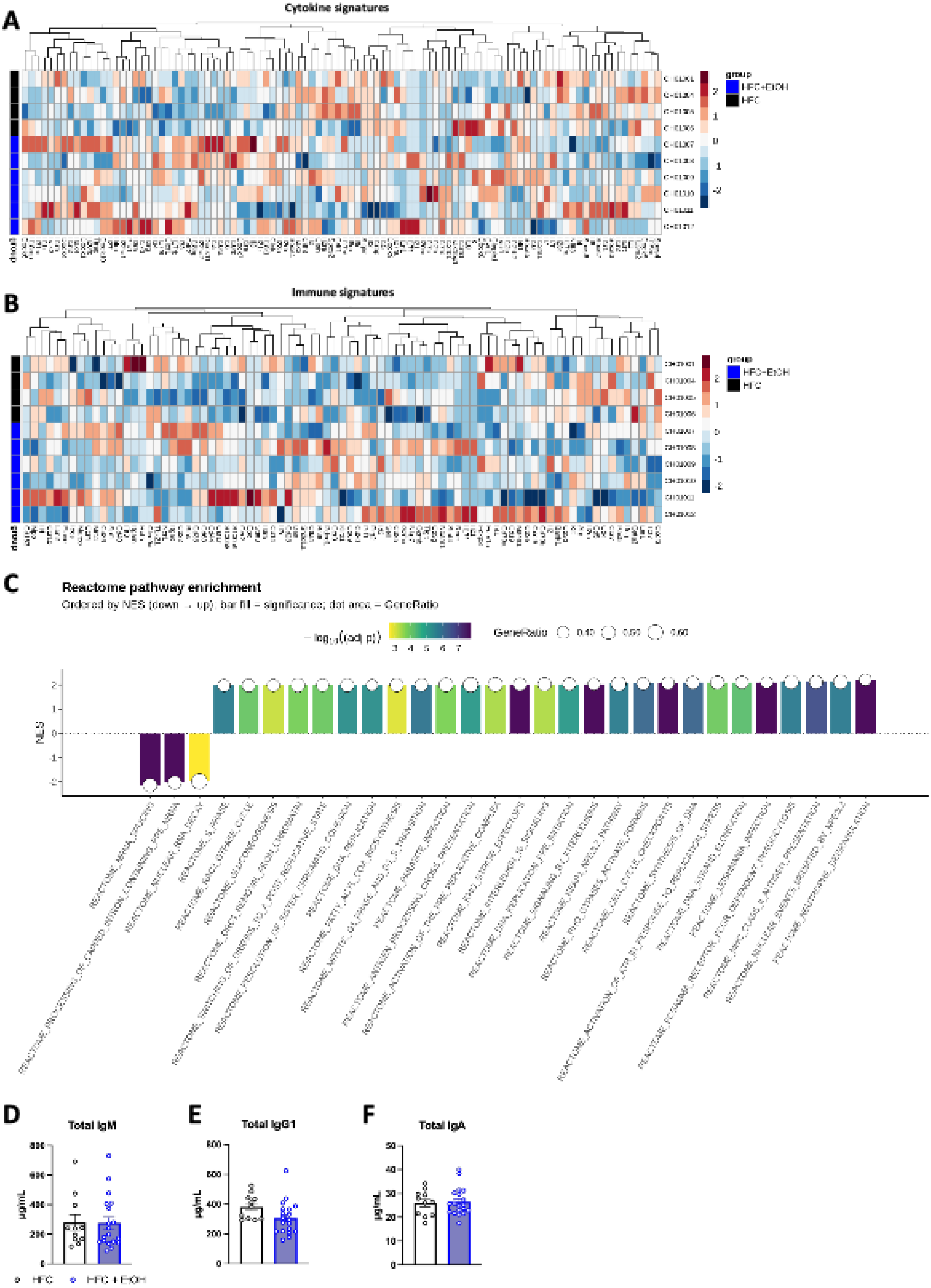

Supplementary Figure 3

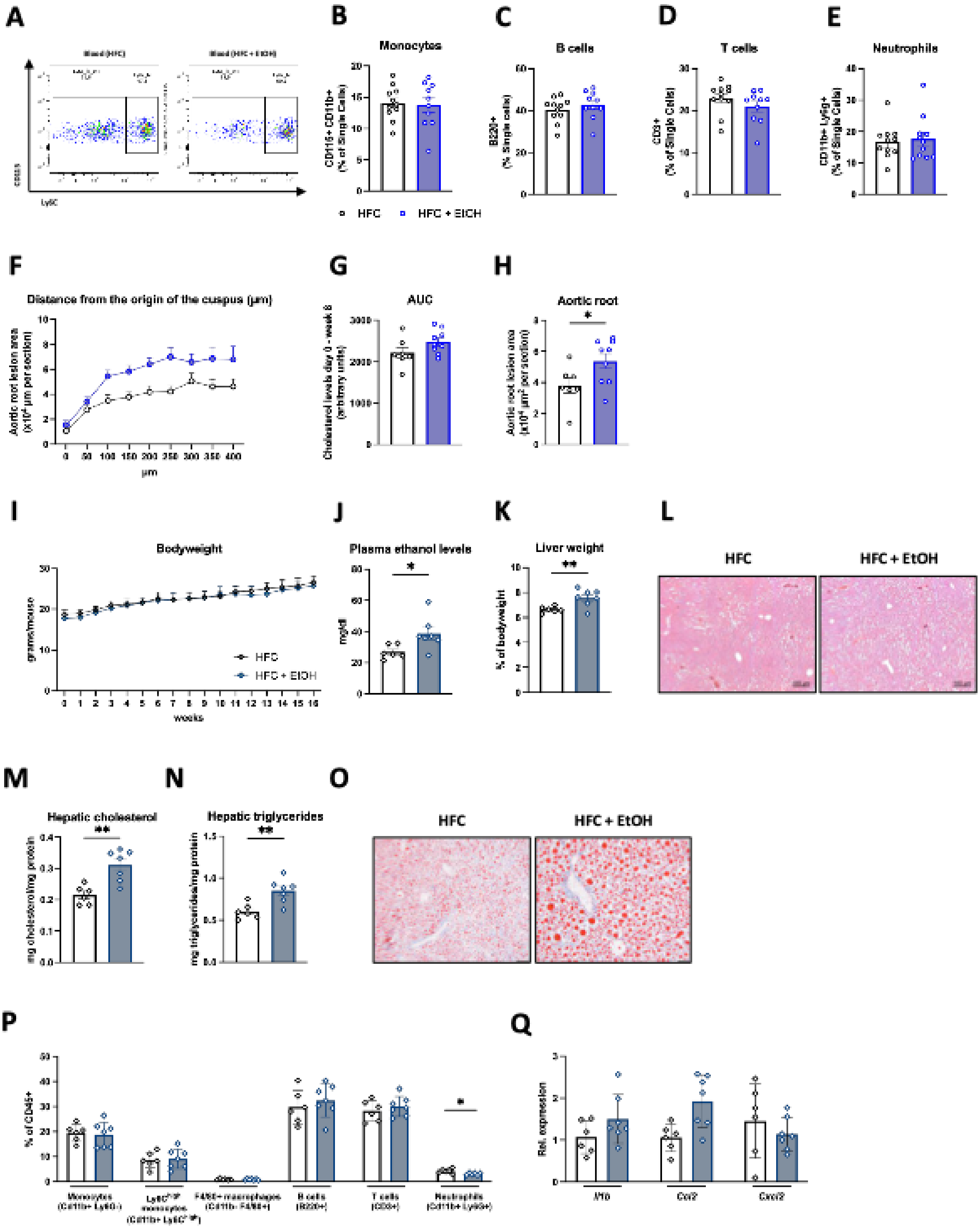

Supplementary Figure 4

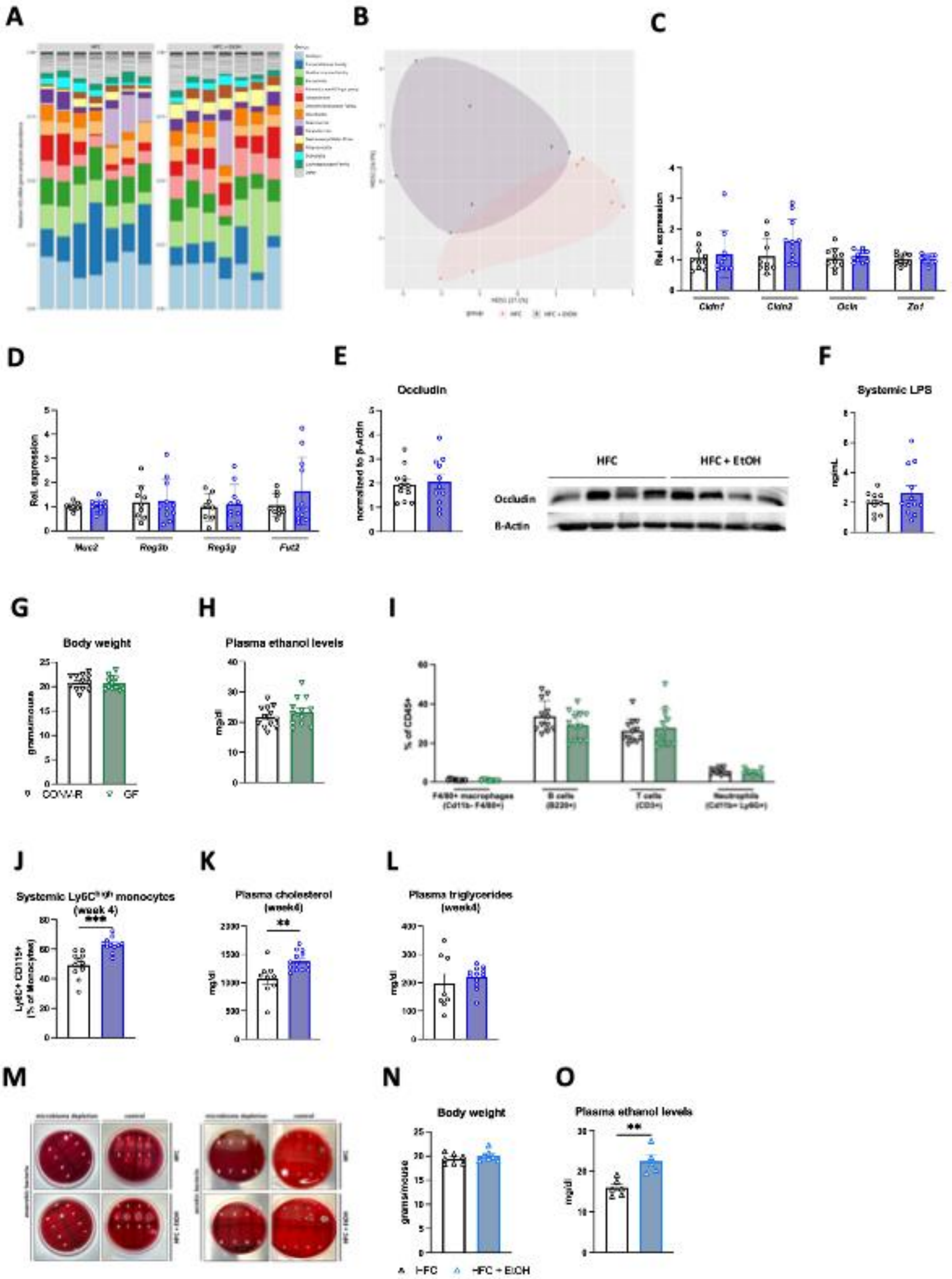

Supplementary Figure 5

A

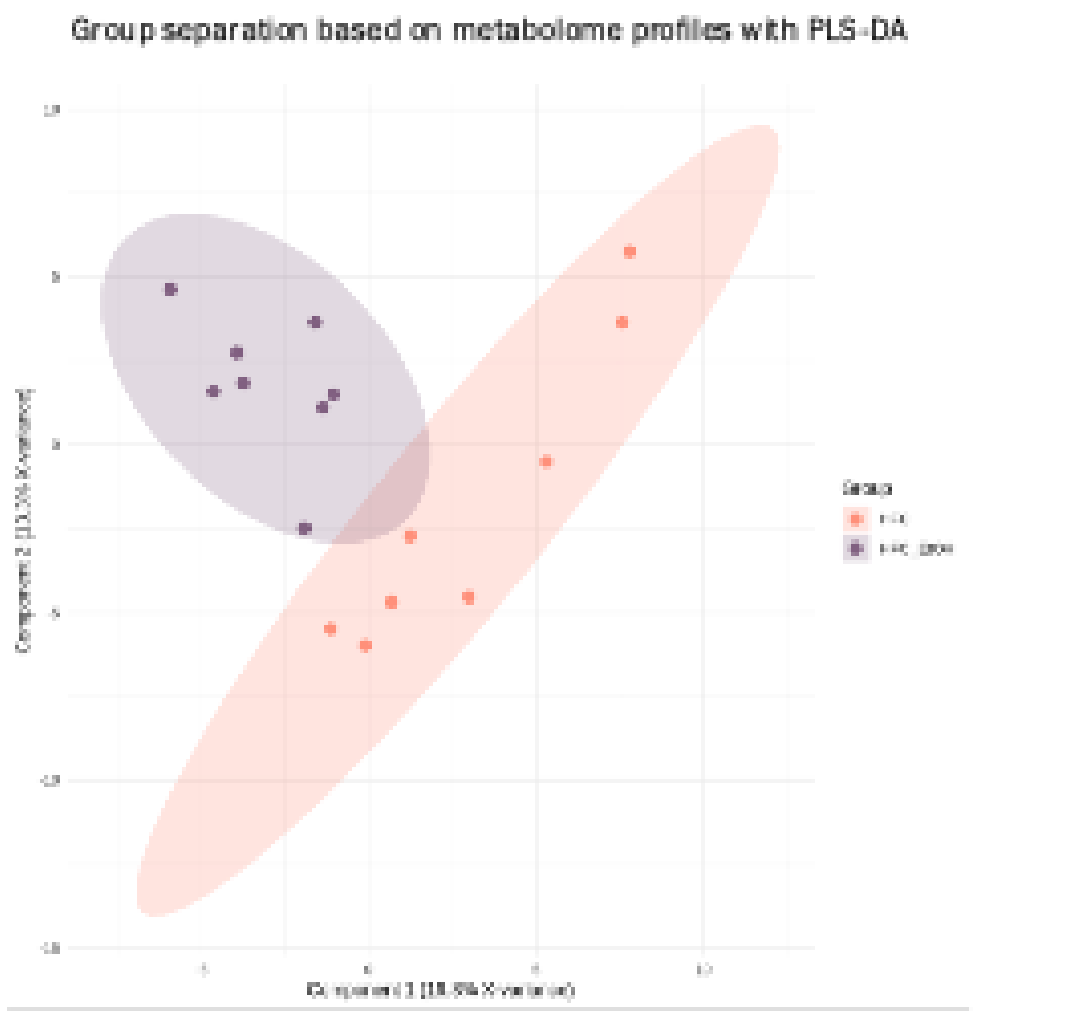

B

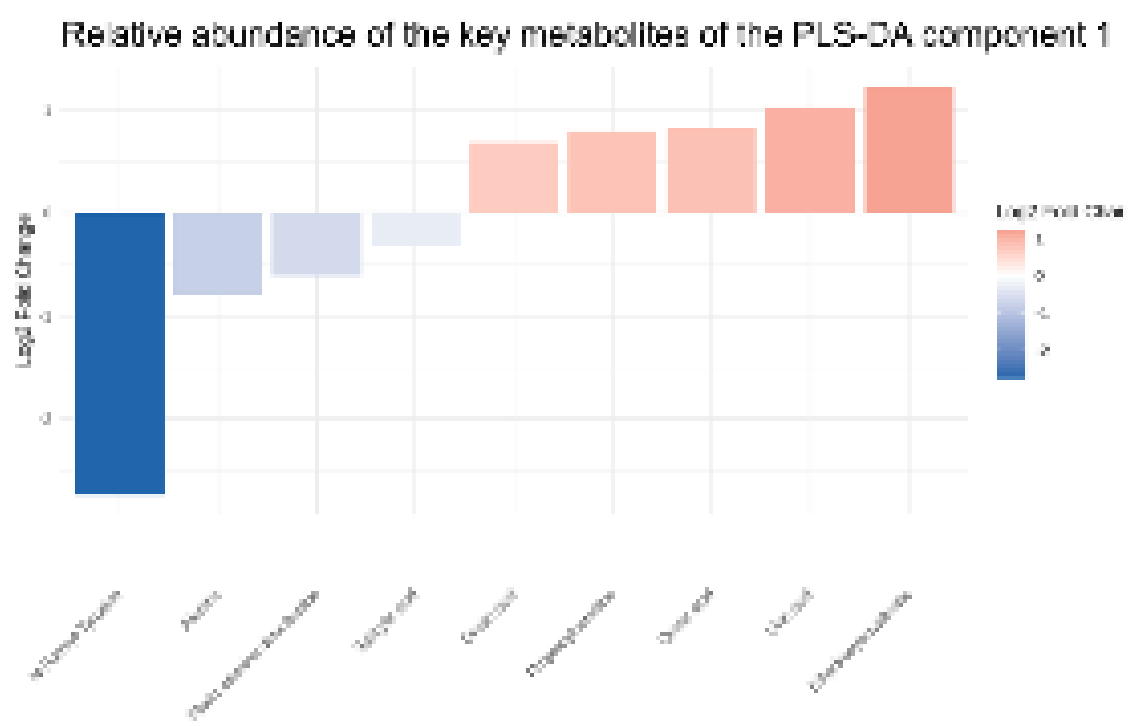

C

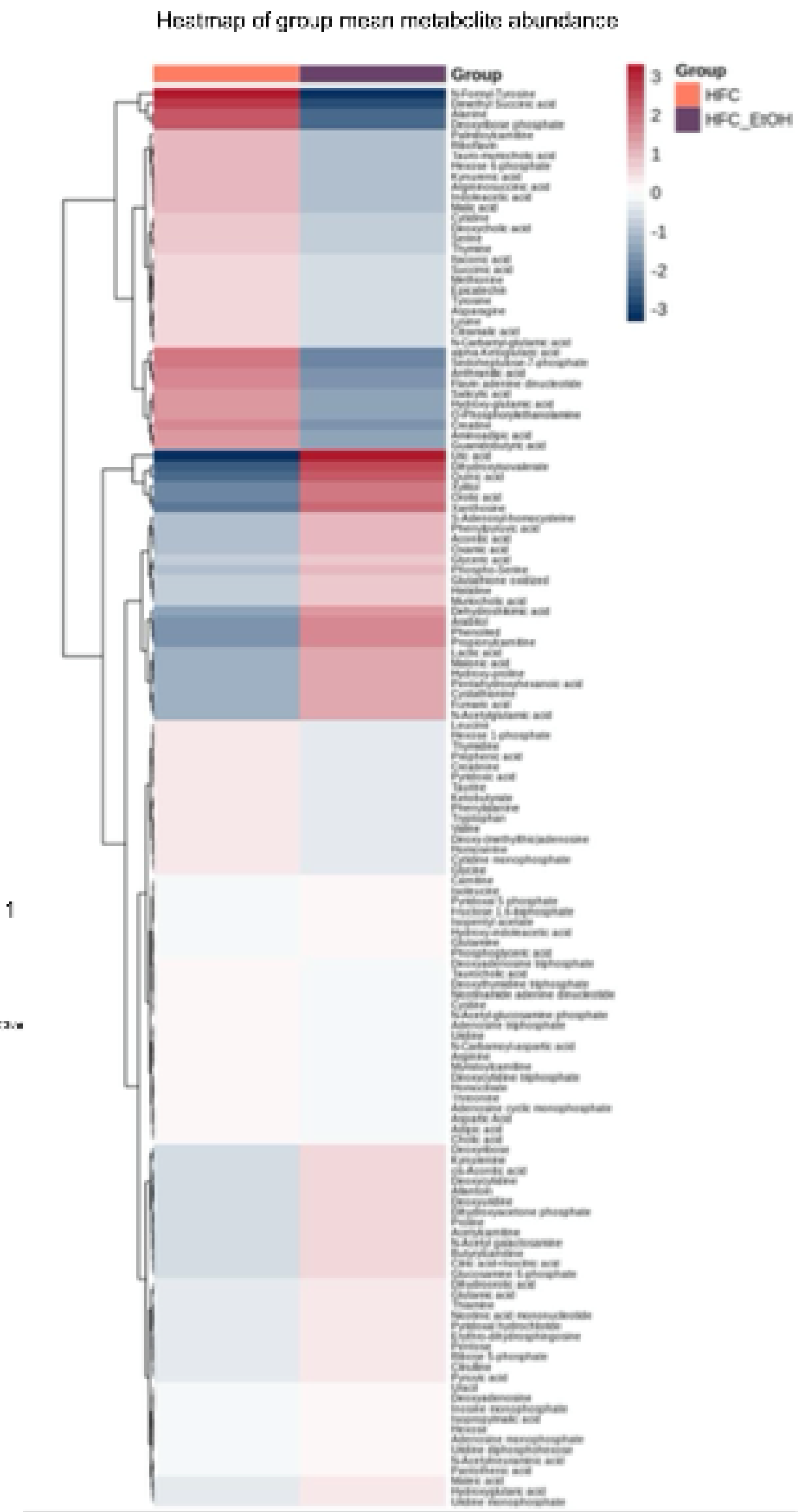

D

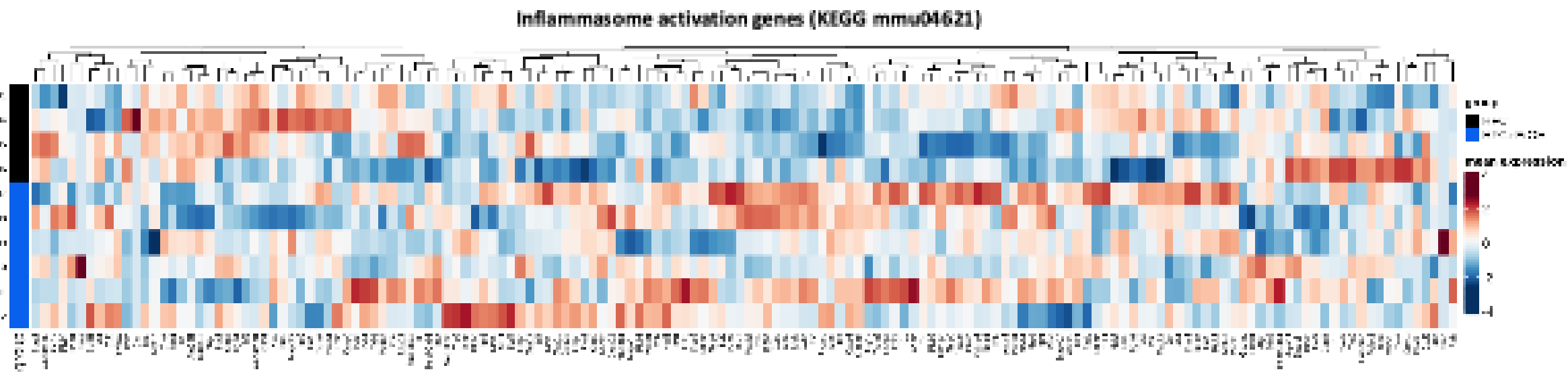

Supplementary Figure 6

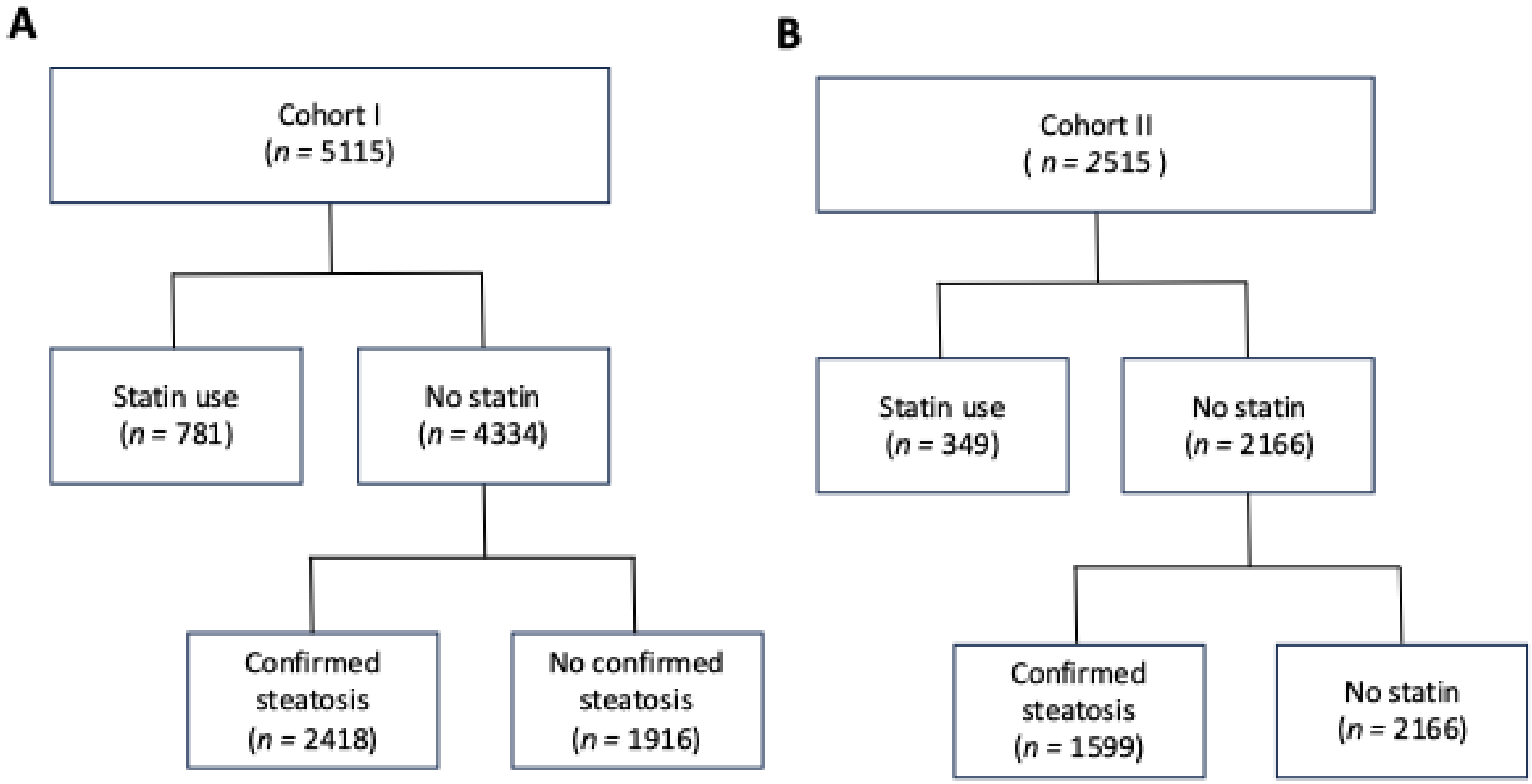

Supplementary figure legends

Fig. S1.

(A) Weekly bodyweight from day 0 through week 8. **(B-C)** Hepatic gene expression. **(D-E)** Lipid levels in distal small intestinal content. **(F)** Small intestinal gene expression. **(G)** Representative images of H&E-stained liver sections. **(H)** Principal component projection of bulk RNA sequencing data from HFC-fed *Ldlr*$^{-/-}$ mice with regular or ethanol-containing drinking water. The two components with the highest attributed variation are displayed. **(I)** Volcano plot of differentially expressed genes, indicating upregulation (right) and downregulation (left) in ethanol-consuming *Ldlr*$^{-/-}$ mice compared to controls. (**J, K**) Heatmap representation of the genes involved in cholesterol metabolism (KEGG pathway accession ko04979). **(J)** Average and **(K)** individual values for each group are presented.

Data are mean ± SEM (n = 4–11 for the HFC group and n = 5–15 for the HFC+EtOH group). mRNA was normalized to *18S* and expressed relative to HFC-fed *Ldlr*$^{-/-}$ mice on regular drinking water. Statistical significance was determined using a two-tailed unpaired Student's t-test; $^{*}p \leq 0.05$, $^{**}\ p \leq 0.0$.

Fig. S2.

(A) Heatmap depicting the expression levels of all cytokine genes from the BioMart database and **(B)** of immune-related genes in livers of HFC-fed *Ldlr*$^{-/-}$ mice with regular and ethanol-containing drinking water. Individual values for each group are presented. **(C)** Gene set enrichment analysis (Reactome pathways) based on all hepatic DEG between the two groups. **(D)** Systemic IgM, **(E)** IgG1, and **(F)** IgA concentrations.

Data are mean ± SEM (n = 4–11 for the HFC group and n = 5–19 for the HFC+EtOH group).

Fig. S3.

(A) Representative flow cytometry gating plots for hepatic Ly6C^{high} monocytes. (**B-E**) Quantification of systemic immune cell populations by flow cytometry: monocytes, B cells**,** T cells, and neutrophils. **(F)** Quantification of aortic root lesion size, calculated as the average lesion size (µm²) from nine sections throughout the aortic root. **(G)** Area under the curve (AUC) for plasma cholesterol concentrations measured at baseline, week 4 and week 8 of the dietary intervention in selected mice with comparable cholesterol exposure over time. **(H)** Average aortic root lesion size (µm² per section) in selected mice with similar cholesterol exposure during the study. **(I-Q)** 16 weeks dietary intervention. **(I)** Weekly bodyweight from day 0 through week 16. **(J)** Plasma ethanol levels at the study endpoint. **(K)** Liver weight. **(L)** Representative images of H&E-stained liver sections. **(M)** Hepatic cholesterol and **(N)** triglyceride levels. **(O)** Representative images showing Oil Red O staining of liver sections. **(P)** Quantification of hepatic monocytes, Ly6C^{high} monocytes (, B cells ($CD45^{+}B220^{+}CD3^{-}$), T cells, and neutrophils. **(Q)** Hepatic gene expression.

(A-H) Data are mean ± SEM (n = 7–11 for the HFC group and n = 9–11 for the HFC+EtOH group). (I–Q) Data are mean ± SEM (n = 5–6 for the HFC group and n = 7 for the HFC+EtOH group). mRNA was normalized to *18S* and expressed relative to HFC-fed *Ldlr*$^{-/-}$ mice on regular drinking water. Statistical significance was determined using a two-tailed unpaired Student's t test; $^{*}p \leq 0.05$, $^{**}\ p \leq 0.0$.

Fig. S4.

(A-F) 8-week dietary intervention. **(A)** 16S rRNA sequencing of fecal microbiota at the end of the study, illustrating the relative abundance of indicated bacterial genera. **(B)** Principal component analysis of 16S rRNA sequencing data from fecal bacterial microbiome. **(C)** Gene expression of tight junction genes and **(D)** of mucus barrier genes in the small intestine. **(E)** Hepatic Occludin western blot quantification with representative blots. β-Actin was used as loading control. **(F)** Systemic LPS levels. (**G-I**) 8-week dietary intervention with CONV-R and GF *Ldlr*$^{-/-}$ mice. **(G)** Body weight of CONV-R and GF *Ldlr*$^{-/-}$ mice at the study endpoint. **(H)** Plasma ethanol levels of CONV-R and GF *Ldlr*$^{-/-}$ mice. **(I)** Quantification of hepatic immune cell populations by flow cytometry: Kupffer cells, B cells, T cells, and hepatic neutrophils. **(J-L)** 8-week dietary intervention at timepoint 4 weeks. **(J)** Quantification of circulating Ly6C^{high} monocytes as a percentage of all monocytes, determined by flow cytometry. **(K)** Quantification of systemic cholesterol and **(L)** triglyceride levels. (**M-O**) 4-week dietary intervention with antibiotics-mediated microbiome depletion. **(M)** Representative images indicating successful antibiotics-mediated microbiome depletion. Anaerobic bacteria: control vs. antibiotic-treated HFC and HFC+EtOH groups (left); Aerobic bacteria: control vs antibiotic-treated HFC and HFC+EtOH groups (right). **(N)** Body weight of antibiotic-treated *Ldlr*$^{-/-}$ mice at the study endpoint. **(O)** Plasma ethanol levels of antibiotic-treated *Ldlr*$^{-/-}$ mice.

(A-F, J-K) Data are mean ± SEM (*n* = 8–12 for the HFC group and *n* = 11–12 for the HFC+EtOH group). (G–I) Data are presented as mean ± SEM (*n* = 12 for the CONV-R group and *n* = 12 for the GF group). (N, O) Data are presented as mean ± SEM (*n* = 6–7 for the antibiotic-treated HFC group *n* = 5-7 antibiotic-treated HFC+EtOH group). mRNA was normalized to *18S* and expressed relative to to HFC-fed *Ldlr*$^{-/-}$ mice on regular drinking water. Statistical significance was determined using a two-tailed unpaired Student's t test; ** $p \leq 0.01$, **$p \leq 0.001$

Fig. S5.

(A) The partial least squares discrimination analysis (PLS-DA) of the systemic metabolome profile of HFC-fed *Ldlr*$^{-/-}$ mice with regular and ethanol-containing drinking water. The two components with the highest attributed variation are displayed. **(B)** Relative abundance of key metabolites of the main component of PLS-DA in plasma of ethanol-consuming HFC-fed *Ldlr*$^{-/-}$ mice compared to controls. **(C)** Heatmap displaying the mean plasma metabolite abundance within the two experimental groups. **(D)** Heatmap representation of the genes involved in inflammasome activation (KEGG pathway accession mmu04621) in livers of mice depicted as individual values for each group.

Fig. S6.

(A) Flow chart describing Cohort I. **(B)** Flow chart describing Cohort II.

Supplementary tables

Table S1. Murine blood parameters after 16 weeks dietary intervention.

Parameter	Unit	HFC	HFC + EtOH	*P* value
**White blood cells**	10^3/µL	10.8 (2.1)	14.4 (3.5)	0.0513
**Lymphocytes**	10^3/µL	7.9 (1.5)	9.6 (2.0)	0.1018
**Lymphocytes**	%	73.0 (3.8)	67.5 (3.0)	**0.0146**
**Monocytes**	10^3/µL	0.75 (0.2)	1.1 (0.3)	**0.0451**
**Monocytes**	%	7.2 (1.1)	8.0 (0.6)	0.1232
**Granulocytes**	10^3/µL	2.2 (0.5)	3.7 (1.2)	**0.0232**
**Granulocytes**	%	19.7 (2.9)	24.4 (2.7)	**0.0124**
**Eosinophils**	%	1.9 (0.4)	2.3 (0.1)	**0.035**
**Red blood cells**	10^6/µL	15.9 (1.6)	16.0 (1.1)	0.2985
**Hemoglobin**	g/dL	24.5 (2.1)	24.6 (1.9)	0.9576
**Hematocrit**	%	83.78 (7.2)	84.3 (6.9)	0.8847
**Mean corpuscular volume**	µm	52.5 (1.5)	52.6 (1.1)	0.9244
**Mean corpuscular hemoglobin**	pg	15.4 (0.6)	15.4 (0.4)	0.7685
**Mean corpuscular hemoglobin concentration**	g/dL	29.3 (0.6)	29.2 (0.3)	0.6021
**Red blood cell distribution width**	%	15.2 (0.6)	15.1 (0.2)	0.525
**Platelets**	10^3/µL	1226 (270.3)	1165.4 (260.5)	0.6891
**Mean platelet volume**	µm^3	6.3 (0.2)	6.3 (0.1)	0.77
**Monocyte/Lymphocyte**	ratio	0.1 (0.02)	0.1 (0.01)	0.065

Statistical significance was determined using two-tailed, unpaired Student's *t*-tests.

Table S2. Characteristics of 5115 subjects of the general population including statin users (Cohort I), stratified by self-reported alcohol intake into abstainers, low consumption (<20/30g/d), moderate consumption (20-50/30-60g/d) and high consumption (>50/60g/d), respectively.

**Parameter**	**Overall (N=5115)**	**Abstinence (n=1912)**	**<20/30g/d (n=2650)**	**20/30-50/60g/d (n=507)**	**>50/60g/d (n=46)**	***P* value**
Age, years	59.4 (8.42)	60.1 (8.63)	58.6 (8.25)	60.7 (8.20)	60.5 (8.21)	**<0.001**
Male sex	2700 (52.8%)	606 (31.7%)	1727 (65.2%)	331 (65.3%)	36 (78.3%)	**<0.001**
BMI, kg/m²	27.2 (4.68)	27.7 (5.21)	26.9 (4.37)	26.7 (3.90)	28.3 (4.68)	**<0.001**
BMI ≥30 kg/m²	1200 (23.5%)	545 (28.5%)	544 (20.5%)	96 (18.9%)	15 (32.6%)	**<0.001**
Abdominal obesity	3782 (73.9%)	1473 (77.0%)	1887 (71.2%)	385 (75.9%)	37 (80.4%)	**<0.001**
Alcohol, g/week	20.0 [0-100]	0 [0-0]	80.0 [20.0-100]	280 [160-280]	500 [440-560]	**<0.001**
Diabetes	896 (17.5%)	383 (20.0%)	405 (15.3%)	97 (19.1%)	11 (23.9%)	**<0.001**
Uric acid, mg/dL	5.77 (1.53)	5.47 (1.48)	5.88 (1.50)	6.18 (1.57)	6.85 (2.19)	**<0.001**
AST, U/L	21.0 [17.0-25.0]	20.0 [17.0-24.0]	21.0 [18.0-26.0]	22.0 [18.0-28.0]	24.0 [20.0-31.0]	**<0.001**
AST xULN	0.486 [0.400-0.600]	0.514 [0.429-0.629]	0.480 [0.400-0.600]	0.514 [0.400-0.629]	0.520 [0.440-0.680]	**<0.001**
ALT, U/L	21.0 [16.0-30.0]	19.0 [15.0-28.0]	21.0 [16.0-30.3]	23.0 [17.0-34.0]	26.0 [18.0-33.0]	**<0.001**
ALT xULN	0.500 [0.371-0.680]	0.514 [0.380-0.686]	0.486 [0.371-0.660]	0.517 [0.380-0.740]	0.540 [0.429-0.720]	**0.050**
GGT, U/L	26.0 [18.0-44.0]	22.0 [16.0-35.0]	28.0 [19.0-45.0]	35.0 [22.0-59.0]	57.0 [33.0-108]	**<0.001**
GGT xULN	0.525 [0.367-0.825]	0.483 [0.350-0.750]	0.533 [0.375-0.825]	0.633 [0.433-1.09]	1.10 [0.700-1.80]	**<0.001**
Glucose, mg/dL	102 (23.9)	103 (24.3)	102 (22.8)	104 (28.1)	106 (20.6)	**0.043**
HbA1c (%)	5.58 (0.577)	5.65 (0.605)	5.53 (0.556)	5.55 (0.523)	5.59 (0.884)	**<0.001**
Cholesterol, mg/dL	222 (43.2)	220 (44.1)	222 (41.7)	228 (46.1)	229 (51.1)	**0.003**
HDL cholesterol, mg/dL	58.4 (16.6)	57.2 (15.6)	58.7 (16.9)	60.6 (18.6)	62.0 (19.6)	**<0.001**

LDL cholesterol, mg/dL	143 (39.3)	143 (39.8)	142 (38.2)	146 (41.7)	144 (49.3)	0.325
Triglycerides, mg/dL	107 [79.0-150]	107 [79.0-145]	106 [78.0-150]	116 [84.0-163]	135 [98.8-182]	**0.001**
SCORE2, points[1]	4.9 [3.0-7.4]	4.5 [2.7-6.7]	5.0 [3.0-7.5]	5.9 [3.9-8.8]	6.0 [4.7-8.2]	**<0.001**
FRS, points[2]	0.143 [0.0806-0.251]	0.127 [0.0719-0.225]	0.147 [0.0831-0.254]	0.186 [0.103-0.304]	0.204 [0.134-0.302]	**<0.001**
CRP, mg/dL	0.190 [0.100-0.360]	0.200 [0.100-0.403]	0.170 [0.100-0.310]	0.200 [0.100-0.300]	0.200 [0.100-0.500]	**<0.001**
Monocytes, %	7.30 [6.00-8.80]	6.80 [5.70-8.40]	7.50 [6.30-8.90]	7.70 [6.60-8.98]	7.15 [6.25-9.25]	**<0.001**
Monocytes, G/L	0.420 [0.330-0.520]	0.410 [0.320-0.500]	0.430 [0.340-0.540]	0.425 [0.363-0.575]	0.455 [0.320-0.688]	**0.007**
Monocyte to lymphocyte ratio	0.243 [0.191-0.318]	0.231 [0.189-0.297]	0.250 [0.191-0.329]	0.259 [0.210-0.336]	0.314 [0.220-0.387]	**0.003**

[1] Only calculated in 2576 patients aged 40-69, without diabetes and without established cardiovascular disease; [2] Only calculated in patients without established cardiovascular disease. Group comparisons used Kruskal–Wallis tests for continuous variables and chi-square tests for categorical variables; for normally-distributed variables (uric acid and total cholesterol), one-way ANOVA was used.

Table S3. Characteristics of 1916 subjects of the general population (Cohort I) with confirmed hepatic steatosis on ultrasound (n=1916) excluding statin users, stratified by self-reported alcohol intake into abstainers, low consumption (<20/30g/d), moderate consumption (20-50/30-60g/d) and high consumption (>50/60g/d), respectively.

**Parameter**	**Overall (N=1916)**	**Abstinence (n=672)**	**<20/30g/d (n=1001)**	**20/30-50/60g/d (n=213)**	**>50/60g/d (n=30)**	***P* value**
Age, years	59.6 (8.12)	60.5 (8.23)	58.8 (7.92)	60.5 (8.24)	61.7 (8.87)	**<0.001**
Male sex	1198 (62.5%)	263 (39.1%)	759 (75.8%)	154 (72.3%)	22 (73.3%)	**<0.001**
BMI, kg/m²	29.4 (4.60)	30.5 (4.97)	29.0 (4.32)	28.2 (3.92)	29.2 (5.04)	**<0.001**
BMI ≥30 kg/m²	740 (38.6%)	331 (49.3%)	337 (33.7%)	61 (28.6%)	11 (36.7%)	**<0.001**
Abdominal obesity	1633 (85.2%)	595 (88.5%)	834 (83.3%)	177 (83.1%)	27 (90.0%)	**0.002**
Alcohol, g/week	40.0 [0-112]	0 [0-0]	80.0 [20.0-100]	280 [220-300]	500 [440-560]	**<0.001**
Diabetes	392 (20.5%)	163 (24.3%)	168 (16.8%)	53 (24.9%)	8 (26.7%)	**0.002**
Uric acid, mg/dL	6.30 (1.44)	6.00 (1.37)	6.41 (1.41)	6.64 (1.44)	6.96 (2.48)	**<0.001**
AST, U/L	22.0 [18.0-28.0]	21.0 [18.0-26.0]	22.0 [19.0-28.0]	24.0 [19.0-31.0]	23.0 [19.3-28.5]	**<0.001**
AST xULN	0.514 [0.420-0.640]	0.543 [0.440-0.657]	0.486 [0.400-0.629]	0.540 [0.440-0.665]	0.493 [0.425-0.595]	**<0.001**
ALT, U/L	26.0 [19.0-36.0]	24.0 [18.0-34.0]	27.0 [20.0-37.0]	29.0 [20.0-40.0]	24.0 [17.3-30.8]	**<0.001**
ALT xULN	0.600 [0.440-0.820]	0.600 [0.457-0.800]	0.580 [0.440-0.820]	0.620 [0.480-0.900]	0.517 [0.407-0.635]	0.065
GGT, U/L	33.0 [22.0-54.0]	26.0 [19.0-42.0]	36.0 [25.0-56.0]	45.0 [28.0-74.0]	56.5 [44.3-101]	**<0.001**
GGT xULN	0.650 [0.433-1.00]	0.550 [0.400-0.867]	0.650 [0.450-1.02]	0.808 [0.550-1.24]	1.03 [0.788-1.69]	**<0.001**
Glucose, mg/dL	105 (22.0)	107 (24.2)	104 (20.1)	107 (22.8)	108 (22.7)	**0.047**
HbA1c (%)	5.65 (0.572)	5.76 (0.646)	5.59 (0.489)	5.64 (0.576)	5.63 (1.02)	**<0.001**
Cholesterol, mg/dL	225 (42.4)	220 (42.7)	226 (40.6)	235 (46.0)	235 (53.2)	**0.001**
HDL cholesterol, mg/dL	53.6 (15.1)	51.8 (12.9)	53.8 (15.0)	57.1 (19.3)	64.5 (22.2)	**<0.001**

LDL cholesterol, mg/dL	149 (38.1)	147 (38.0)	149 (36.8)	154 (41.6)	151 (52.4)	0.237
Triglycerides, mg/dL	127 [94.0-175]	124 [93.0-169]	128 [95.0-178]	133 [96.8-192]	129 [89.0-172]	0.367
SCORE2, points[1]	7.00 [4.50-10.2]	6.40 [4.20-9.70]	7.10 [4.50-10.2]	8.00 [5.15-11.4]	7.50 [5.40-12.7]	**0.007**
FRS, points[2]	0.180 [0.107-0.293]	0.158 [0.0961-0.272]	0.189 [0.114-0.292]	0.212 [0.134-0.355]	0.185 [0.133-0.281]	**<0.001**
CRP, mg/dL	0.230 [0.120-0.450]	0.290 [0.140-0.500]	0.210 [0.100-0.400]	0.200 [0.100-0.400]	0.290 [0.145-0.613]	**<0.001**
Monocytes, %	7.40 [6.25-8.80]	6.80 [5.70-8.40]	7.50 [6.40-9.08]	8.05 [6.98-9.03]	7.05 [6.08-8.35]	**<0.001**
Monocyte to lymphocyte ratio	0.248 [0.195-0.322]	0.226 [0.189-0.303]	0.263 [0.200-0.336]	0.273 [0.210-0.340]	0.244 [0.184-0.303]	**0.015**

[1] Only calculated in patients aged 40-69, without diabetes and without established cardiovascular disease; [2] Only calculated in patients without established cardiovascular disease. Group comparisons used Kruskal–Wallis tests for continuous variables and chi-square tests for categorical variables; for normally-distributed variables (uric acid and total cholesterol), one-way ANOVA was used.

Table S4. Characteristics of 2515 individuals referred to a hepatology outpatient clinic (Cohort II), stratified by self-reported alcohol intake into abstainers, low consumption (<20/30g/d), moderate consumption (20-50/30-60g/d) and high consumption (>50/60g/d), respectively.

Parameter	Overall (N=2515)	Abstinence (n=730)	<20/30g/d (n=1290)	20/30-50/60g/d (n=290)	>50/60g/d (n=205)	*P* value
Age, years	52.5 (15.2)	51.1 (15.8)	51.5 (15.1)	56.4 (14.4)	57.8 (12.4)	**<0.001**
Male sex	1422 (56.5%)	275 (37.7%)	778 (60.3%)	194 (66.9%)	175 (85.4%)	**<0.001**
BMI, kg/m²	27.3 (7.65)	27.5 (5.62)	27.2 (9.32)	26.8 (4.19)	27.2 (5.35)	0.600
BMI ≥30 kg/m²	615 (24.5%)	219 (30.0%)	294 (22.8%)	54 (18.6%)	48 (23.4%)	**<0.001**
Diabetes	361 (14.4%)	122 (16.7%)	171 (13.3%)	29 (10.0%)	39 (19.0%)	**0.007**
Uric acid, mg/dL	5.79 (1.53)	5.46 (1.47)	5.80 (1.48)	6.08 (1.63)	6.50 (1.55)	**<0.001**
AST, U/L	31.0 [24.0-44.0]	30.0 [23.0-42.0]	31.0 [24.0-42.0]	33.0 [25.0-46.0]	42.0 [31.0-67.0]	**<0.001**
ALT, U/L	39.0 [26.0-63.0]	37.0 [24.3-63.0]	39.0 [26.0-63.0]	41.5 [28.0-63.8]	44.0 [30.0-62.0]	0.228
GGT, U/L	62.0 [30.0-134]	51.5 [24.0-117]	58.0 [28.0-118]	75.0 [38.0-156]	179 [73.0-407]	**<0.001**
Glucose, mg/dL	100 (25.7)	101 (29.3)	98.9 (23.8)	101 (22.8)	106 (27.0)	**0.002**
HbA1c (%)	5.48 (0.732)	5.59 (0.883)	5.46 (0.651)	5.42 (0.548)	5.35 (0.809)	**<0.001**
Cholesterol, mg/dL	203 (48.0)	198 (48.3)	205 (45.4)	209 (46.7)	203 (61.6)	**<0.001**
HDL cholesterol, mg/dL	58.0 (19.0)	56.8 (18.5)	57.8 (18.7)	60.2 (19.3)	60.3 (22.0)	**0.019**
LDL cholesterol, mg/dL	129 (42.7)	125 (42.0)	131 (40.8)	133 (42.8)	123 (53.6)	**<0.001**
Triglycerides, mg/dL	113 [80.0-165]	109 [80.0-159]	114 [79.0-167]	116 [81.0-160]	118 [90.0-186]	0.137
CRP, mg/dL	0.170 [0.100-0.400]	0.190 [0.100-0.420]	0.150 [0.100-0.360]	0.170 [0.100-0.388]	0.200 [0.100-0.540]	**0.007**
Monocytes, G/L	0.500 [0.400-0.635]	0.490 [0.390-0.618]	0.490 [0.390-0.620]	0.530 [0.410-0.660]	0.610 [0.490-0.760]	**<0.001**
Monocyte to lymphocyte ratio	0.278 [0.218-0.366]	0.257 [0.205-0.343]	0.277 [0.217-0.358]	0.302 [0.234-0.403]	0.350 [0.264-0.482]	**<0.001**

Group comparisons used Kruskal–Wallis tests for continuous variables and chi-square tests for categorical variables; for normally-distributed variables (uric acid and total cholesterol), one-way ANOVA was used.

Table S5. Characteristics of 1599 individuals referred to a hepatology outpatient clinic (Cohort II) with hepatic steatosis as assessed by controlled attenuation parameter (CAP) ≥248dB/m, stratified by self-reported alcohol intake into abstainers, low consumption (<20/30g/d), moderate consumption (20-50/30-60g/d) and high consumption (>50/60g/d), respectively.

Parameter	Overall (N=1599)	Abstinence (n=433)	<20/30g/d (n=811)	20/30-50/60g/d (n=202)	>50/60g/d (n=153)	*P* value
Age, years	53.8 (14.2)	52.7 (14.6)	52.7 (14.2)	56.9 (14.1)	58.1 (12.1)	**<0.001**
Male sex	975 (61.0%)	180 (41.6%)	520 (64.1%)	146 (72.3%)	129 (84.3%)	**<0.001**
BMI, kg/m²	28.8 (8.64)	29.6 (5.18)	29.0 (11.0)	27.7 (3.88)	27.6 (5.51)	**0.020**
BMI ≥30 kg/m²	529 (33.1%)	182 (42.0%)	262 (32.3%)	45 (22.3%)	40 (26.1%)	**<0.001**
Diabetes	282 (17.6%)	94 (21.7%)	137 (16.9%)	24 (11.9%)	27 (17.6%)	**0.032**
Uric acid, mg/dL	6.07 (1.48)	5.77 (1.40)	6.07 (1.45)	6.30 (1.63)	6.57 (1.48)	**<0.001**
AST, U/L	33.0 [25.0-46.0]	32.0 [24.0-44.0]	32.0 [25.0-44.0]	33.0 [26.0-46.0]	43.0 [31.0-68.0]	**<0.001**
ALT, U/L	43.0 [29.0-66.0]	40.0 [27.0-65.0]	44.0 [29.0-67.0]	44.0 [32.0-66.0]	44.0 [30.0-62.0]	0.554
GGT, U/L	67.0 [35.0-145]	58.0 [29.0-120]	62.0 [35.0-122]	78.0 [38.3-159]	191 [73.0-388]	**<0.001**
Glucose, mg/dL	104 (27.7)	105 (32.9)	103 (26.3)	102 (18.5)	107 (28.3)	0.113
HbA1c (%)	5.57 (0.772)	5.71 (0.904)	5.54 (0.717)	5.49 (0.506)	5.37 (0.873)	**<0.001**
Cholesterol, mg/dL	204 (47.4)	195 (48.4)	208 (45.5)	214 (44.7)	200 (53.4)	**<0.001**
HDL cholesterol, mg/dL	54.8 (18.0)	52.3 (16.4)	53.9 (16.8)	59.0 (19.6)	61.2 (23.3)	**<0.001**
LDL cholesterol, mg/dL	131 (42.3)	125 (42.3)	134 (41.0)	138 (40.2)	121 (48.3)	**<0.001**
Triglycerides, mg/dL	129 [93.0-184]	127 [93.0-179]	133 [94.0-191]	129 [90.3-171]	119 [91.0-185]	0.538
CRP, mg/dL	0.200 [0.100-0.410]	0.220 [0.100-0.500]	0.180 [0.100-0.370]	0.190 [0.100-0.400]	0.200 [0.100-0.410]	**0.033**
Monocytes, G/L	0.520 [0.420-0.640]	0.490 [0.390-0.610]	0.510 [0.410-0.630]	0.530 [0.420-0.668]	0.600 [0.490-0.720]	**<0.001**
Monocytes, %	8.1 [6.9-9.5]	7.6 [6.4-9.0]	8.1 [7.0-9.4]	8.5 [7.2-9.9]	9.1 [7.7-10.4]	**<0.001**

Monocyte to lymphocyte ratio	0.279 [0.219-0.365]	0.248 [0.199-0.328]	0.279 [0.222-0.359]	0.304 [0.240-0.407]	0.350 [0.265-0.462]	**<0.001**

Group comparisons used Kruskal–Wallis tests for continuous variables and chi-square tests for categorical variables; for normally-distributed variables (uric acid and total cholesterol), one-way ANOVA was used.

Supplementary references

1. Semmler G, Wernly S, Bachmayer S, Leitner I, Wernly B, Egger M, et al. Metabolic Dysfunction-Associated Fatty Liver Disease (MAFLD)-Rather a Bystander Than a Driver of Mortality. J Clin Endocrinol Metab. 2021;106(9):2670-7.
2. Schonfeld M, O'Neil M, Villar MT, Artigues A, Averilla J, Gunewardena S, et al. A Western diet with alcohol in drinking water recapitulates features of alcohol-associated liver disease in mice. Alcohol Clin Exp Res. 2021;45(10):1980-93.
3. Gruber S, Hendrikx T, Tsiantoulas D, Ozsvar-Kozma M, Göderle L, Mallat Z, et al. Sialic Acid-Binding Immunoglobulin-like Lectin G Promotes Atherosclerosis and Liver Inflammation by Suppressing the Protective Functions of B-1 Cells. Cell Rep. 2016;14(10):2348-61.
4. Mieczkowski K, Bakiri L, Griss J, Wagner EF. A Sex-Specific Anti-Inflammatory Role for p62 in Psoriasis-Like Disease. J Invest Dermatol. 2025;145(11):2763-74.e11.
5. Burger K, Jung F, Baumann A, Brandt A, Staltner R, Sánchez V, et al. TNFα is a key trigger of inflammation in diet-induced non-obese MASLD in mice. Redox Biol. 2023;66:102870.
6. Brandt A, Jin CJ, Nolte K, Sellmann C, Engstler AJ, Bergheim I. Short-Term Intake of a Fructose-, Fat- and Cholesterol-Rich Diet Causes Hepatic Steatosis in Mice: Effect of Antibiotic Treatment. Nutrients. 2017;9(9).
7. Bieghs V, Van Gorp PJ, Wouters K, Hendrikx T, Gijbels MJ, van Bilsen M, et al. LDL receptor knock-out mice are a physiological model particularly vulnerable to study the onset of inflammation in non-alcoholic fatty liver disease. PLoS One. 2012;7(1):e30668.
8. Busch CJ, Hendrikx T, Weismann D, Jackel S, Walenbergh SM, Rendeiro AF, et al. Malondialdehyde epitopes are sterile mediators of hepatic inflammation in hypercholesterolemic mice. Hepatology. 2017;65(4):1181-95.
9. Hoebinger C, Rajcic D, Silva B, Hendrikx T. Chronic-binge ethanol feeding aggravates systemic dyslipidemia in Ldlr(-/-) mice, thereby accelerating hepatic fibrosis. Front Endocrinol (Lausanne). 2023;14:1148827.
10. Piollet M, Porsch F, Rizzo G, Kapser F, Schulz DJJ, Kiss MG, et al. TREM2 protects from atherosclerosis by limiting necrotic core formation. Nat Cardiovasc Res. 2024;3(3):269-82.
11. Drost HG, Paszkowski J. Biomartr: genomic data retrieval with R. Bioinformatics. 2017;33(8):1216-7.
12. Love MI, Huber W, Anders S. Moderated estimation of fold change and dispersion for RNA-seq data with DESeq2. Genome Biol. 2014;15(12):550.
13. Stephens M. False discovery rates: a new deal. Biostatistics. 2017;18(2):275-94.
14. Ignatiadis N, Klaus B, Zaugg JB, Huber W. Data-driven hypothesis weighting increases detection power in genome-scale multiple testing. Nat Methods. 2016;13(7):577-80.
15. Wu T, Hu E, Xu S, Chen M, Guo P, Dai Z, et al. clusterProfiler 4.0: A universal enrichment tool for interpreting omics data. Innovation (Camb). 2021;2(3):100141.
16. Kolberg L, Raudvere U, Kuzmin I, Vilo J, Peterson H. gprofiler2 -- an R package for gene list functional enrichment analysis and namespace conversion toolset g:Profiler. F1000Res. 2020;9.
17. Hänzelmann S, Castelo R, Guinney J. GSVA: gene set variation analysis for microarray and RNA-seq data. BMC Bioinformatics. 2013;14:7.
18. Bueschl C, Doppler M, Varga E, Seidl B, Flasch M, Warth B, et al. PeakBot: machine-learning-based chromatographic peak picking. Bioinformatics. 2022;38(13):3422-8.
19. Castellano-Escuder P, González-Domínguez R, Carmona-Pontaque F, Andrés-Lacueva C, Sánchez-Pla A. POMAShiny: A user-friendly web-based workflow for metabolomics and proteomics data analysis. PLoS Comput Biol. 2021;17(7):e1009148.

20. van den Berg RA, Hoefsloot HC, Westerhuis JA, Smilde AK, van der Werf MJ. Centering, scaling, and transformations: improving the biological information content of metabolomics data. BMC Genomics. 2006;7:142.
21. Chong J, Xia J. MetaboAnalystR: an R package for flexible and reproducible analysis of metabolomics data. Bioinformatics. 2018;34(24):4313-4.
22. Pjevac P, Hausmann B, Schwarz J, Kohl G, Herbold CW, Loy A, et al. An Economical and Flexible Dual Barcoding, Two-Step PCR Approach for Highly Multiplexed Amplicon Sequencing. Front Microbiol. 2021;12:669776.
23. Callahan BJ, McMurdie PJ, Rosen MJ, Han AW, Johnson AJ, Holmes SP. DADA2: High-resolution sample inference from Illumina amplicon data. Nat Methods. 2016;13(7):581-3.
24. Callahan BJ, Sankaran K, Fukuyama JA, McMurdie PJ, Holmes SP. Bioconductor Workflow for Microbiome Data Analysis: from raw reads to community analyses. F1000Res. 2016;5:1492.
25. Quast C, Pruesse E, Yilmaz P, Gerken J, Schweer T, Yarza P, et al. The SILVA ribosomal RNA gene database project: improved data processing and web-based tools. Nucleic Acids Res. 2013;41(Database issue):D590-6.
